# Impact of climatic, demographic and disease control factors on the transmission dynamics of COVID-19 in large cities worldwide

**DOI:** 10.1016/j.onehlt.2021.100221

**Published:** 2021-02-03

**Authors:** Soeren Metelmann, Karan Pattni, Liam Brierley, Lisa Cavalerie, Cyril Caminade, Marcus S.C. Blagrove, Joanne Turner, Kieran J. Sharkey, Matthew Baylis

**Affiliations:** a Department of Evolution, Ecology and Behaviour, Institute of Infection, Veterinary and Ecological Sciences, University of Liverpool, Brownlow Hill, Liverpool L3 5RF, UK; b Health Protection Research Unit in Emerging and Zoonotic Infections, University of Liverpool, UK; c Department of Mathematical Sciences, University of Liverpool, Peach Street, Liverpool L69 7ZL, UK; d Department of Health Data Science, Institute of Population Health, University of Liverpool, Brownlow Street, Liverpool, L69 3GL, UK; e Department of Livestock and One Health, Institute of Infection, Veterinary and Ecological Sciences, University of Liverpool, Brownlow Hill, Liverpool L3 5RF, UK; f International Livestock Research Institute, Addis Ababa, Ethiopia

**Keywords:** COVID-19, SARS-CoV-2, Climate, Demographic, Socio-economic, Epidemic response, R0, Metropolitan areas

## Abstract

Approximately a year into the COVID-19 pandemic caused by the SARS-CoV-2 virus, many countries have seen additional “waves” of infections, especially in the temperate northern hemisphere. Other vulnerable regions, such as South Africa and several parts of South America have also seen cases rise, further impacting local economies and livelihoods. Despite substantial research efforts to date, it remains unresolved as to whether COVID-19 transmission has the same sensitivity to climate observed for other common respiratory viruses such as seasonal influenza. Here, we look for empirical evidence of seasonality using a robust estimation framework. For 359 large cities across the world, we estimated the basic reproduction number (R_0_) using logistic growth curves fitted to cumulative case data. We then assess evidence for association with climatic variables through ordinary least squares (OLS) regression. We find evidence of seasonality, with lower R_0_ within cities experiencing greater surface radiation (coefficient = −0.005, *p* < 0.001), after adjusting for city-level variation in demographic and disease control factors. Additionally, we find association between R_0_ and temperature during the early phase of the epidemic in China. However, climatic variables had much weaker explanatory power compared to socioeconomic and disease control factors. Rates of transmission and health burden of the continuing pandemic will be ultimately determined by population factors and disease control policies.

## 1 Introduction

An unusual pneumonia outbreak occurred in Wuhan, Hubei province, China in December 2019. The aetiological agent was found to be a novel coronavirus, and was named Severe acute respiratory syndrome coronavirus 2 (‘SARS-CoV-2’) [1]. SARS-CoV-2 belongs to the *Betacoronavirus* genus, with high sequence homology to a wild bat coronavirus, pointing to bats as a likely natural reservoir [2]. The disease associated with it, COVID-19, has an incubation period that can range between two days and two weeks (5–7 days median), and patients with it typically present with fever, myalgia, cough, sore throat, difficulty breathing and loss of taste/smell. COVID-19 has spread globally and the pandemic remains a significant public health emergency, with global confirmed estimates approximating 70.5 million cases and 1.6 million deaths and daily incidence still rising in some countries [3].

The transmission of human coronaviruses are often correlated with colder climates, either through direct effects upon viral stability from changes in, e.g., temperature and humidity, or broader variation in host contact and immunity [4]. For example, the endemic human coronaviruses (*Alphacoronavirus*: Human coronaviruses 229E, NL63; *Betacoronavirus*: Human coronaviruses OC43, HKU1) follow established seasonal respiratory transmission in winter [4,5]. The SARS-related coronavirus and Middle East respiratory syndrome (MERS)-related coronavirus have also exhibited associations between transmission and colder temperatures [6,7]. However, whether the transmission of SARS-CoV-2 is sensitive to climate in the same way remains a significant knowledge gap [5,8]. Governments and health authorities have now faced rises in COVID-19 cases in southern hemisphere countries (e.g. Brazil, South Africa, Australia) during austral winter, and resurgence (second or third “waves”) in northern hemisphere countries during boreal winter. There are also questions regarding whether seasonally-driven patterns of infection might continue longer-term, should COVID-19 persist endemically [8,9]. Assessing evidence for seasonality and climatic effects is therefore essential to help predict (and prepare for) potential short- and long-term climate-driven disease dynamics [10].

Early attempts to capture relationships between climate and case numbers or transmission rate have produced disparate and inconclusive results. Analyses have conflictingly reported positive [[11], [12], [13]], negative [14,15], or nonlinear [16] effects of humidity. Reported effects of temperature appear more consistent, suggesting a consensus negative relationship [[11], [12], [13],^15^], or curvilinear patterns, with cases or transmission rate peaking between ~0 and ~10 °C [[16], [17], [18]]. However, these initial modelling efforts often achieved relatively poor model fits to data, either because of data specified at country or administrative division-level, ignoring heterogeneity in local climatic conditions and epidemic spread, or failure to account for additional confounding variables. A recent systematic review highlighted that 12 of 17 climatological analyses of COVID-19 did not include or discuss potential demographic or socioeconomic confounders [19]. As COVID-19 transmission has been experienced for only one year, finding rigorous evidence for seasonal sensitivity requires a carefully-considered methodology [20].

In this study, regression models are used to explore potential effects of climatic factors upon the basic reproduction number (R_0_) while adjusting for variation in demography, socioeconomics, and epidemic response. R_0_ is the number of secondary infections resulting from one infected individual in a fully susceptible population [21]. We calculate it from the rate of exponential growth during the initial period typically observed in the initial “wave” of an epidemic. The exponential growth rate is estimated by fitting the logistic equation to cumulative COVID-19 case data, which we show provides more robust results than fitting our model to incidence data. The logistic equation is able to account for the slowdown following the period of exponential growth due to limitations on the availability of new susceptible individuals. We used data for 359 large cities (population > 0.5 million) representing 43 countries in all permanently inhabited continents with a wide range of climates.

## 2 Methods

We defined a large city as having at least half a million people with population figures given by Cox [22], considering greater metropolitan areas or agglomerations of neighbouring cities as one city. We then collected case data for 374 of these large cities (Supplementary Material (SM) Table S1).

The exponential growth rate was estimated by fitting the logistic population growth model to cumulative case data. The logistic equation is numerically efficient in comparison to, for example, the Susceptible-Infectious-Recovered (SIR) model, because it avoids the need for repeatedly solving a complex differential equation system and is just as effective in representing the early epidemic behaviour [23]. We fitted cumulative data because we show here that this is a more robust method than using incidence data (SM Section S2.1).

Our algorithm (SM Section S2.2) automatically crops the data at the point of inflection where transmission is slowed down, an approach similar to that used by Hsieh and Chen [24]. The same algorithm is systematically applied to all cities to avoid bias. The basic reproduction number, R_0_, was then calculated from the exponential growth rate following Wallinga and Lipsitch [25] (SM Section S2.3). The determination of R_0_ by this method is independent of COVID-19 testing rates, except that the testing rates are assumed to be constant through the fit window. We excluded eight cities for which model fits were considered unreliable due to fitting based on fewer than five days of incidence data, plus a further seven cities as they lacked data on the initial growth phase (SM Section S4.8).

We assembled a set of covariates that potentially explain variation in estimates of R_0_, covering five broad categories: climatic, geographic, demographic, socioeconomic and epidemic response at city-level resolution (SM Section S1.2 and Table S2). Where no city-level data were available, country-level average data were substituted or imputed based on other covariates using a random forest-based procedure [26] (SM Section S3.1). Where covariates appeared inter-correlated (SM Fig. S3), we discarded a subset (preferentially retaining city-resolution covariates over country-resolution covariates, and covariates with fewer imputed values if equal resolution) until the remaining set of input covariates did not demonstrate multicollinearity (i.e., all variance inflation factor values <5).

To quantify associations between R_0_ and potential explanatory covariates, the following regression models were constructed:

1. Ordinary least squares (OLS) regression model.
2. Mixed-effects regression model: Constructed by adding country-level random intercepts and model fit compared through likelihood ratio tests (LRT).

A saturated OLS regression is initially constructed by taking the full set of covariates and reducing to a minimal model by stepwise removal based on Akaike Information Criterion (AIC) score. Model fit is then compared to a mixed-effects regression with the same covariates. Cities were assigned weights within regression models proportional to the number of days of incidence data used to calculate exponential growth, acting as a proxy for confidence in the R_0_ estimate. All models were additionally run without weights to check weighting sensitivity.

## 3 Results

**Fig. 1:**
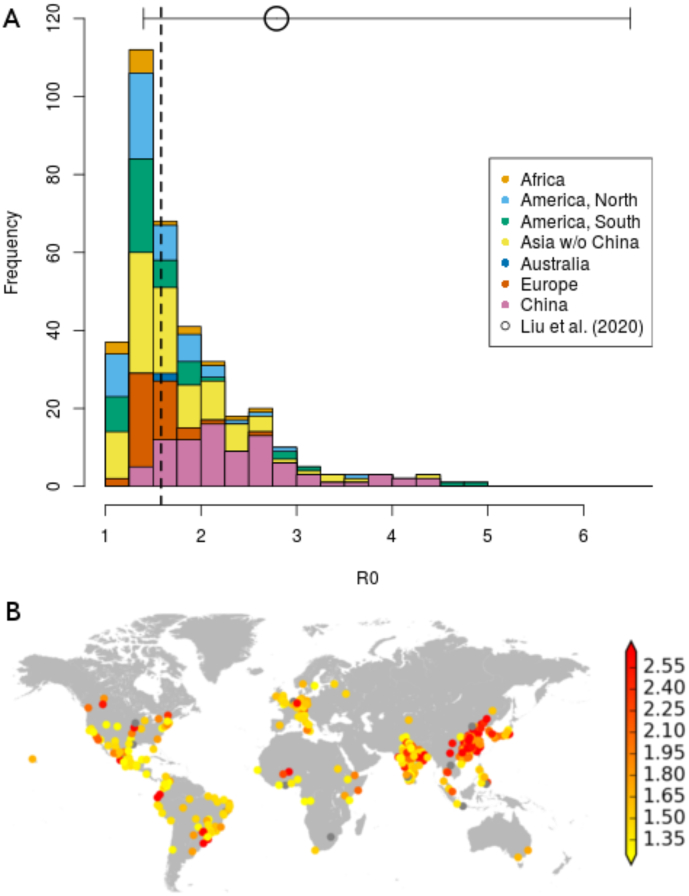
Calculated values of R_0_ over the exponential growth period for world cities with at least half a million inhabitants. A) Frequency histogram of R_0_ values calculated for 359 cities. Colour indicates continent, with China highlighted separately. Dashed line indicates median R_0_ value. The circle with horizontal bars indicates median, min and max of R_0_ values, as collected by Liu et al. [29]. B) Mapped locations of all 374 cities examined. Colour indicates R_0_ value, with grey dots indicating excluded cities (see SM Section S4.8).

Considering the 359 cities for which reliable estimates of R_0_ were obtained, Fig. 1 shows that these estimates follow a slightly skewed distribution with a median of 1.58 and interquartile range (IQR) of 0.70, with greater values for cities in China than those in other parts of the world (see also SM Table S3 for descriptive statistics by continent).

### 3.1 Regression models for global data

**Fig. 2:**
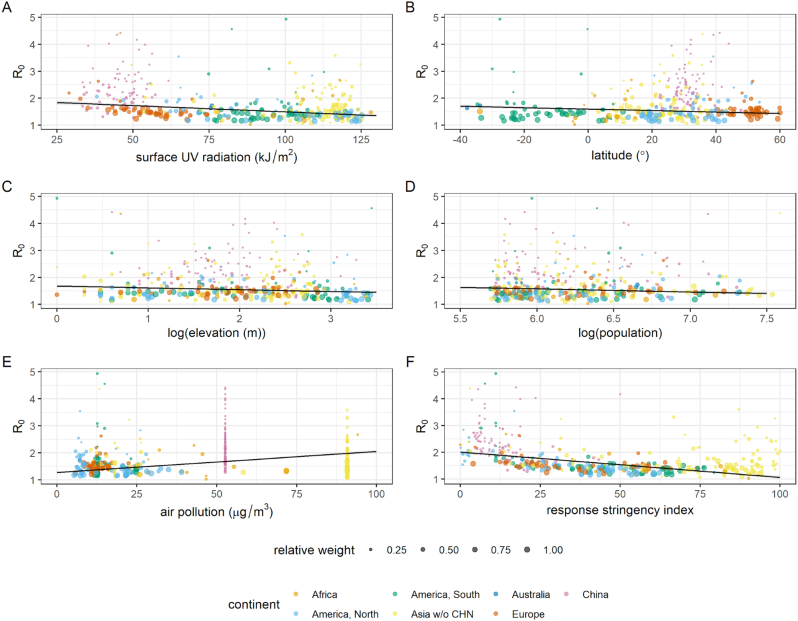
Plotted covariates from selected OLS regression model predicting R_0_ within global cities (n = 359), showing model effects significant at the 5% level: A) UV radiation, B) latitude, C) elevation, D) population size, E) mean population exposure to air pollution, and F) index measuring stringency of government response two weeks before epidemic growth period. Size of points is proportional to weighting in model, determined as number of observed days of incidence. Lines denote fits, calculated as estimated marginal means holding all other model variables constant. Shaded areas denote 95% confidence interval.

The OLS model was selected because the mixed-effects model did not significantly improve model fit (LRT statistic = 1.67, df = 1, p(LRT) = 0.196). The covariates retained include climatic, demographic, geographic, socioeconomic and response variables (Table 1, Fig. 2). They explain 33.6% (adjusted measure: 32.3%) of variance in estimated R_0_. Model fit does not appear to vary between cities in different continents (SM Fig. S4), though there is consistent under-prediction of large R_0_ values (>3), the majority of which are in cities in China (n = 12) or India (n = 3).

**Table 1:** Outputs from selected OLS regression models predicting R_0_ within global cities (n = 359) and global cities excluding China (n = 274). CI = confidence interval, ΔAIC = change in Akaike Information Criterion when term excluded, LRT = Likelihood ratio test.

	Global analysis			Global analysis excluding China		
Covariate	Coefficient (95% CI)	ΔAIC	p(LRT)	Coefficient (95% CI)	ΔAIC	p(LRT)
Relative humidity (%)	−0.004 (−0.008, 0.000)	1.49	0.062	−0.003 (−0.007, 0.001)	0.30	0.129
Surface UV radiation (kJ/m^2^)	−0.005 (−0.007, −0.002)	15.4	<0.001	−0.003 (−0.006, 0.000)	3.57	0.018
Calendar day	–	–	–	0.004 (−0.001, 0.008)	0.86	0.091
Latitude (°)	−0.003 (−0.005, −0.001)	4.69	0.010	−0.003 (−0.005, −0.001)	4.24	0.012
Log(elevation (m))	−0.067 (−0.118, −0.016)	4.81	0.009	−0.059 (−0.110, −0.007)	3.24	0.022
Log(population)	−0.113 (−0.200, −0.026)	4.57	0.010	−0.091 (−0.186, 0.005)	1.63	0.057
Log(population density (km^−2^))	–	–	–	0.167 (−0.015, 0.350)	1.38	0.066
Log(GDP per capita (USD))	–	–	–	0.160 (−0.038, 0.357)	0.64	0.105
Air pollution (μg/m^3^)	0.008 (0.006, 0.009)	83.6	<0.001	0.006 (0.004, 0.008)	25.0	<0.001
Stringency of government response	−0.009 (−0.012, −0.007)	70.1	<0.001	−0.010 (−0.013, −0.007)	39.5	<0.001

The model coefficient for ultraviolet (UV) radiation indicates that R_0_ is lower in cities with greater UV radiation reaching the surface, with an average decrease of 0.005 associated with each 1 kJ/m^2^ increase in average daily measures (Fig. 2A). Although a weak negative effect of relative humidity was retained in the selected model, this was not significant at the 5% level (Table 1).

For geographic, demographic and socioeconomic covariates, model coefficients indicate a greater R_0_ is associated with lower latitudes, lower elevation, and smaller populations experiencing greater historical air pollution exposure (Fig. 2B–E). Impact of control measures may be reflected by the strong evidence for association with mean stringency of government response two weeks prior. The model coefficient for this covariate indicates that there is an average decrease of 0.09 in R_0_ for an increase of 10 in the stringency index (measured from 0 to 100; see [27]; Fig. 2F). All model conclusions were insensitive to model weighting (SM Table S8).

### 3.2 Regression models excluding cities in China

**Fig. 3:**
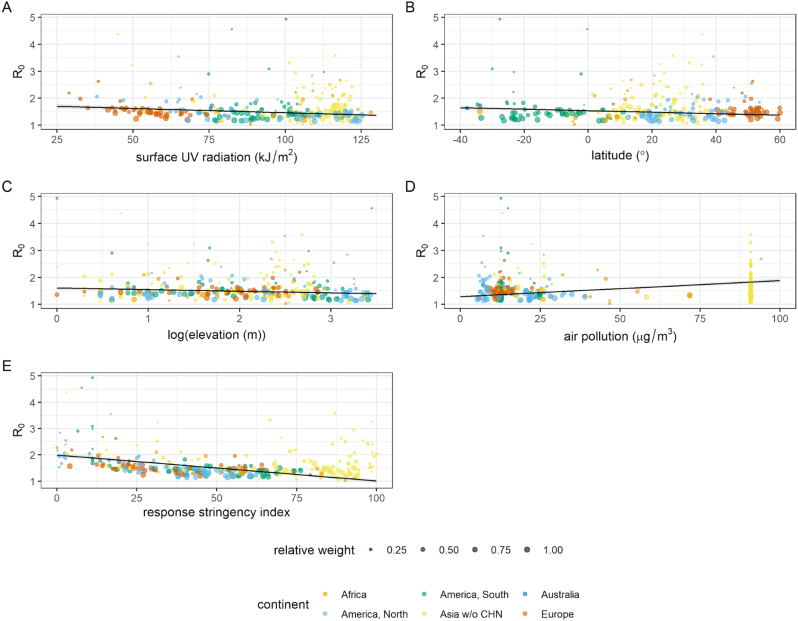
Plotted covariates from selected OLS regression model predicting R_0_ within global cities excluding China (n = 274), showing model effects significant at the 5% level: A) UV radiation, B) latitude, C) elevation, D) mean population exposure to air pollution, and E) index measuring stringency of government response two weeks before epidemic growth period. Size of points is proportional to weighting in model, determined as number of observed days of incidence. Lines denote fits, calculated as estimated marginal means holding all other model variables constant. Shaded areas denote 95% confidence interval.

As China was the centre of the initial phase of the pandemic when little was known to inform surveillance or responses and less UV radiation reached surfaces in the northern hemisphere, further regression models were constructed excluding cities in China (n = 85) to validate sensitivity of model effects (retaining n = 274 cities). The best-fitting model was again the OLS model (LRT statistic = 2.05, df = 1, p(LRT) = 0.152, see also SM Fig. S5). The effects of this model (Table 1) are comparable to those for the global dataset model; a similar negative association between UV radiation and R_0_ is observed (Fig. 3A). This model retains additional weak associations with calendar day, population density and GDP per capita, though the strongest associations again indicate R_0_ is greater in cities with a smaller population, greater air pollution and lower elevation and latitudes (Fig. 3). More stringent government responses two weeks prior is again associated with a lower R_0_ (Fig. 3H). Model conclusions were again insensitive to weighting (SM Table S9).

**Fig. 4:**
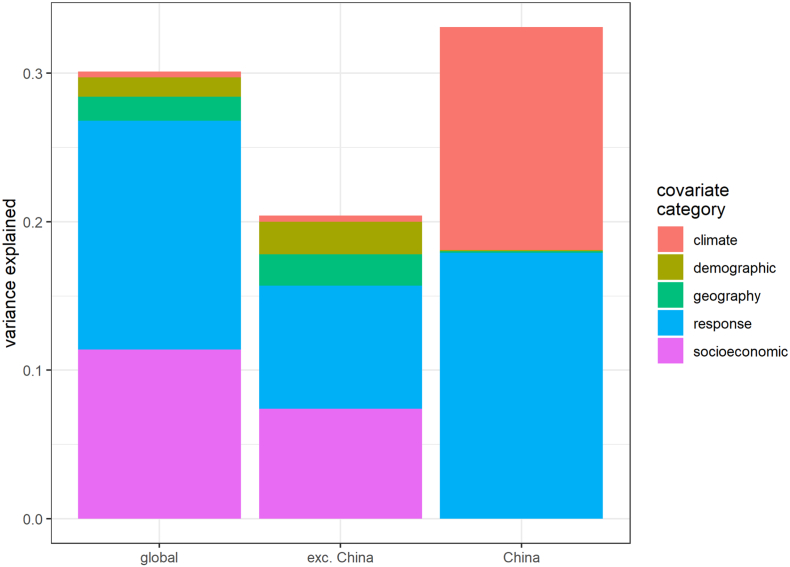
Relative importance of each covariate category in saturated OLS regression models predicting R0 within all global cities, global cities excluding China, and cities within China only. Y axis denotes collective proportion of variance explained, i.e., contribution to model R^2^. See SM Table S2 for explanation of covariate categories. No socioeconomic covariates and a limited subset of demographic covariates were modelled due to lack of data at sufficient resolution for cities in China (see SM Section S3.2).

Although overall explanatory power was limited in all cases, comparing saturated models (SM Tables S4–S6) shows climate variables only had substantial explanatory power for R_0_ when limiting analyses to cities in China only (Fig. 4), though this analysis used a limited subset of covariates. Contrastingly, socioeconomic and epidemic response covariates explained the greatest proportion of variance in R_0_ estimates for our global analyses.

## 4 Discussion

Although COVID-19 became a pandemic within a short period of time, the extent of transmission has greatly varied across large metropolitan areas worldwide (Fig. 1). We observe that the highest estimated R_0_ values at the start of the epidemic are for cities primarily within China, with several in other Asian countries and the Americas. We show that UV radiation explains a small proportion of global variation in R_0_ (Table 1). No other significant associations with climatic variables were observed, except temperature within cities in China only (SM Table S7). Finally, through the association between R_0_ and strictness of disease control (Table 1), we also report globally an estimable impact of governmental responses.

To determine the initial exponential growth rate of the epidemic in a city, we fit the logistic equation to the cumulative case data instead of daily incidence data. For the initial part of an epidemic, both data types give reliable estimates on average [28]. In our case, we argued that cumulative data gives more robust results (SM Section S2.1), and observed an upward bias in the growth rate using daily incidence data. Our R_0_ estimates obtained for China (median = 1.58, range = 1.03-4.93) are consistent, albeit slightly lower, with the meta-review study of Liu et al. [29] (median = 2.79, range = 1.40–7.23) and others [30,31].

One of the most hotly debated questions is whether transmission of COVID-19 is affected by climate and, therefore, whether R_0_ will be affected by continuing seasonal changes [5,8]. Our regression models for global data found R_0_ is negatively associated with greater UV radiation (Table 1), which varies seasonally, particularly at higher latitudes. For our global dataset, UV radiation was strongly correlated with temperature, potentially indicative of a generalised climatic effect, either directly as observed for other respiratory viruses [32], or indirectly via human behavioural factors (i.e. indoor crowding during colder, darker days) and physiological factors (i.e. impacts of colder climates on human immunity [33]). However, UV radiation is specifically hypothesised to affect COVID-19 community transmission through viral inactivation [34]. This was supported in laboratory studies that showed a faster inactivation of SARS-CoV-2 with an increase of UV intensity in artificial daylight [35]. Other analyses at grid scale-level [36] and state−/country-level [37,38] also report a significant negative association between UV radiation and COVID-19 transmission, though only later in the year in the latter analyses.

However, there is generally stronger association with epidemic responses and socioeconomic factors (Table 1). This finding offers empirical support to a recent scenario analysis using mathematical models [39] predicting the influence of climate upon COVID-19 transmission to be minimal compared to the supply of susceptibles, which is a function of epidemiological dynamics and efficacy of control measures. Furthermore, recent statistical modelling efforts also find climatic variables to be of less importance compared to, for example, the number of public health interventions [14] and airport connectivity [40]. We find the strongest socioeconomic predictor to be historical exposure to air pollution, likely a proximal indicator for more industrialised and congested population contact patterns. Additionally, other studies have argued air quality to have a more direct role in COVID-19 transmission and disease [41].

Our regression models considering only China data found a negative association between R_0_ and temperature (SM Table S7 and Fig. S6). The systematic review by [19] found similar trends, where 16 of 17 studies (e.g. [12,13,15,18]) reported increased COVID-19 incidence or transmission in colder climates. When directly comparing to those studies using regression models, our estimated temperature effect size (β = −0.046) is smaller than reported in a pooled study of East Asia ([12]: −1.050) but more similar to a country-stratified study also adjusting for sociodemographic factors ([15]: China: −0.023; USA: −0.020). The review also said 13 of 14 studies (e.g. [15,16]) reported decreased transmission in more humid conditions, though we find only tentative evidence supporting this. However, according to the review the overall quality of existing evidence for climatic associations was low.

Our study relies on six months of case data during the early stage of the epidemic with quality varying between sources. For example, there are inconsistencies between sources in China case count data [42], potentially due to differences in case definitions or testing logistics. Furthermore, due to the short timescale, the epidemic trajectory for several cities is still currently unclear, particularly those in Africa where cases may not have yet reached peak epidemic growth [3]. A more detailed analysis of climate variability would require data at interannual to decadal time scales [10], though only one large betacoronavirus outbreak has previously occurred to date. For influenza, Shaman and Lipsitch [43] have highlighted that each major pandemic (1918, 1957, 1968, and 2009) was preceded by La Niña conditions e.g. colder sea surface temperatures than average in the equatorial Pacific. This year, winter temperature conditions were close to neutral in the Pacific, but a La Niña event has developed during 2020 that seems likely to continue into boreal spring 2021 [44].

The data used in our study is biased towards countries exhibiting northern hemisphere patterns of seasonality (n = 151 cities above 30° latitude compared to n = 6 cities below −30°), thus representing a restricted part of the full climatic range of human inhabited regions. Although we focus primarily on the northern hemisphere, equal concern has been raised regarding the southern hemisphere, after significant epidemic spread in Argentina, Brazil, Colombia, and South Africa. More widely, Africa hosts 16% of the global population, yet to date accounts for just 2.3% of confirmed COVID-19 cases and 2.2% of reported deaths [3]. Higher temperature has been put forward as an explanatory hypothesis [45], which our results do not support. Although the annual range in temperature is small, equatorial Africa may yet demonstrate more complex seasonal patterns of COVID-19, from no seasonality to several epidemics per year, as shown for influenza dynamics in Kenya [46,47].

Our results support the guidelines issued by WHO and CDC public-facing guidelines to not raise expectations that the COVID-19 outbreak will significantly slow as a result of changes to weather alone. On the contrary, subsequent “waves” of cases have already occurred in most European countries which look set to peak in winter time [48]. Ultimately, our work implies that seasonal change in climate may have detectable effects upon forthcoming patterns of COVID-19 transmission, but that demographic and epidemic response factors will be much more influential. As a result, it is most prudent for policymakers to continue to focus attention on disease control measures at this stage [49].

## 5 Conclusion

The assumption that COVID-19 will follow seasonal patterns as for endemic human coronaviruses and other respiratory illnesses is not unfounded, but requires careful empirical consideration. By taking a standardised dataset of large cities, we estimate R_0_ using a robust methodology and find compelling evidence that seasonal increase in UV radiation is negatively associated with R_0_. Although we focus here on identifying and quantifying any seasonal component to COVID-19 transmission, we reiterate the importance of demography and epidemic responses in COVID-19 epidemiology. We cannot control how the seasons change, but we can control our immediate disease prevention measures and policies and we urge governments to remain vigilant and evidence-informed in all climates, particularly so during winter seasons with lower UV conditions.

## Declaration of Competing Interest

None.

## Appendix A Supplementary data

Supplementary Data Collection, Supplementary Methods, Supplementary Figures S1 - S6, Supplementary Tables S1 - S10 Image 1
