# Supplementary material for: Impact of climatic, demographic and disease control factors on the transmission dynamics of COVID-19 in large cities worldwide
Source: One Health. 2021 Feb 3;12:100221. doi: 10.1016/j.onehlt.2021.100221 (PMC7857042; doi:10.1016/j.onehlt.2021.100221)
Supplement: Supplementary file 1 — Supplementary Data Collection, Supplementary Methods, Supplementary Figures S1 - S6, Supplementary Tables S1 - S10 [file mmc1.pdf]

Supplementary Material

Soeren Metelmann, Karan Pattni, Liam Brierley, Lisa Cavalerie, Cyril Caminade, Marcus S. C. Blagrove, Joanne Turner, Kieran J. Sharkey, Matthew Baylis

S1 Data Collection

S1.1 Case Data

We collected daily case data from 374 cities and metropolitan areas from 43 countries from all inhabited continents. The data source for each country is listed in Table S1. However, data was unavailable for cities in the arid Middle East and in colder parts of the world, e.g. northern Russia, therefore our models represent a restricted part of the full climatic range of human inhabited regions.

To ensure a comparable level of infrastructure and population mixing in the locations of interest, we only used cities that had a population of at least half a million people. We further excluded cities that reported fewer than 50 cases up to 8 July 2020, to mitigate the influence of imported cases compared to local transmission.

Collected daily case data was either in the form of interval data (new cases per day, also known as daily incidence) or cumulative data (new cases per day are aggregated to previously reported cases).

For our purposes, days with zero incidence (i.e. where no new cases are recorded) are not used for model fitting. Days with zero incidence are unlikely especially once an epidemic is under way and there is community spread. Instead, it is likely that cases on days with zero incidence were reported at a later date. This is because collection of samples and lab-testing does not necessarily take place on the same day. Some countries, like the UK, match a positive test to the sample collection date. Other countries might not do this and would report after lab-testing. This possible difference between cities/countries is not a problem as long as the reporting delay, the period between the actual transmission and the reporting, is constant throughout the initial phase in each individual city. We also assume that this delay is relatively short (only a few days), so that the extracted climate variables approximately match the transmission period.

We make one final assumption: we assume that the testing rate, or rather the case detection rate, is constant during the initial outbreak period. It does not matter if the testing rate differs between cities and countries (which it will do) - our method will yield comparable basic reproduction numbers as long as the testing rate is relatively constant in each individual city.

Table S1: Case data and sources.

Country (# cities)	Source Details
Argentina (1)<br>Belgium (1)<br>Canada (5)<br>China (86)<br>Ecuador (2)<br>India (75)<br>Japan (1)<br>Kazakhstan (1)<br>Niger (1)<br>Paraguay (1)<br>Russia (2)<br>Singapore (1)<br>UK (1)	[1] Xu et al. [29]<br>https://github.com/beoutbreakprepared/nCoV2019/tree/master/latest_data
Australia (2)	[2] New South Wales Government & State Government of Victoria<br>https://data.nsw.gov.au/data/dataset/nsw-covid-19-cases-by-location-and-likely-source-of-infection/resource/2776dbb8-f807-4fb2-b1ed-184a6fc2c8aa<br>https://www.dhhs.vic.gov.au/ncov-covid-cases-by-lga-source-csv
Belgium (1)	[3] Belgian Institute for Health<br>https://epistat.wiv-isp.be/covid/
Brazil (36)	[4] Ministry for Health Brazil<br>https://opendatasus.saude.gov.br/dataset/bd-srag-2020<br>https://s3-sa-east-1.amazonaws.com/ckan.saude.gov.br/SRAG/2020/INFLUD-16-11-2020.csv
Burkina Faso (1)	[5] Ministry of Health Burkina Faso<br>https://www.sante.gov.bf/corona-virus
Chile (1)	[6] Ministry of Health, Chile<br>https://www.gob.cl/coronavirus/cifrasoficiales/
Colombia (8)	National Institute of Health, Colombia<br>https://www.ins.gov.co/Noticias/Paginas/Coronavirus.aspx
Congo, Republic of (2)	Ministry of Health, Congo<br>http://sante.gouv.cg/<br>https://twitter.com/MSPPFIFD_cg

[1] As data is incomplete, city-level data from a country is used if at least 50% of country-level cases had been reported. For this, we compared daily numbers of Xu et al. with country-level numbers of the JHU CSSE database. We discarded city-level data from day n, if country-level data dropped below 50% of official numbers on day n.

[2] Case data of Sydney, SE Sydney, SW Sydney, N Sydney, and W Sydney clustered for Sydney.

[3] We use the Brussles Arrondissement as approximate metropolitan area.

[4] Aggregated non-severe and sever cases, filtered for positive test result.

[5] Only updated until 22nd May.

[6] Individually published reports. Data on 16th June was manually adjusted by the Ministry of Health, so we only used records up to this point.

Côte d'Ivoire (1)	[7] [8] Ministry of Health and Public Hygiene http://www.sante.gouv.ci/welcome/actualites/605..690
Djibouti (1)	[9] Ministry of Health of Djibouti https://covid19.gouv.dj/test
Ethiopia (1)	[7] Ethiopian Public Health Institute https://www.ephi.gov.et/index.php/public-health-emergency/novel-corona-virus-update
Germany (15)	Robert Koch Institute https://www.rki.de/DE/Content/InfAZ/N/Neuartiges_Coronavirus/Fallzahlen.html
Ghana (1)	[7] Ministry of Health, Ghana https://ghanahealthservice.org/covid19/archive.php https://ghanahealthservice.org/covid19/ https://twitter.com/mohgovgh
India (1)	Delhi Health Bulletins https://delhifightscorona.in/health-bulletins/
Indonesia (2)	Ministry of Health of the Republic of Indonesia https://corona.jakarta.go.id/en/data-pemantauan
Italy (11)	Civil Protection https://github.com/pcm-dpc/COVID-19/tree/master/dati-province
Japan (7)	[10] Ministry of Health Labour and Welfare https://mhlw-gis.maps.arcgis.com/apps/opsdashboard/index.html#/0c5d0502bbb54f9a8dddebca003631b8
Kenya (2)	[7] Ministry of Health Kenya https://www.health.go.ke/
Malaysia (1)	Director-General of Health Malaysia https://kpkesihatan.com/
Mexico (30)	[11] Mexican Government https://datos.gob.mx/busca/dataset/informacion-referente-a-casos-covid-19-en-mexico
Netherlands (3)	National Institute for Public Health and Environment https://data.rivm.nl/covid-19/
Nigeria (2)	[7] Nigeria Centre for Disease Control https://ncdc.gov.ng/diseases/sitreps/?cat=14&name=An%20update%20of%20COVID-19%20outbreak%20in%20Nigeria
Norway (1)	Norwegian Institute of Public Health https://www.fhi.no/en/id/infectious-diseases/coronavirus/daily-reports/daily-reports-COVID19/

[7] Individually published reports.
[8] Country-level data but as of June 2020, 95% of the cases recorded are in Abidjan.
[9] Country-level data, but > 70% of population are living in Djibouti City.
[10] Only about 10000 out of 17000 cases were associated with a date and used here.
[11] Filtered for cases without recent travel history.

Pakistan (1)	Government of Pakistan<br>http://covid.gov.pk/stats/ict
Peru (4)	Ministry of Health, Peru<br>https://www.datosabiertos.gob.pe/dataset/casos-positivos-por-covid-19-ministerio-de-salud-minsa
Philippines (8)	Department of Health<br>https://ncovtracker.doh.gov.ph/<br>https://drive.google.com/drive/folders/164CQ_1lI6WZJovwULpC8zHDi9K0arkiz
Senegal (1)	[7] Ministry of Health and Social Action of Senegal<br>http://www.sante.gouv.sn/activites<br>https://cartosantesen.maps.arcgis.com/apps/opsdashboard/index.html#/d74c1c8960e1450d9ade59a8b5c9e9a7
Somalia (1)	[7] Ministry of Health, Federal Republic of Somalia<br>https://twitter.com/MoH_Somalia<br>https://www.facebook.com/MoHSomalia/<br>https://www.nomadilab.org/covid-19somalia/
South Africa (2)	[7] Gauteng Department of Health & Western Cape Government<br>https://twitter.com/GautengHealth<br>https://coronavirus.westerncape.gov.za/covid-19-dashboard
South Korea (5)	[7] Korea Centers for Disease Control and Prevention (KCDC)<br>https://www.cdc.go.kr/board/board.es?mid=a30402000000&bid=0030
Spain (1)	National Centre for Epidemiology, Spain<br>https://cnecovid.isciii.es/covid19/#documentaci%C3%B3n-y-datos
Sudan (1)	[7] Federal Ministry of Health, Sudan<br>https://twitter.com/FMOH_SUDAN<br>http://www.fmoh.gov.sd/
Sweden (1)	Public Health Agency of Sweden<br>https://www.folkhalsomyndigheten.se/smittskydd-beredskap/utbrott/aktuella-utbrott/covid-19/bekraftade-fall-i-sverige/
Thailand (4)	Department of Disease Control<br>https://data.go.th/en/dataset/covid-19-daily
UK (10)	Government of the United Kingdom<br>https://coronavirus.data.gov.uk/
USA (27)	[12] John Hopkins University CSSE COVID-19 Dataset<br>https://github.com/CSSEGISandData/COVID-19/tree/master/csse_covid_19_data

[12] We used the according county as city area.

S1.2 Covariate Data

We assembled a set of predictor covariates that may potentially explain variation in R_0 between cities, covering five broad categories: climatic, geographic, demographic, socioeconomic and epidemic response at city- or country-level resolution, depending on data availability (Table S2).

For climatic covariates, daily temperature and relative humidity data were downloaded together with altitude data from Ogimet [20], using the R package 'climate' v0.9.5 [4], taking readings from the nearest weather location according to the city's latitude and longitude coordinates, extracted from Geonames [8][13]. Hourly downward UV radiation reaching the earth's surface was extracted from the Copernicus Climate Data Store [14], using city coordinates in the same way before aggregating as daily measures.

Considering demographic and socioeconomic covariates, city population size and density were firstly taken from Demographia [3]. GDP per capita, elderly dependency ratio, and mean population air pollution exposure were obtained from the OECD Metropolitan Database [21], substituting country-level average data from alternative sources where no city-level data were available (Table S2). These data, most notably air pollution, are historical data intended to capture variability in socioeconomic infrastructure and do not reflect economic or environmental changes resulting from the pandemic (Table S2). Life expectancy was obtained from the WHO Global Health Observatory [27] and prevalence of chronic respiratory disease was obtained from the Global Burden of Disease Study [9]. Self-reported International Health Regulation (IHR) capacity was obtained from the e-SPAR tool [26].

To capture epidemic responses, we extracted changes in population activity at various types of location (e.g. retail and recreation, workplaces) from Google COVID-19 Community Mobility Reports [10]. Data describing stringency of government response were then obtained from the Oxford COVID-19 Government Response Tracker [13].

All covariates averaged or derived from daily time series source data (temperature, relative humidity, UV radiation) were calculated for each city based on its respective start and/or end dates of the data fitting window (see Section S2.2), except for changes in population activity and stringency of government response, which were calculated starting two weeks prior to this period, in order to account for potential lagged impact of disease control responses.

[13] The vast majority of weather stations were within city limits (median of 8 km distance to the city centre) and 320 of 374 weather stations were within 50 km distance of the city centre. 42 weather stations were between 50 km and 100 km away from the city centre and the remaining 12 weather stations were within a 250 km range except for Anyang in China (371 km), and Tijuana and Leon in Mexico (352 resp. 644 km).

Table S2: Extracted city or country-level covariates for use in predictive modelling of R_0, along with category, source and respective date described by data.

**Covariate**	**Definition (units)**	**Category**	**Resolution**	**Source**	**Date of data**
Population	population size	demographic	city	Cox [3]	2019
Population Density	population density (per km^2)	demographic	city	Cox [3]	2019
Temperature	mean daily temperature (°C)	climatic	city	OGIMET [20], Czernecki et al. [4]	Fitting Window[14]
Relative Humidity (RH)	mean daily relative humidity (%)	climatic	city	OGIMET [20], Czernecki et al. [4]	Fitting Window[14]
Ultraviolet (UV) radiation	downward UV radiation reaching surface (kJ/m^2)	climatic	city	Copernicus Climate Change Service [14]	Fitting Window[14]
Latitude	latitude (degrees north)	geographic	city	Geonames [8]	-
Elevation	elevation (meters above sea level)	geographic	city	OGIMET [20], Czernecki et al. [4]	-
Retail and Recreation Activity	activity at restaurants, cafes, shopping centers, theme parks, museums, libraries, and movie theaters (% change compared to baseline)	epidemic response	city, otherwise administrative division, otherwise country	Google [10]	Fitting Window with 2 weeks lag[15]
Grocery and Pharmacy Activity	activity at grocery markets, food warehouses, farmers markets, specialty food shops, drug stores, and pharmacies (% change compared to baseline)	epidemic response	city, otherwise administrative division, otherwise country	Google [10]	Fitting Window with 2 weeks lag[15]

[14] City-specific data fitting window for initial part of epidemic (see Section S2.2 for details).

[15] Starting (ending) date 14 days prior to start (end) date of city-specific data fitting window for initial part of epidemic.

Park Activity	activity at national parks, public beaches, marinas, dog parks, plazas, and public gardens (% change compared to baseline)	epidemic response	city, otherwise administrative division, otherwise country	Google [10]	Fitting Window with 2 weeks lag[15]
Transit Station Activity	activity at public transport hubs e.g. subways, stations, ports (% change compared to baseline)	epidemic response	city, otherwise administrative division, otherwise country	Google [10]	Fitting Window with 2 weeks lag[15]
Workplace Activity	activity at workplaces (% change compared to baseline)	epidemic response	city, otherwise administrative division, otherwise country	Google [10]	Fitting Window with 2 weeks lag[15]
Residential Activity	activity at places of residence (% change compared to baseline)	epidemic response	city, otherwise administrative division, otherwise country	Google [10]	Fitting Window with 2 weeks lag[15]
IHR Capacity	mean self-reported International Health Regulations capacity rating (%)	socioeconomic	country	WHO [26]	2019
GDP per capita	GDP per capita (USD, constant prices, constant PPP, base year 2015)	socioeconomic	city, otherwise country	OECD [21], World Bank [25]	2018
Air Pollution	mean population air pollution exposure to PM2.5 (μg/m^3)	socioeconomic	city, otherwise country	OECD [21], GBD [9]	2017
Elder Dependency Ratio	ratio between population 65+ years to population 15-64 years old (%)	demographic	city, otherwise country	OECD [21], CIA [2]	2018
Life Expectancy	life expectancy at birth (years)	demographic	country	WHO [27]	2016
CRD Prevalence	prevalence of chronic respiratory diseases including COPD, pneumoconiosis, asthma (%)	socioeconomic	country	GBD [9]	2017

Stringency of Government Response	mean government response stringency index aggregated over 13 indicators (scale from 0 - 100)	epidemic response	city, derived from administrative division (Brazil, UK, USA), otherwise derived from country	Hale et al. [13]	Fitting Window with 2 weeks lag[15]
DayS	Starting day of data fitting window[14]	-	city	case data	2020

All covariate data was plotted within the range of the first and last decile, see figures below.

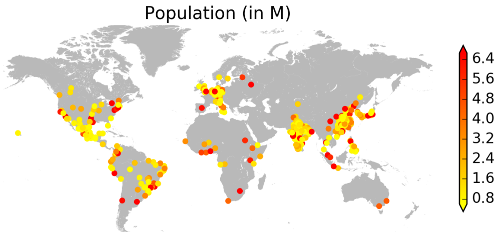

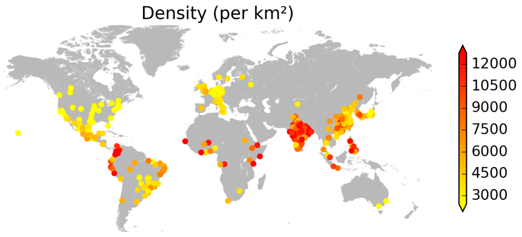

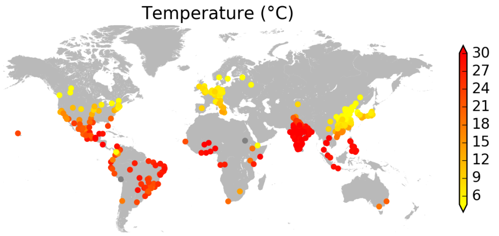

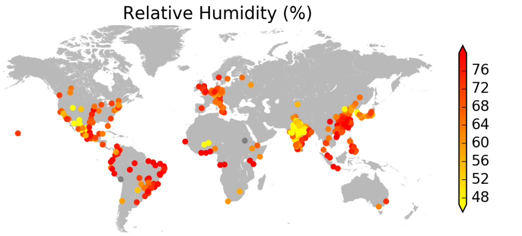

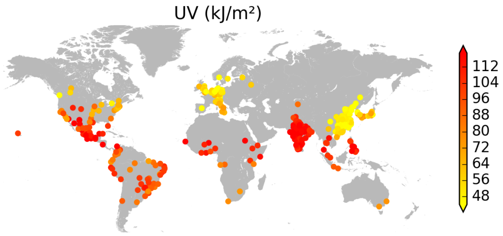

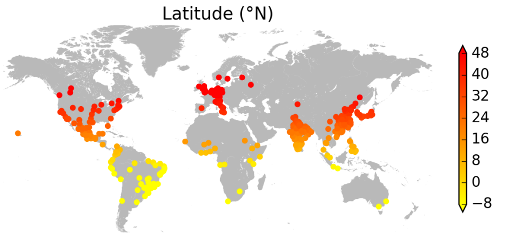

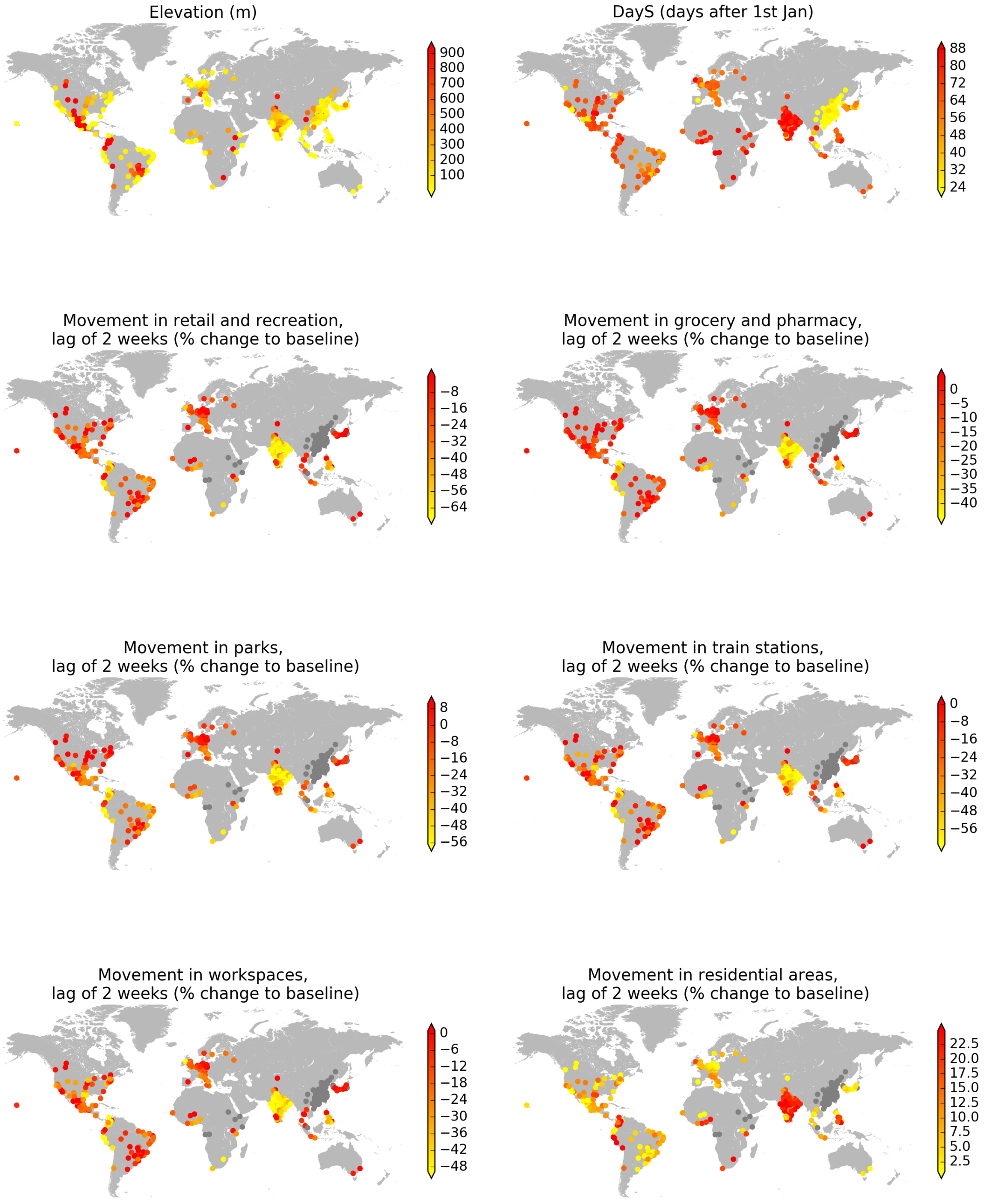
Elevation (m)
900 800 700 600 500 400 300 200 100
DayS (days after 1st Jan)
88 80 72 64 56 48 40 32 24
Movement in retail and recreation, lag of 2 weeks (% change to baseline)
−8 −16 −24 −32 −40 −48 −56 −64
Movement in grocery and pharmacy, lag of 2 weeks (% change to baseline)
0 −5 −10 −15 −20 −25 −30 −35 −40
Movement in parks, lag of 2 weeks (% change to baseline)
8 0 −8 −16 −24 −32 −40 −48 −56
Movement in train stations, lag of 2 weeks (% change to baseline)
0 −8 −16 −24 −32 −40 −48 −56
Movement in workspaces, lag of 2 weeks (% change to baseline)
0 −6 −12 −18 −24 −30 −36 −42 −48
Movement in residential areas, lag of 2 weeks (% change to baseline)
22.5 20.0 17.5 15.0 12.5 10.0 7.5 5.0 2.5

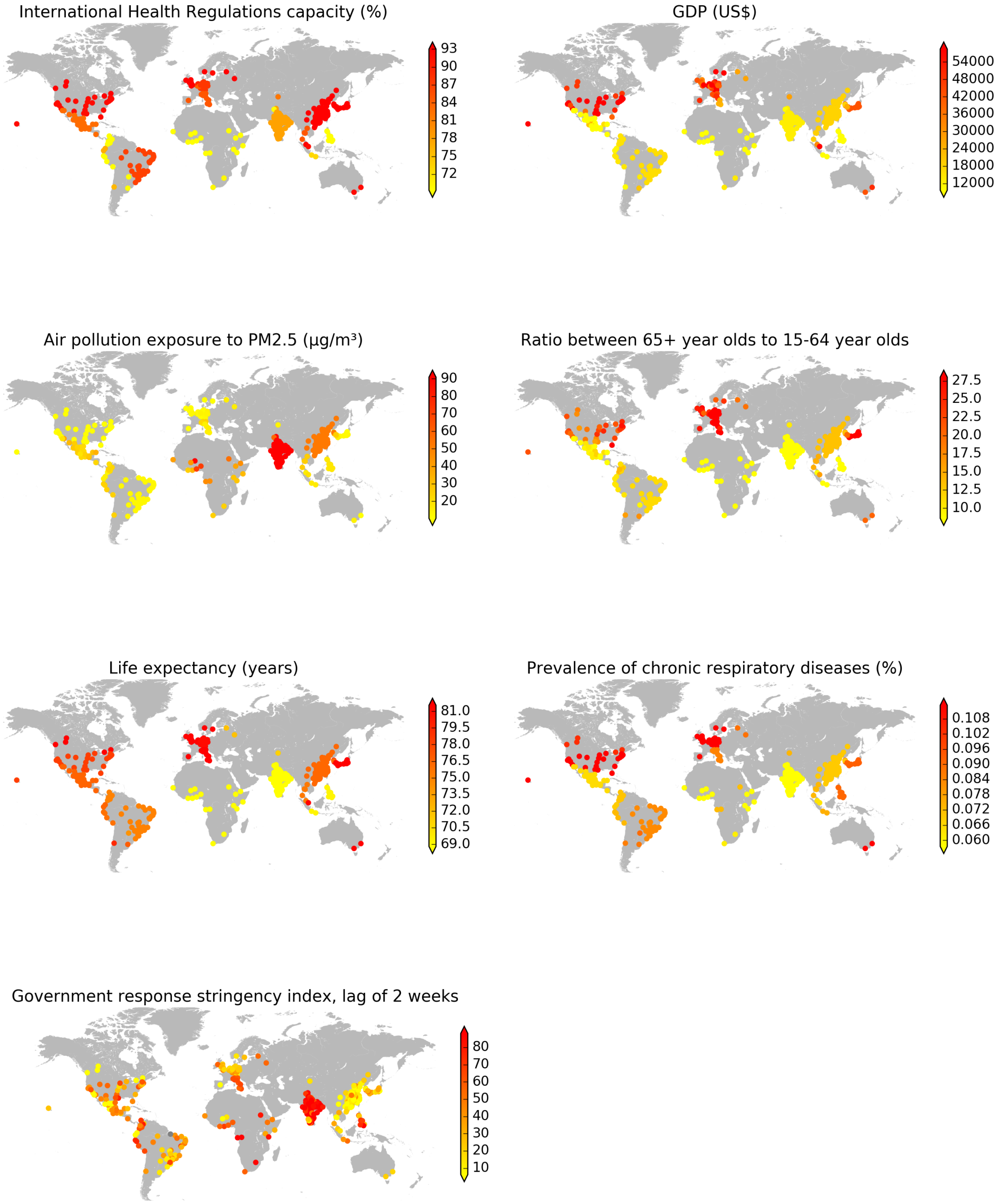
International Health Regulations capacity (%)
93 90 87 84 81 78 75 72
GDP (US$)
54000 48000 42000 36000 30000 24000 18000 12000
Air pollution exposure to PM2.5 (μg/m³)
90 80 70 60 50 40 30 20
Ratio between 65+ year olds to 15-64 year olds
27.5 25.0 22.5 20.0 17.5 15.0 12.5 10.0
Life expectancy (years)
81.0 79.5 78.0 76.5 75.0 73.5 72.0 70.5 69.0
Prevalence of chronic respiratory diseases (%)
0.108 0.102 0.096 0.090 0.084 0.078 0.072 0.066 0.060
Government response stringency index, lag of 2 weeks
80 70 60 50 40 30 20 10

S2 Methods: Determining the Exponential Growth

At least in the early stages, we observe that the majority of epidemics in large cities are typified by a rapid growth phase followed by a period in which the outbreak is being brought under control. We model this process by using the logistic equation

$$dN_t/dt = rN_t(1 - N_t/K) \tag{S2.1}$$

with solution

$$N_t = \frac{e^{rt}N_0}{1 + (e^{rt} - 1)N_0/K} \tag{S2.2}$$

where N_t is the total number of cases at time t, N_0 is the initial number of cases, r is the underlying exponential growth rate and K is a parameter representing the total number of individuals that would be infected at the end of an outbreak. This gives rise to an S-shaped curve, similar to that which is observed in the first part of the majority of data for large cities. In using this model, we are assuming that for a given city, the ratio of detected to actual cases remains constant throughout the part of the epidemic we fit to.

In general t is continuous, i.e. $t \in [0, \infty)$. For our purposes t represents a particular day in an outbreak and is considered to be discrete, i.e. $t = 1, 2, \ldots$, such that $t = 1$ is the first day of an outbreak. Let I_t denote the incidence (number of new cases) on day t, it represents the time interval $(t-1, t]$. Let C_t denote the cumulative total number of cases from day 1 to t, it represents the time interval $(0, t]$. I_t is related to C_t as follows

$$C_t = \sum_{n=1}^{t} I_n. \tag{S2.3}$$

We cannot fit the daily incidence or cumulative case data directly to the logistic equation, as the data is incomplete. In particular, the total number of cases up to and including day t is given by

$$N_t = N_0 + \sum_{n=1}^{t} I_n = N_0 + C_t, \tag{S2.4}$$

where N_0 is the number of cases prior to day 1, which have not been reported and are therefore unknown. However, from equation (S2.4), we have that I_t is related to N_t by

$$I_t = N_t - N_{t-1} \tag{S2.5}$$

and C_t is related to N_t by

$$C_t = N_t - N_0. \tag{S2.6}$$

Using these relationships, we can fit one of the two following models:

- **Model 1: Fit to daily incidence data.** This approach is used by Ma et al. [18] and Ma [17] where, using equation (S2.5), we obtain the following model where the fitted values of I_t, denoted $\hat{I}_t$, for $t = 1, 2, \ldots$ are given by

$$\hat{I}_t = \frac{e^{\hat{r}t}\hat{N}_0}{1 + (e^{\hat{r}t} - 1)\hat{N}_0/\hat{K}} - \frac{e^{\hat{r}(t-1)}\hat{N}_0}{1 + (e^{\hat{r}(t-1)} - 1)\hat{N}_0/\hat{K}} \tag{S2.7}$$

  where $\hat{r}, \hat{N}_0, \hat{K}$ are the fitted values of r, N_0, K.

- **Model 2: Fit to cumulative data.** Using equation (S2.6), the fitted values of C_t, denoted $\hat{C}_t$, for $t = 1, 2, \ldots$ are given by the model

$$\hat{C}_t = \frac{e^{\hat{r}t}\hat{N}_0}{1 + (e^{\hat{r}t} - 1)\hat{N}_0/\hat{K}} - \hat{N}_0 \tag{S2.8}$$

  where $\hat{r}, \hat{N}_0, \hat{K}$ are the fitted values of r, N_0, K.

This means that the fitted values of N_t, denoted $\hat{N}_t$, are given by

$$\hat{N}_t = \frac{e^{\hat{r}t}\hat{N}_0}{1 + (e^{\hat{r}t} - 1)\hat{N}_0/\hat{K}},$$

where $\hat{r}, \hat{N}_0, \hat{K}$ are the fitted values of r, N_0, K obtained from either model 1 or 2. Fitting to the logistic equation is numerically efficient in comparison to, for example, the SIR model, because it avoids the need for repeatedly solving a differential equation system [17]. Data fits are carried out in the R programming language using a function that implements the Levenberg-Marquardt algorithm.

S2.1 Comparison between approaches

While it is more common to fit to daily incidence data (model 1), here we fit to cumulative data (model 2). When fitting to cumulative data, errors in individual observations are correlated since they contain all cases from previous observations [18]. This results in uncertainty being underestimated leading to overconfidence in the model fit [18, 15], and is particularly problematic when trying to forecast the epidemic curve, which we are not doing. We, on the other hand, are estimating the exponential growth rate from the early phase of an outbreak. In this case, King et al. [15] showed that fitting the deterministic susceptible–infected–recovered (SIR) model to incidence or cumulative data (generated using a stochastic model) gives a fairly accurate estimate. To check that this is indeed the case, we fit the logistic equation to data generated with noise as follows.

1. Generate perfect data using the logistic equation (S2.2) with parameters r, N_0, K.

2. Generate incidence data with noise such that

$$i_t = \max\left(0, \mathcal{N}(\mu_t, \sigma_t^2)\right) \tag{S2.9}$$

   where

$$\mu_t = N_t - N_{t-1} \text{ and } \sigma_t = \epsilon\mu_t, \; \epsilon \geq 0. \tag{S2.10}$$

   That is, i_t is normally distributed random variable with mean μ_t and variance σ_t^2, but it is truncated so that $i_t \geq 0$. Note that ϵ controls the noise such that for $\epsilon = 0$ there is no noise. Calculate cumulative data using equation (S2.3), as follows

$$c_t = \sum_{n=1}^{t} i_t.$$

3. Fit models 1 and 2 up to the point of inflection, t_*, which is given by

$$t_* = \frac{\ln(K/N_0 - 1)}{r}.$$

The models are fitted to data up to t_* because, as seen later in section S2.2, we want to estimate the growth rate from the early phase of an epidemic but stop at the point where control behaviour starts to dominate over growth.

4. Store the fitted values $\hat{N_0}, \hat{r}, \hat{K}$ obtained from fitting models 1 and 2 to the data set.

5. Repeat steps 2–4 for as many realisations as required.

The results of our numerical experiment are shown in Figure S1. We see that the variance in the fitted exponential growth rate when fitting to the incidence data (model 1) is larger than when fitting to cumulative data (model 2). Furthermore, the mean has a slight upward bias in model 1 when the noise parameter (ϵ) is increased. This bias is also found to persist when the normally distributed noise (equation (S2.9)) is symmetrically truncated to be in $[0, 2\mu_t]$.

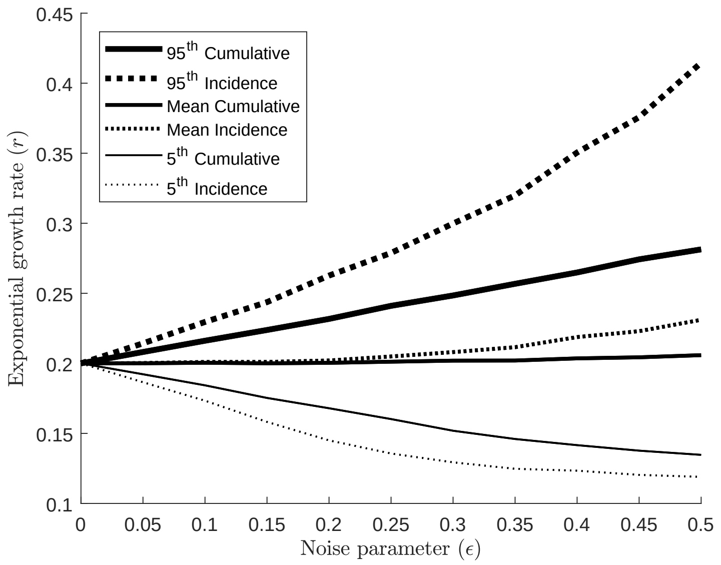

Figure S1: Comparison between fitting to incidence data (model 1) and cumulative data (model 2). Parameters used to generate the logistic growth curve are $r = 0.2, N_0 = 100, K = 10000$. Plots shown are the 5th percentile, mean and 95th percentile of the fitted exponential growth rate for 10^4 incidence curves with noise.

S2.2 Finding initial exponential growth rate:

We are interested in obtaining the underlying exponential growth rate r as a measure of the intrinsic rate of spread of the epidemic in the absence of control. The logistic equation can be seen to be

piecewise linear when plotted on the logarithmic scale (see Figure S2); that is, there are two segments consisting of an initial upward sloping line followed by a horizontal line. This is typically the case in the cumulative case curve when there is a single peak of high incidence caused by one wave of infections.

In the presence of multiple waves, the second segment would be upward sloping instead of horizontal. However, the slope of the second segment could be lower than that of the first due to control measures put in place.

For our purposes, the first segment is considered to be the first wave of an epidemic and, therefore, fitting to this part of the curve allows us to obtain the underlying exponential growth rate r we require. To find the first segment, we ensure that we crop the end point of the data before it gets too far into the control phase by determining the point of inflection when control behaviour starts to dominate over growth. Note that this is consistent with the experiment we carried out earlier where we fit to the point of inflection. We do this by using the following algorithm:

1. Let T be the last point of this interval so that $I_1, \ldots, I_T$ is the incidence data and $C_1, \ldots, C_T$ is the cumulative data. Use the correct data to fit either model 1 or 2.

2. Fit model to the data window $t \in W_m = \{1, 2, \ldots, m\}$. For our fits we have set the minimum value of m to 5. For $t \in W_m$ we define

$$\hat{N}_t(m) = \frac{e^{\hat{r}(m)t}\hat{N}_0(m)}{1 + (e^{\hat{r}(m)t} - 1)\hat{N}_0(m)/\hat{K}(m)} \tag{S2.11}$$

   where $\hat{N}_t(m), \hat{r}(m), \hat{N}_0(m), \hat{K}(m)$ are the fitted values of N_t, r, N_0, K respectively when fitting to the first m data points. Calculate the slope at point m as follows

$$\dot{N}_m(m) = \hat{r}(m)\hat{N}_m(m)\left[\hat{K}(m) - \hat{N}_m(m)\right]/\hat{K}(m). \tag{S2.12}$$

3. Repeat the previous step for all data windows with length greater than m, i.e. $W_{m+1}, \ldots, W_T$.

4. Choose the data window W_M such that the slope is largest at time point M; that is,

$$\dot{N}_M(M) = \max_{n=m}^{T}\left\{\dot{N}_n(n)\right\}. \tag{S2.13}$$

   The fit to W_M is used to give the exponential growth rate of the epidemic; that is,

$$r = \hat{r}(M). \tag{S2.14}$$

S2.3 Calculation of the basic reproduction number

The basic reproduction number (R_0[16]) is calculated following the approximation by Wallinga & Lipsitch [24] as

$$R_0 = 1 + rT \tag{S2.15}$$

[16]It is important to note that this is not the R_0 in the complete absence of control. People's behaviour is determined by factors that include government interventions and people's natural avoidance of infection. We are determining R_0 for the system under study; that is, the number of secondary cases per primary in an uninfected population and the behaviour of that uninfected population is a key factor in this quantity.

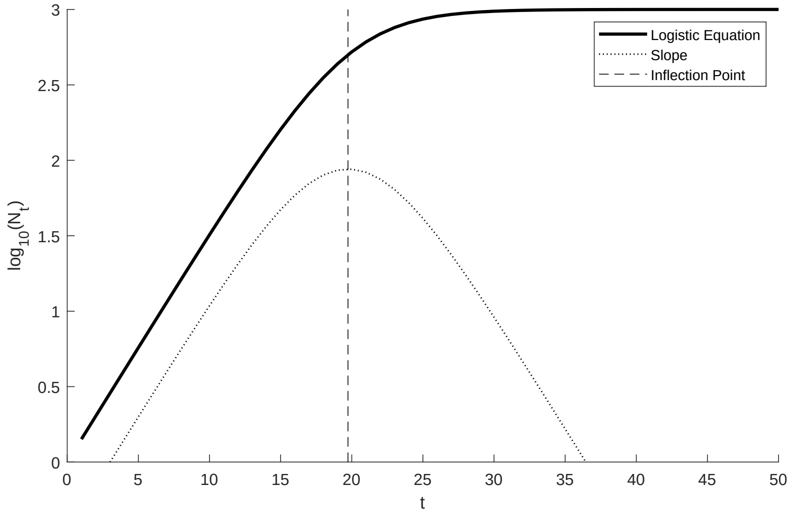

Figure S2: Logistic equation with $N_0 = 1, r = 0.35, K = 1000$ plotted on the logarithmic scale. The slope and inflection point (where slope is maximum) are also shown.

where the serial interval, T, is estimated to be 4.7 days [19]. Earlier estimates of T were in the range of 7-8 days [16, 28], resulting in higher R_0 values. More recent studies give lower serial intervals between 3.95 for China to 5.2 for Singapore [7, 5].

S2.4 R0 values

Table S3: Descriptive statistics for values of R_0 over exponential growth period for 359 world cities with at least half a million inhabitants, stratified by region. IQR = interquartile range.

**Continent**	**Number of cities**	**Min**	**Median**	**Max**	**IQR**
China	85	1.28	2.21	4.42	0.85
Australia	2	1.63	1.68	1.73	0.05
Asia (exc. China)	103	1.07	1.56	4.38	0.67
Europe	46	1.20	1.49	2.63	0.20
South America	52	1.16	1.45	4.93	0.31
North America	56	1.15	1.43	3.55	0.41
Africa	15	1.03	1.34	2.68	0.58
World	359	1.03	1.58	4.93	0.70

A total of 15 cities were excluded due to the following reasons:

1. The fitting algorithm did not work due to too few data points, jumps or local maxima in the cumulative data (8 cities: Beijing, Richmond, Houston, Virginia Beach, Wichita, Amsterdam, Yogjakarta, Des Moines).

2. There were too few data points within the first 60 days (5 cities: Aracaju, Thrissur, Aguascalientes, General Santos City, Udon Thani).

3. The start of the outbreak was not recorded (2 cities: Accra, Johannesburg).

S3 Statistical analysis

All data handling and analysis was conducted using R v3.6.1 [22]. Supporting data and code is available at https://github.com/lbrierley/metelmann_covid19_climate.

S3.1 Data handling

To avoid inflated error estimates in predictive models, we firstly examined correlation between all potential covariates (Figure S3) and retained a subset without multicollinearity for model inclusion, defined as all variance inflation factors (VIF) < 5), calculated using R package 'car', v3.0-7 [6]. When determining which covariates to retain, we preferentially retained the covariate(s) with higher resolution (i.e. data at city- or administrative division-resolution over country-resolution) and fewer missing data values. We therefore retained UV radiation while excluding temperature; stringency of government responses while excluding population activity covariates; and GDP per capita and exposure to air pollution while excluding population life expectancy, IHR capacity, and prevalence of chronic respiratory disease.

Missing values in city-level air pollution ($n = 237$: all cities within Argentina, Brazil, Burkina Faso, China, Congo, Cote d'Ivoire, Djibouti, Ecuador, Ethiopia, India, Indonesia, Kazakhstan, Kenya, Malaysia, Niger, Nigeria, Pakistan, Paraguay, Peru, Philippines, Russia, Senegal, Singapore, Somalia, South Africa, Sudan, Thailand; plus Honolulu, USA) and elder dependency ratio ($n = 237$: as for air pollution), and GDP per capita ($n = 282$: as for air pollution, plus all cities within Canada, Colombia, Japan; Bergamo, Italy; and Acapulco, Cancun, Chihuahua, Ciudad Juarez, Culiacan, Durango, Hermosillo, Leon, Mexicali, Morelia, Oaxaca, Puebla, Reynosa, Saltillo, San Luis Potosi, Tampico, Tijuana, Toluca, Torreon, Tuxtla Gutierrez, Veracruz, Villahermosa, and Xalapa, Mexico) were substituted with country-level data. Missing values in temperature ($n = 5$: Medellin, Colombia; Djibouti, Djibouti; Erode, India; Arequipa, Peru; Khartoum, Sudan), relative humidity ($n = 3$: Erode, India; Arequipa, Peru; Khartoum, Sudan), and stringency of government response ($n = 1$: Belem, Brazil) were imputed based on other covariates before modelling following a random forest-based procedure using R package 'missForest' v1.4 [23]. No other covariates selected for model inclusion had missing values. Covariates exhibiting overdispersion (population, population density, elevation, GDP per capita) were subject to a $\log_{10}(x)$ transformation prior to modelling, adjusting $\log_{10}(\text{elevation}) = 0$ for cities below sea level (Amsterdam and Rotterdam, Netherlands).

S3.2 Regression modelling

To test whether our hypothesised covariates predicted R_0 values, linear OLS regression models were initially constructed featuring all covariates, before reducing down to a final selected model by stepwise removal and retaining the model with the minimal AIC score. To minimise the influence of cities for which R_0 estimates may have greater uncertainty, all regression models weighted cities proportionally to the number of data points with non-zero incidence in their data fitting window (W_M, see Section S2.2). As unexplained residual variation may be correlated within-country, we then tested whether adding country-level random intercepts in a mixed-effects approach significantly improved model fits using likelihood ratio tests. Mixed-effects regression models were fitted using R package 'lme4' v.1.1-23 [1]. All model fits were examined by plotting residuals against fitted values and theoretical quantiles, and by examining Cook's distance plots.

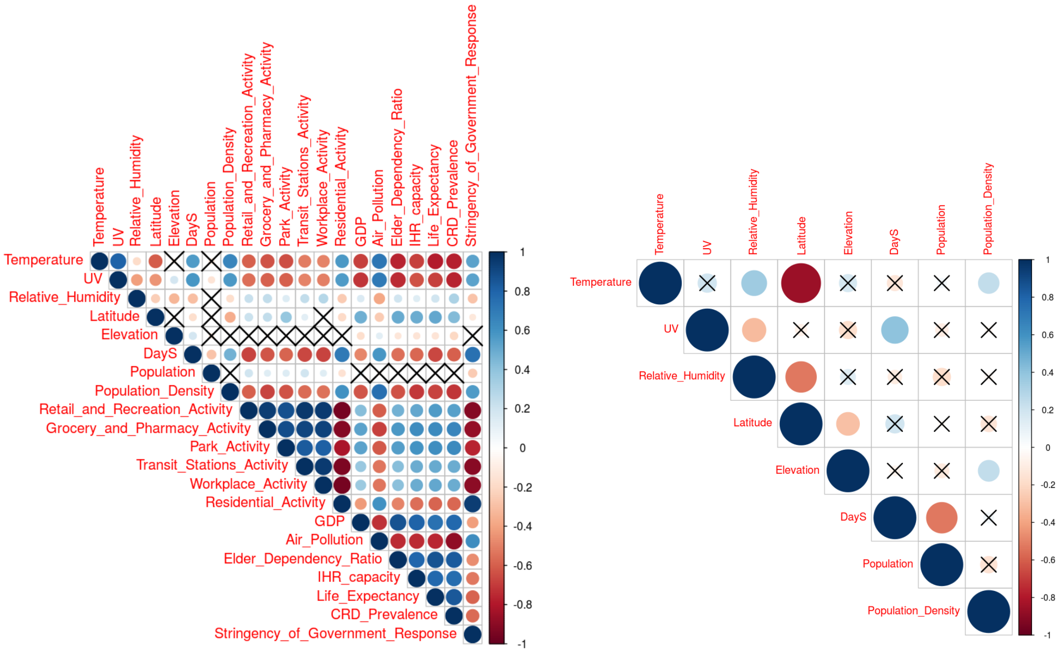

Figure S3: Cross correlation matrices, colour code indicates Spearman's correlation coefficient. Crossed out cells indicate no statistical significance ($p > 0.05$). Left: covariate data for cities outside China. Activity indices show a strong correlation with each other, so do socioeconomic indices. Right: covariate data for cities inside China for which activity and socioeconomic data was not available on city level.

Significance of covariates was determined in finalised models through likelihood ratio tests (LRTs). To additionally quantify the relative overall importance of climatic covariates, we also calculated total contribution to R^2 for each covariate type using proportional marginal variance decomposition [12] using R package 'relaimpo' v2.2-3 [11].

Before constructing the models for cities in China ($n = 83$, excluding Xinyang and Yichun based on excessive influence over model fits determined by Cook's distance), we discarded the following covariates with either country-level substituted data (i.e. constant values) or multicollinearity: latitude, elder ratio, GDP per capita, air pollution and change in retail and recreation (see Tables S6 and S7). In the minimal OLS model, R_0 was associated with only three covariates representing climate and epidemic response which collectively explained 34.5% (adjusted measure: 32.0%) of variation. Lower rates of transmission were observed for warmer climates within China, with R_0 decreasing by an average of 0.46 for every 10°C increase (Figure S6A). We also observe evidence for lower R_0 in cities with more stringent government responses two weeks prior (Figure S6C).

Finally, we repeated all regression modelling procedures without weighting cities by available incidence data, and confirmed similar resulting model effects and coefficients for the global analysis of all cities and excluding China (see Tables S8 and S9), and the model for cities in China only (Table S10).

Table S4: Outputs from initial saturated OLS regression model predicting R_0 within global cities ($n = 359$). CI: confidence interval, ΔAIC: change in Akaike Information Criterion when term excluded, LRT: Likelihood ratio test.

**Covariate**	**Coefficient** (95% **CI**)	**ΔAIC**	$\mathbb{P}$(**LRT**)
Relative Humidity (%)	$-0.004\ (-0.008, 0)$	1.493	0.062
Surface UV radiation (kJ/m^2)	$-0.005\ (-0.007, -0.002)$	9.229	0.001
Calendar day	$0.001\ (-0.003, 0.005)$	-1.624	0.54
Latitude	$-0.003\ (-0.005, -0.001)$	4.14	0.013
log(Elevation (m))	$-0.065\ (-0.116, -0.013)$	4.129	0.013
log(Population)	$-0.124\ (-0.215, -0.033)$	5.283	0.007
log(Population Density per km^2)	$0.137\ (-0.044, 0.319)$	0.282	0.131
log(GDP per capita (USD))	$0.079\ (-0.128, 0.287)$	-1.413	0.443
Air Pollution (μg/m^3)	$0.008\ (0.005, 0.01)$	44.766	< 0.001
Elder Dependency Ratio (%)	$0\ (-0.007, 0.008)$	-1.991	0.925
Stringency of government response	$-0.01\ (-0.013, -0.007)$	43.075	< 0.001

Table S5: Outputs from initial saturated OLS regression model predicting R_0 within global cities excluding China ($n = 274$). CI: confidence interval, ΔAIC: change in Akaike Information Criterion when term excluded, LRT: Likelihood ratio test.

**Covariate**	**Coefficient** (95% **CI**)	**ΔAIC**	$\mathbb{P}$(**LRT**)
Relative Humidity (%)	$-0.003\ (-0.007, 0.001)$	-0.24	0.185
Surface UV radiation (kJ/m^2)	$-0.002\ (-0.005, 0.001)$	0.765	0.096
Calendar day	$0.004\ (0, 0.008)$	1.157	0.076
Latitude	$-0.003\ (-0.005, -0.001)$	5.346	0.007
log(Elevation (m))	$-0.055\ (-0.107, -0.003)$	2.472	0.034
log(Population)	$-0.08\ (-0.177, 0.018)$	0.712	0.1
log(Population Density per km^2)	$0.166(-0.017, 0.349)$	1.334	0.068
log(GDP per capita (USD))	$0.13\ (-0.075, 0.334)$	-0.376	0.203
Air Pollution (μg/m^3)	$0.006\ (0.004, 0.009)$	26.247	< 0.001
Elder Dependency Ratio (%)	$0.004\ (-0.003, 0.012)$	-0.79	0.271
Stringency of government response	$-0.01\ (-0.013, -0.007)$	40.54	< 0.001

Table S6: Outputs from initial saturated OLS regression model predicting R_0 within cities in China ($n = 83$). CI: confidence interval, ΔAIC: change in Akaike Information Criterion when term excluded, LRT: Likelihood ratio test.

**Covariate**	**Coefficient** (95% **CI**)	Δ**AIC**	$\mathbb{P}$(**LRT**)
Temperature (°C)	-0.047 $(-0.081, -0.012)$	5.917	0.005
Relative Humidity (%)	-0.004 $(-0.027, 0.018)$	-1.827	0.677
Surface UV radiation (kJ/m^2)	0.006 $(-0.017, 0.028)$	-1.736	0.607
Calendar day	0.049 $(-0.032, 0.131)$	-0.384	0.204
log(Elevation (m))	-0.039 $(-0.29, 0.212)$	-1.892	0.742
log(Population)	0.049 $(-0.348, 0.446)$	-1.932	0.794
log(Population Density per km^2)	0.091 $(-0.782, 0.965)$	-1.951	0.825
Stringency of government response	-0.039 $(-0.056, -0.022)$	18.516	< 0.001

Table S7: Outputs from selected OLS regression model predicting R_0 within cities in China ($n = 83$) based on stepwise reduction from saturated model using AIC. CI: confidence interval, ΔAIC: change in Akaike Information Criterion when term excluded, LRT: Likelihood ratio test.

**Covariate**	**Coefficient** (95% **CI**)	Δ**AIC**	$\mathbb{P}$(**LRT**)
Temperature (°C)	-0.046 $(-0.070, -0.022)$	12.1	< 0.001
Calendar day	0.051 $(-0.012, 0.113)$	0.68	0.101
Stringency of government response	-0.040 $(-0.056, -0.024)$	20.5	< 0.001

Table S8: Outputs from selected unweighted OLS regression model predicting R_0 within global cities ($n = 359$) based on stepwise reduction from saturated model using AIC. CI: confidence interval, ΔAIC: change in Akaike Information Criterion when term excluded, LRT: Likelihood ratio test.

**Covariate**	**Coefficient** (95% **CI**)	Δ**AIC**	$\mathbb{P}$(**LRT**)
Relative Humidity (%)	-0.007 $(-0.014, -0.001)$	4.05	0.014
Surface UV radiation (kJ/m^2)	-0.007 $(-0.011, -0.004)$	11.871	0
Calendar day	0.005 $(0, 0.01)$	2.362	0.037
Latitude	-0.004 $(-0.008, 0)$	2.793	0.029
log(Elevation (m))	-0.085 $(-0.164, -0.006)$	2.573	0.032
log(Population)	-0.118 $(-0.258, 0.022)$	0.826	0.093
log(Population Density per km^2)	0.205 $(-0.061, 0.471)$	0.348	0.125
Air Pollution (μg/m^3)	0.009 $(0.006, 0.012)$	36.146	< 0.001
Stringency of government response	-0.014 $(-0.018, -0.01)$	46.68	< 0.001

Table S9: Outputs from selected unweighted OLS regression model predicting R_0 within global cities excluding China ($n = 274$) based on stepwise reduction from saturated model using AIC. CI: confidence interval, ΔAIC: change in Akaike Information Criterion when term excluded, LRT: Likelihood ratio test.

**Covariate**	**Coefficient** (95% **CI**)	Δ**AIC**	$\mathbb{P}$(**LRT**)
Relative Humidity (%)	$-0.005(-0.011, 0.001)$	0.929	0.087
Surface UV radiation (kJ/m^2)	-0.005 $(-0.009, -0.001)$	3.006	0.025
Calendar day	0.007 $(0.002, 0.013)$	4.62	0.01
Latitude	-0.005 $(-0.009, -0.001)$	5.45	0.006
log(Elevation (m))	-0.071 $(-0.149, 0.006)$	1.424	0.064
log(Population)	-0.125 $(-0.277, 0.027)$	0.736	0.098
log(Population Density per km^2)	0.317 $(0.031, 0.603)$	2.921	0.027
log(GDP per capita (USD))	0.266 $(-0.01, 0.542)$	1.737	0.053
Air Pollution (μg/m^3)	0.008 $(0.005, 0.011)$	21.106	< 0.001
Stringency of government response	-0.015 $(-0.019, -0.011)$	52.015	< 0.001

Table S10: Outputs from selected OLS unweighted regression model predicting R_0 within cities in China ($n = 83$) based on stepwise reduction from saturated model using AIC. CI: confidence interval, ΔAIC: change in Akaike Information Criterion when term excluded, LRT: Likelihood ratio test.

**Covariate**	**Coefficient** (95% **CI**)	Δ**AIC**	$\mathbb{P}$(**LRT**)
Temperature (°C)	-0.049 $(-0.074, -0.023)$	11.9	< 0.001
Calendar day	0.047 $(-0.019, 0.112)$	0.069	0.150
Stringency of government response	-0.040 $(-0.058, -0.022)$	16.3	< 0.001

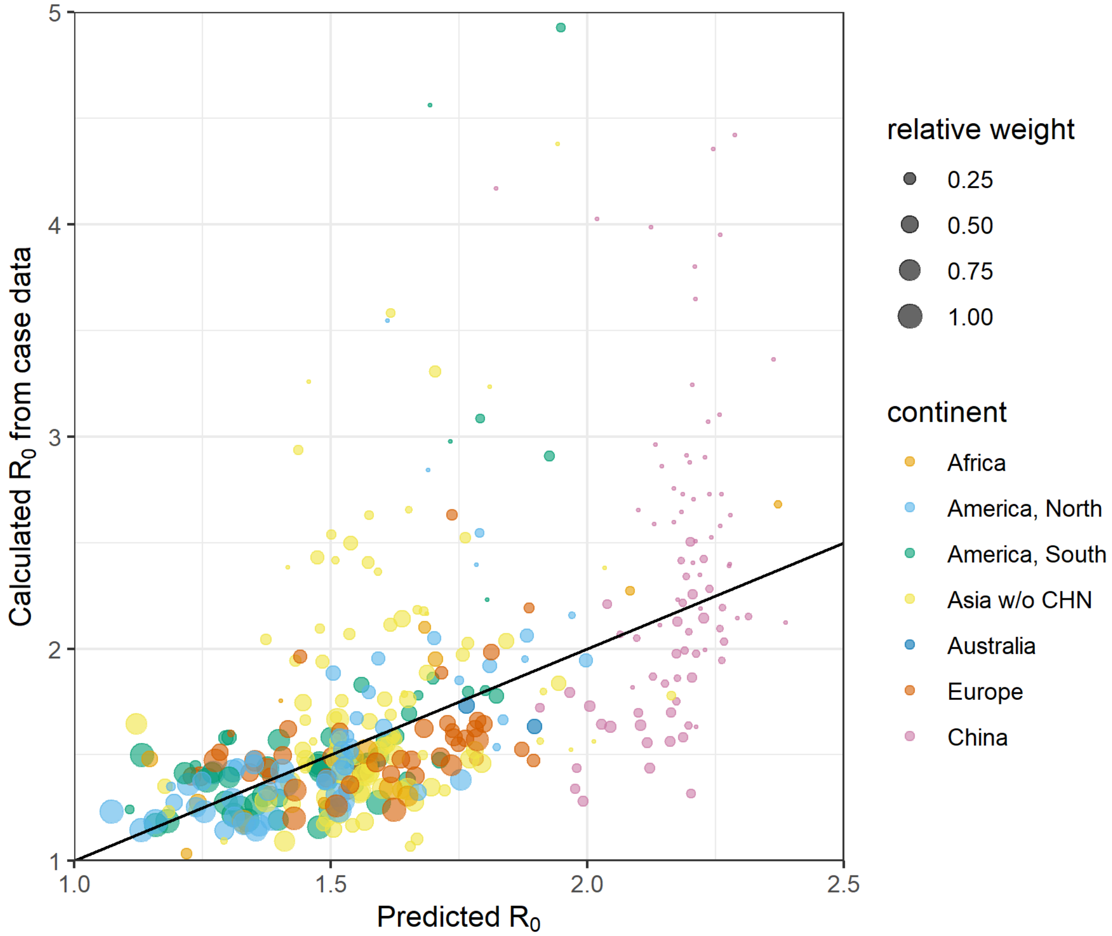

Figure S4: Plotted model performance for selected OLS regression model predicting R_0 in global cities ($n = 359$) based on climate, demographic and epidemic response covariates. Size of points is proportional to weighting in model, determined as number of observed available days of incidence. Cities above diagonal have under-estimated R_0, while those below have over-estimated R_0.

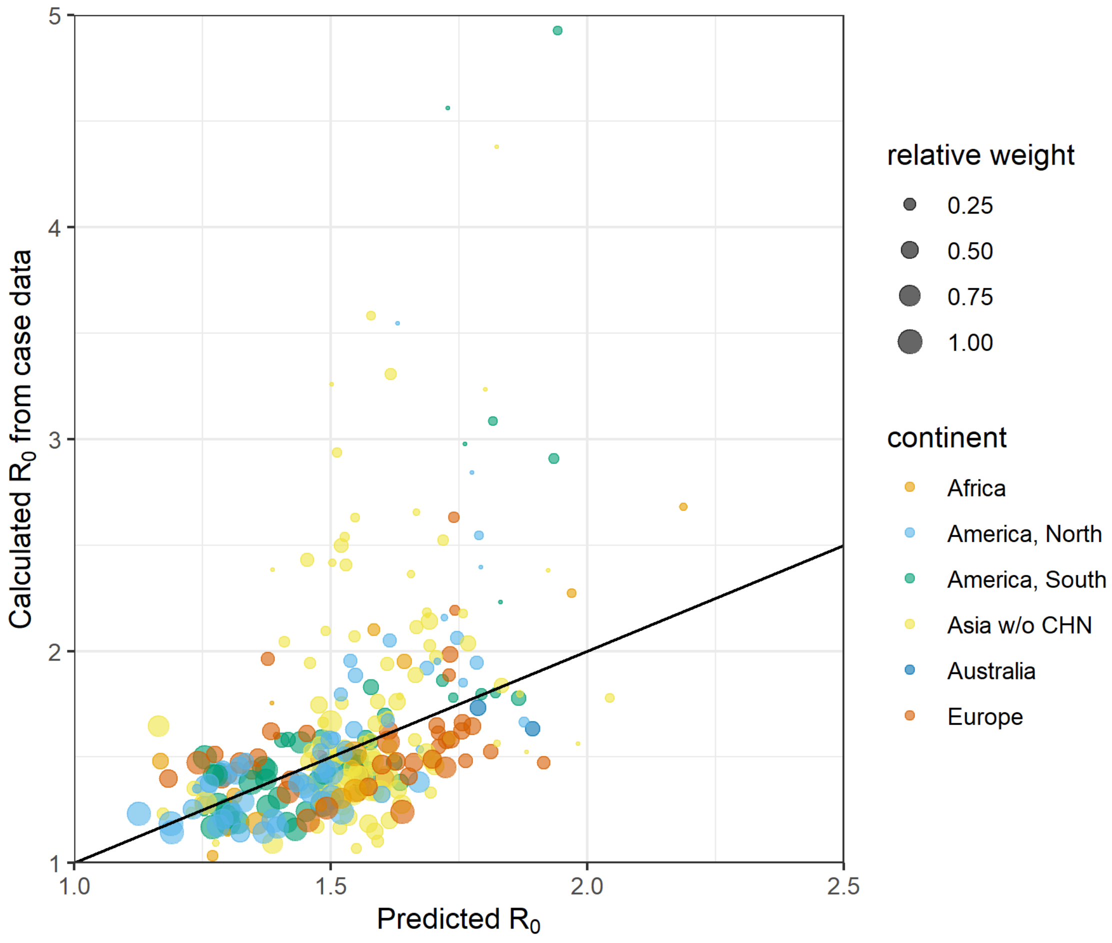

Figure S5: Plotted model performance for selected mixed-effects regression model predicting R_0 in global cities excluding China ($n = 274$) based on demographic and epidemic response covariates. Size of points is proportional to weighting in model, determined as number of observed available days of incidence. Cities above diagonal have under-estimated R_0, while those below have over-estimated R_0.

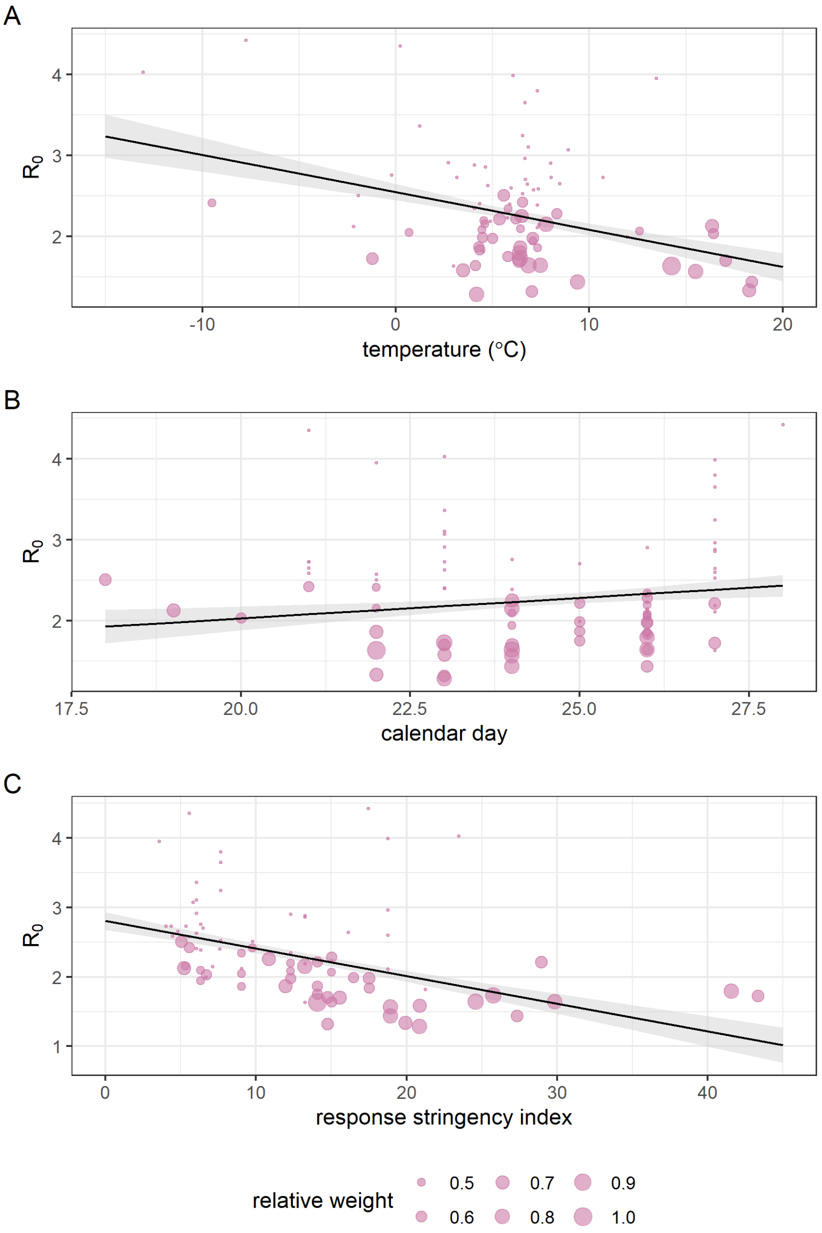

Figure S6: Plotted covariates from selected regression model predicting R_0 within cities in China ($n = 83$), showing effect of A) mean daily temperature, B) calendar day of start of fitting window, and C) index measuring stringency of government response two weeks before epidemic growth period. Lines denote fits, calculated as estimated marginal means holding all other model variables constant. Shaded areas denote 95% confidence interval.

S4 City data and fits

S4.1 Africa

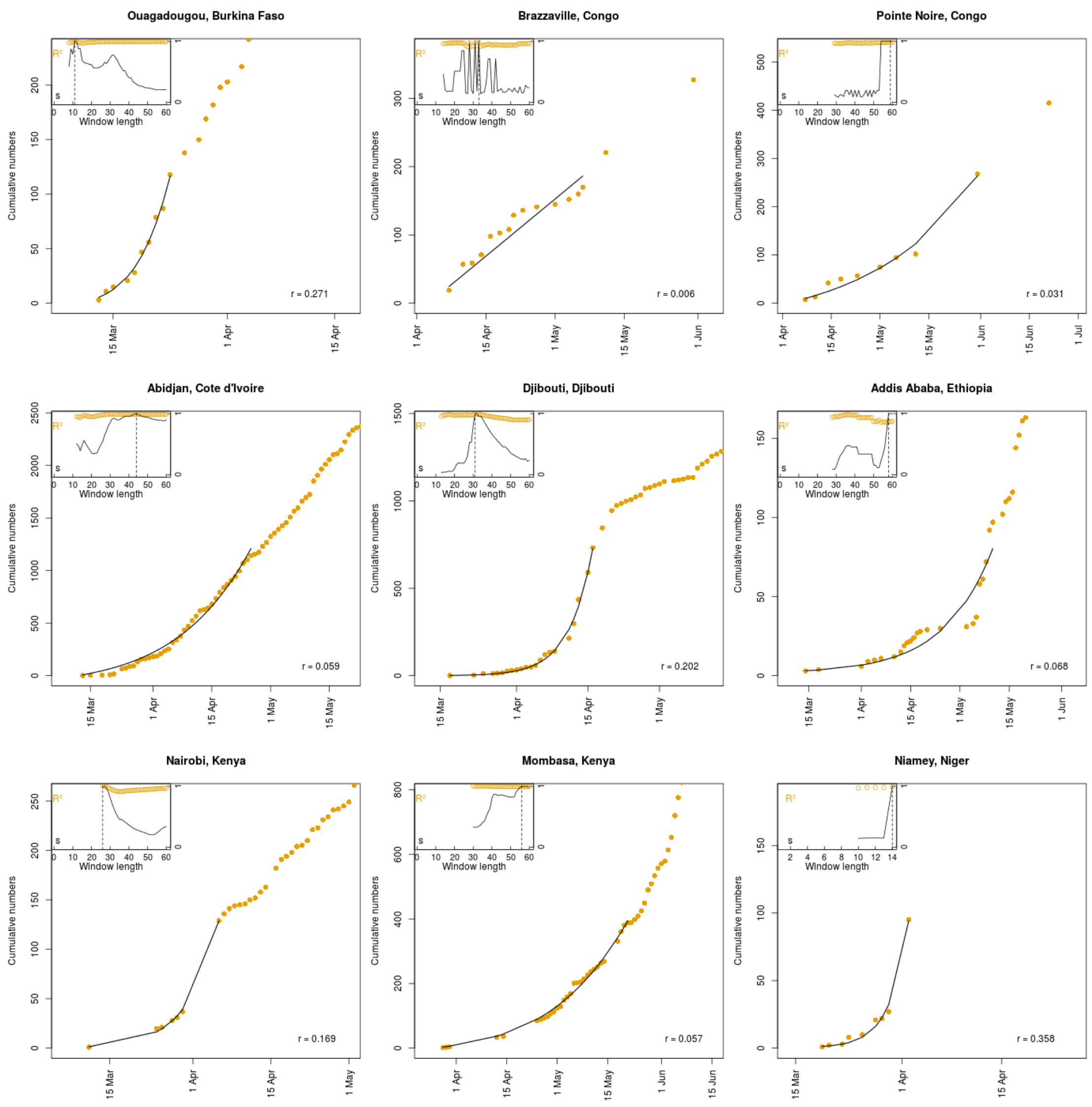

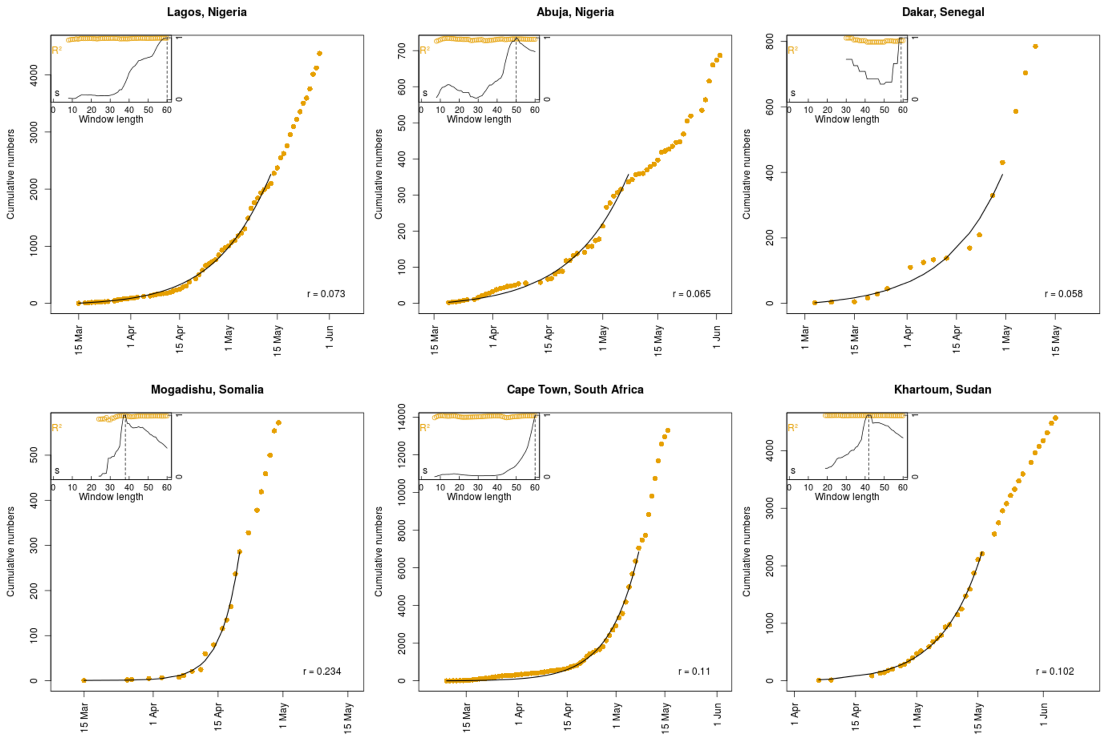
Lagos, Nigeria
Abuja, Nigeria
Dakar, Senegal
Mogadishu, Somalia
Cape Town, South Africa
Khartoum, Sudan
Cumulative numbers
Window length
R²
s
r = 0.073
r = 0.065
r = 0.058
r = 0.234
r = 0.11
r = 0.102

S4.2 America, North

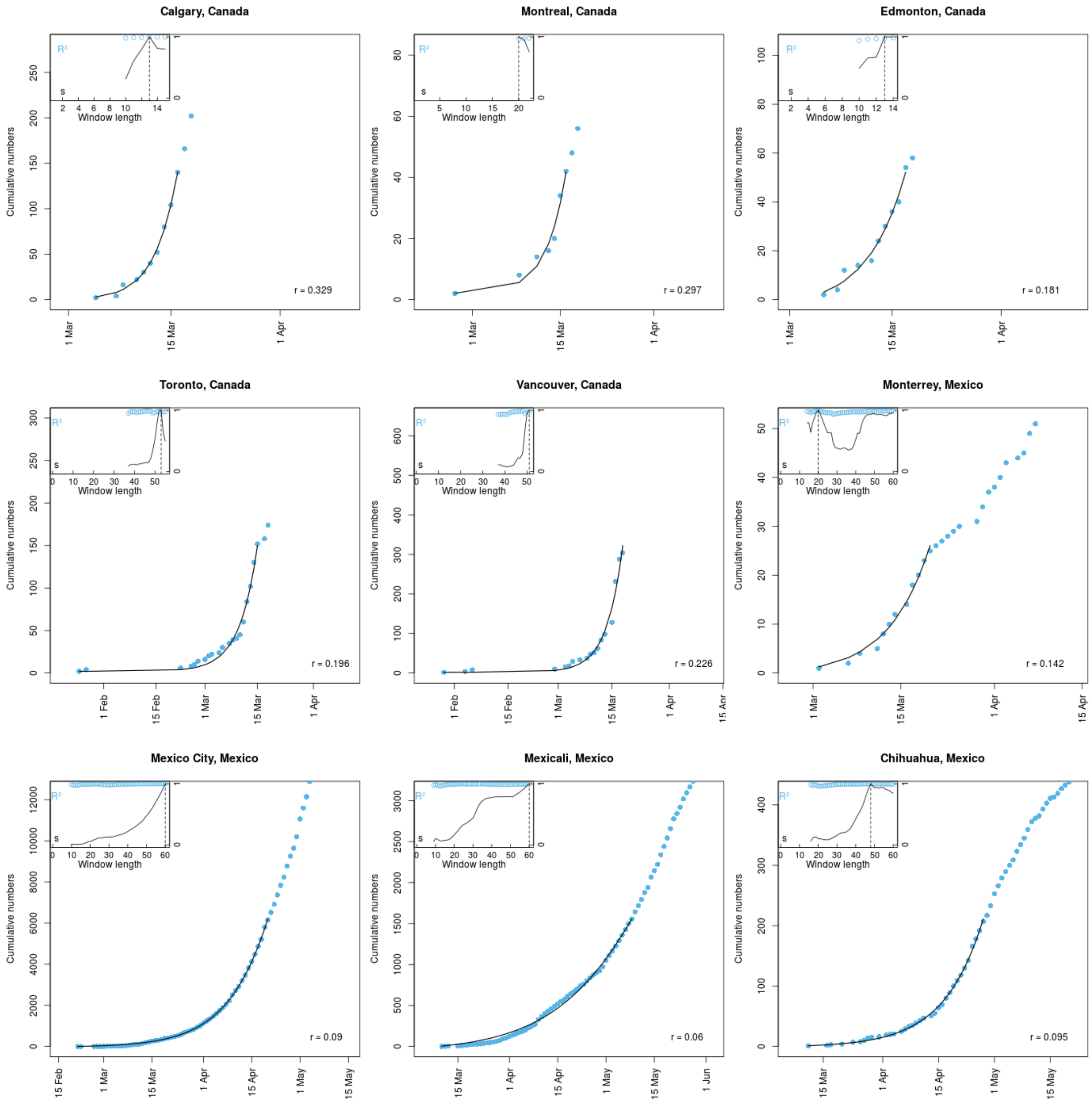

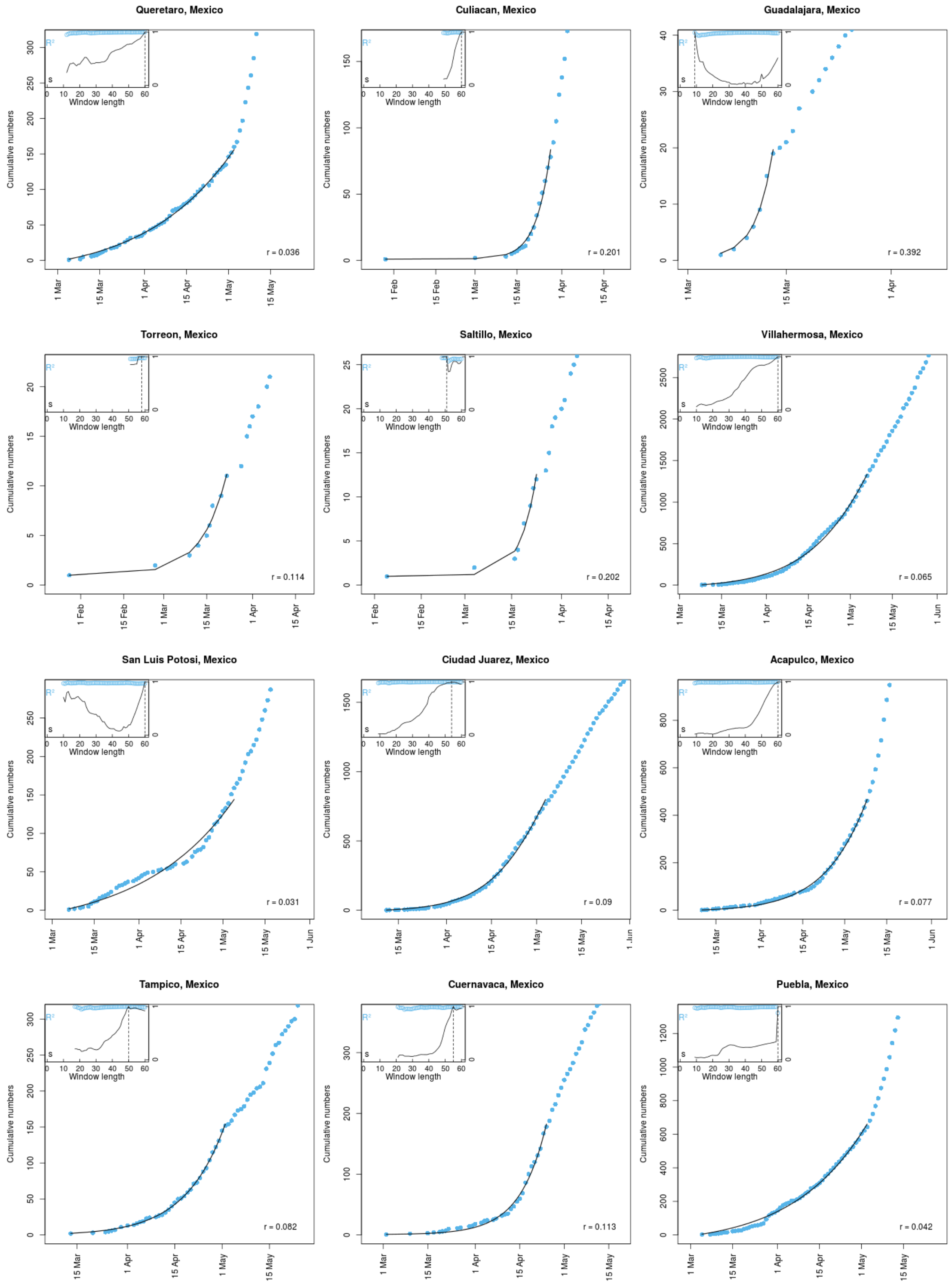
Queretaro, Mexico
r = 0.036
Culiacan, Mexico
r = 0.201
Guadalajara, Mexico
r = 0.392
Torreon, Mexico
r = 0.114
Saltillo, Mexico
r = 0.202
Villahermosa, Mexico
r = 0.065
San Luis Potosi, Mexico
r = 0.031
Ciudad Juarez, Mexico
r = 0.09
Acapulco, Mexico
r = 0.077
Tampico, Mexico
r = 0.082
Cuernavaca, Mexico
r = 0.113
Puebla, Mexico
r = 0.042
Cumulative numbers
Window length
R²
s

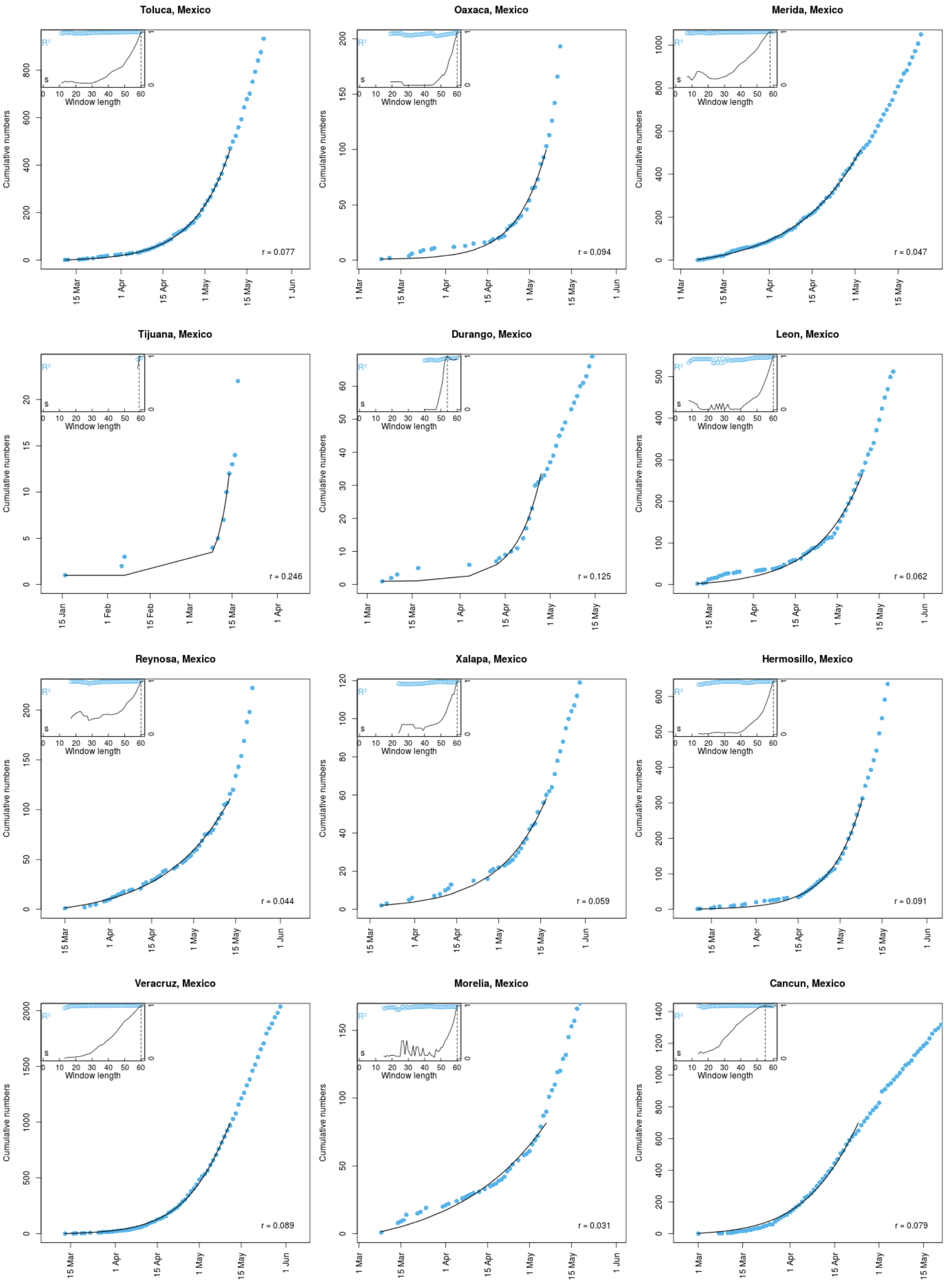
Toluca, Mexico
r = 0.077
Oaxaca, Mexico
r = 0.094
Merida, Mexico
r = 0.047
Tijuana, Mexico
r = 0.246
Durango, Mexico
r = 0.125
Leon, Mexico
r = 0.062
Reynosa, Mexico
r = 0.044
Xalapa, Mexico
r = 0.059
Hermosillo, Mexico
r = 0.091
Veracruz, Mexico
r = 0.089
Morelia, Mexico
r = 0.031
Cancun, Mexico
r = 0.079
Cumulative numbers
Window length

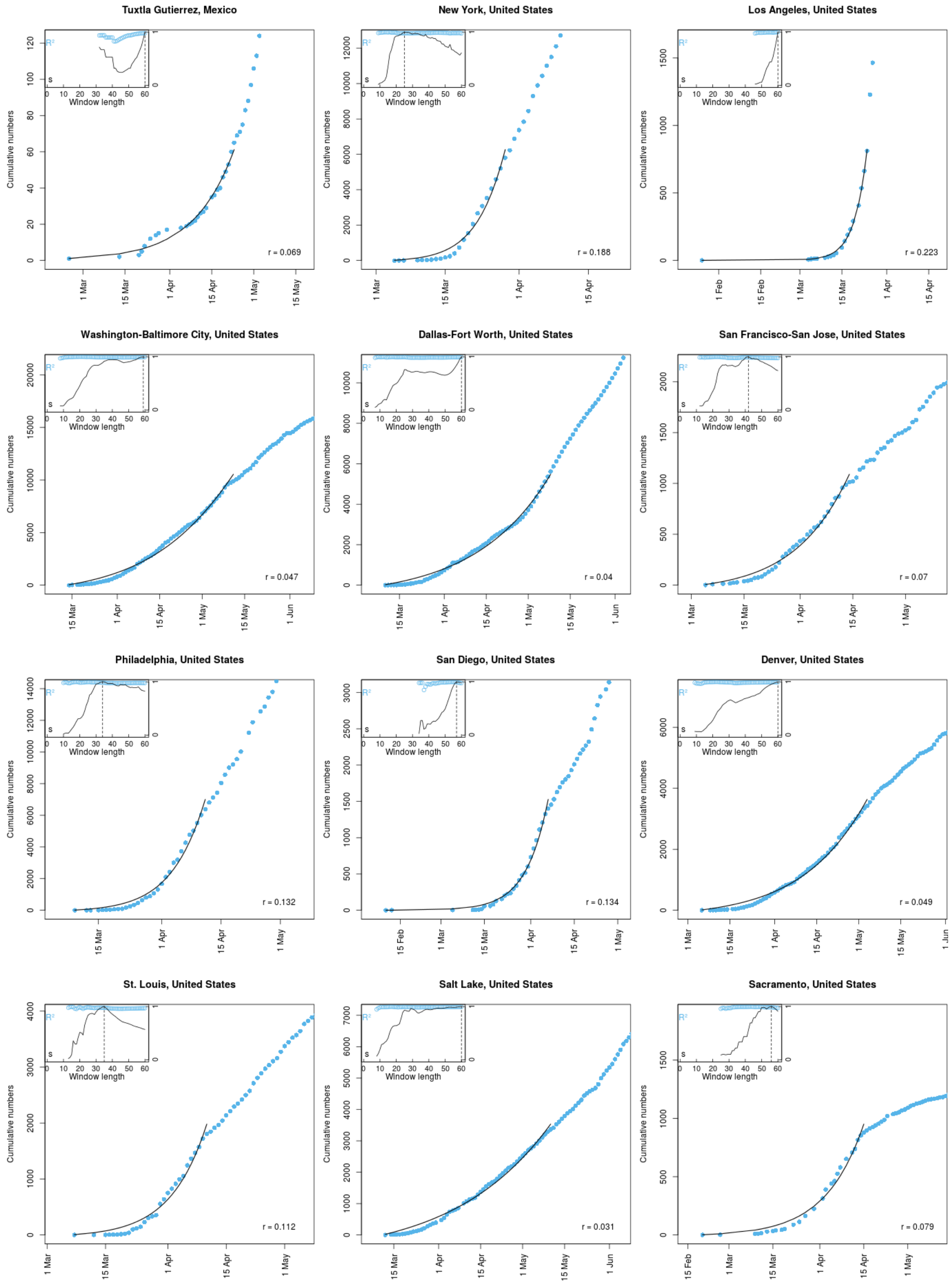
Tuxtla Gutierrez, Mexico
r = 0.069
New York, United States
r = 0.188
Los Angeles, United States
r = 0.223
Washington-Baltimore City, United States
r = 0.047
Dallas-Fort Worth, United States
r = 0.04
San Francisco-San Jose, United States
r = 0.07
Philadelphia, United States
r = 0.132
San Diego, United States
r = 0.134
Denver, United States
r = 0.049
St. Louis, United States
r = 0.112
Salt Lake, United States
r = 0.031
Sacramento, United States
r = 0.079
Cumulative numbers
Window length
R²
s

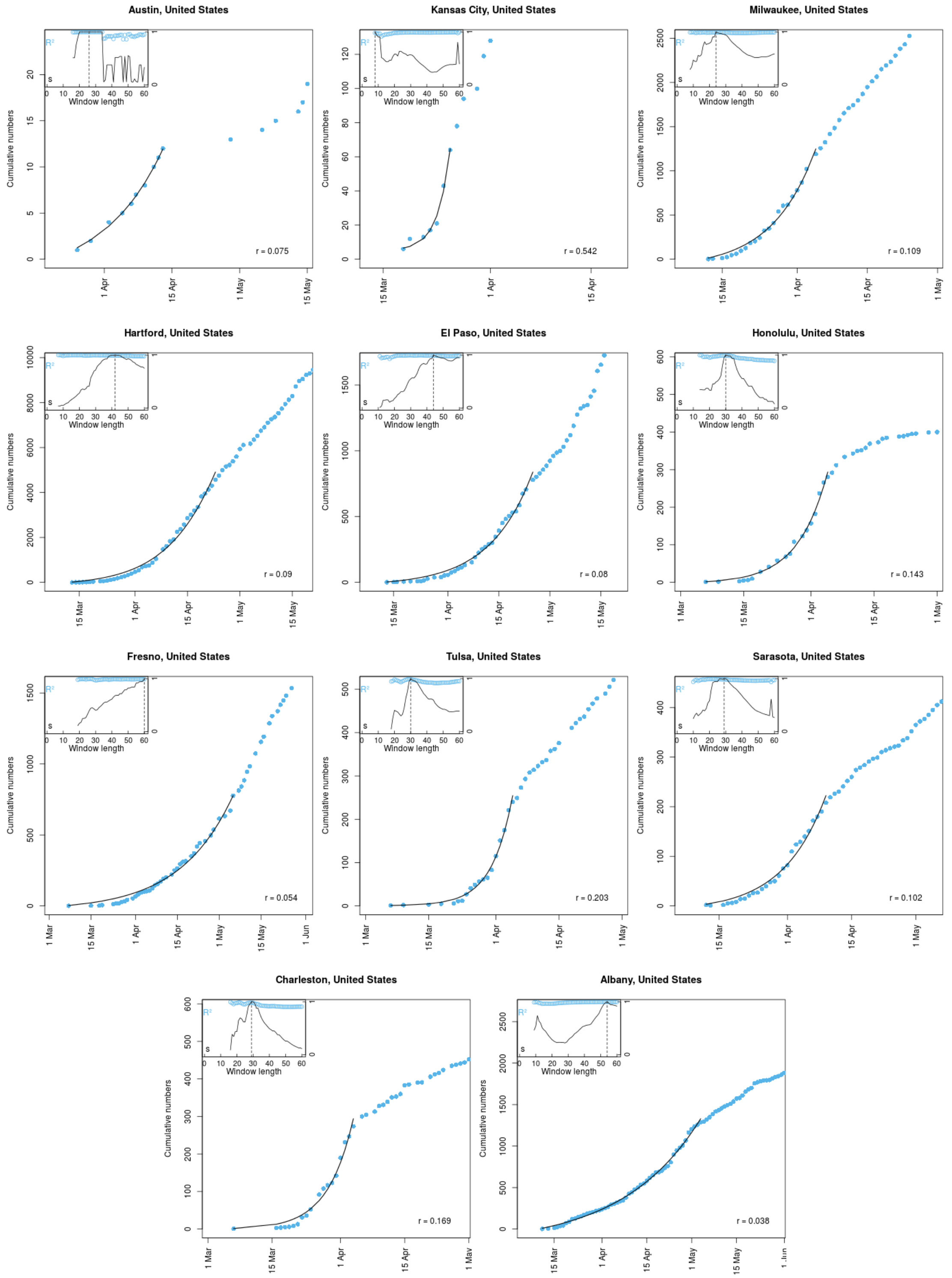
Austin, United States
r = 0.075
Kansas City, United States
r = 0.542
Milwaukee, United States
r = 0.109
Hartford, United States
r = 0.09
El Paso, United States
r = 0.08
Honolulu, United States
r = 0.143
Fresno, United States
r = 0.054
Tulsa, United States
r = 0.203
Sarasota, United States
r = 0.102
Charleston, United States
r = 0.169
Albany, United States
r = 0.038
Cumulative numbers
Window length
R²
s

S4.3 America, South

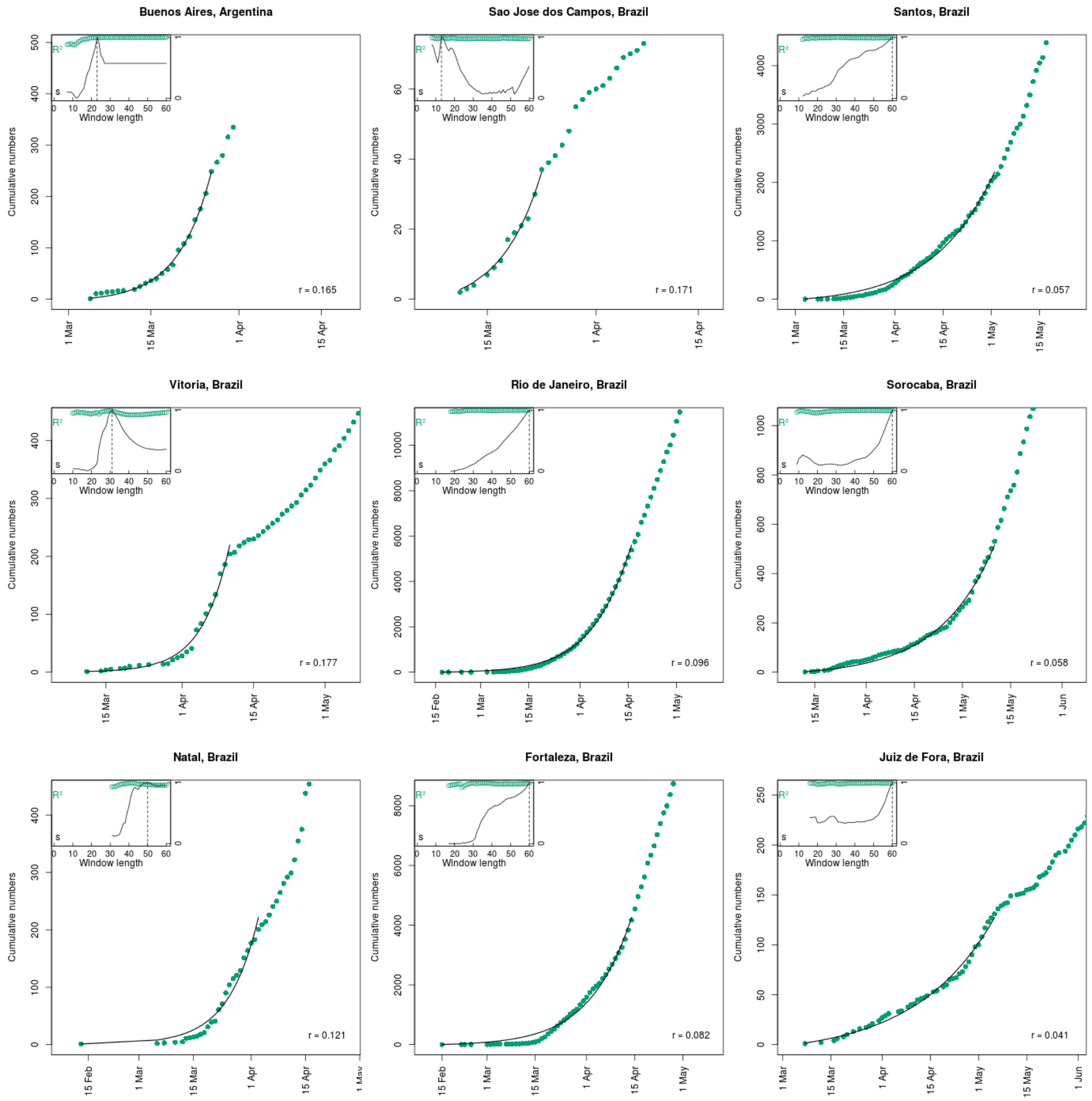

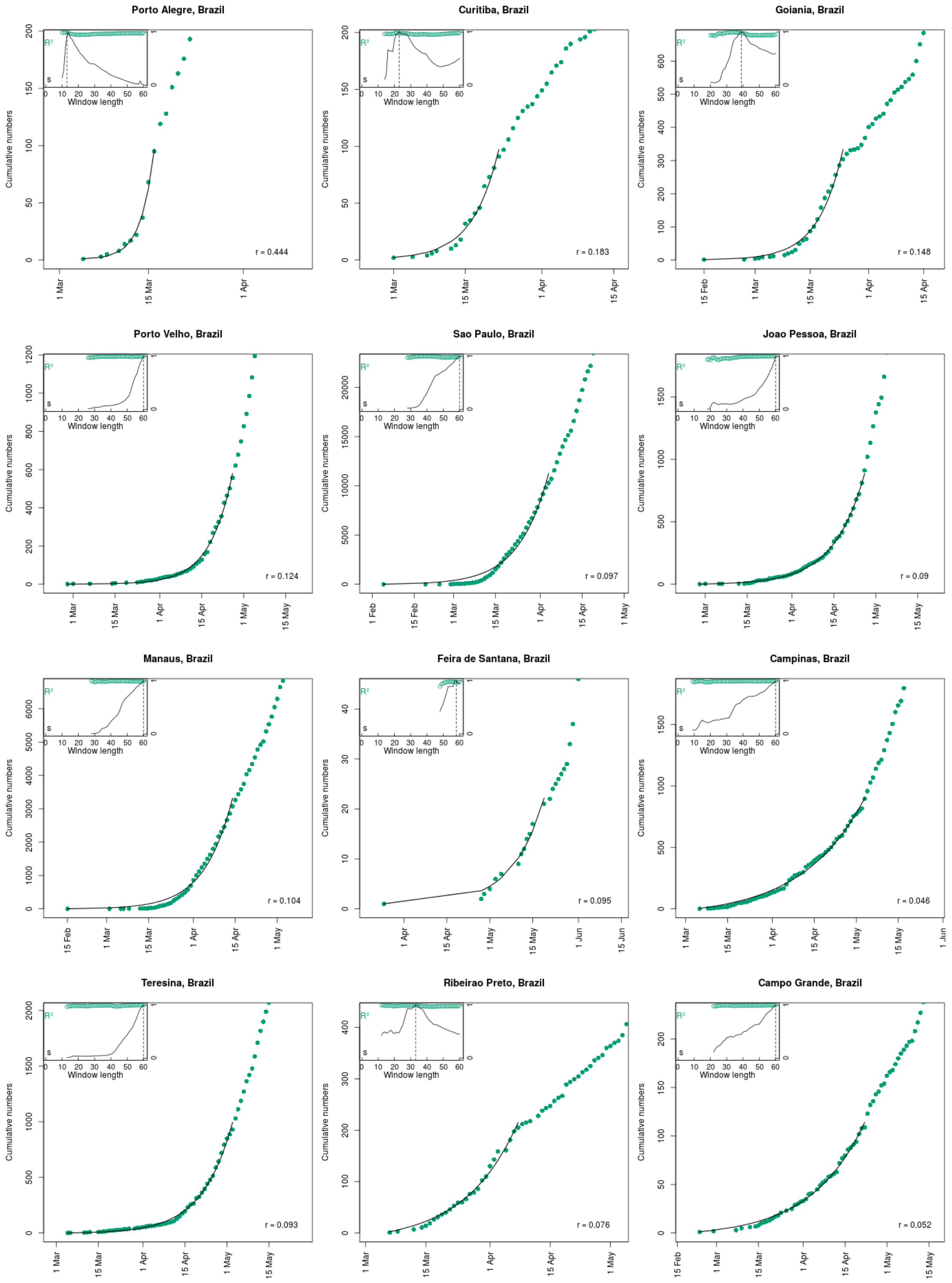
Porto Alegre, Brazil
r = 0.444
Curitiba, Brazil
r = 0.183
Goiania, Brazil
r = 0.148
Porto Velho, Brazil
r = 0.124
Sao Paulo, Brazil
r = 0.097
Joao Pessoa, Brazil
r = 0.09
Manaus, Brazil
r = 0.104
Feira de Santana, Brazil
r = 0.095
Campinas, Brazil
r = 0.046
Teresina, Brazil
r = 0.093
Ribeirao Preto, Brazil
r = 0.076
Campo Grande, Brazil
r = 0.052
Cumulative numbers
Window length
R²
S

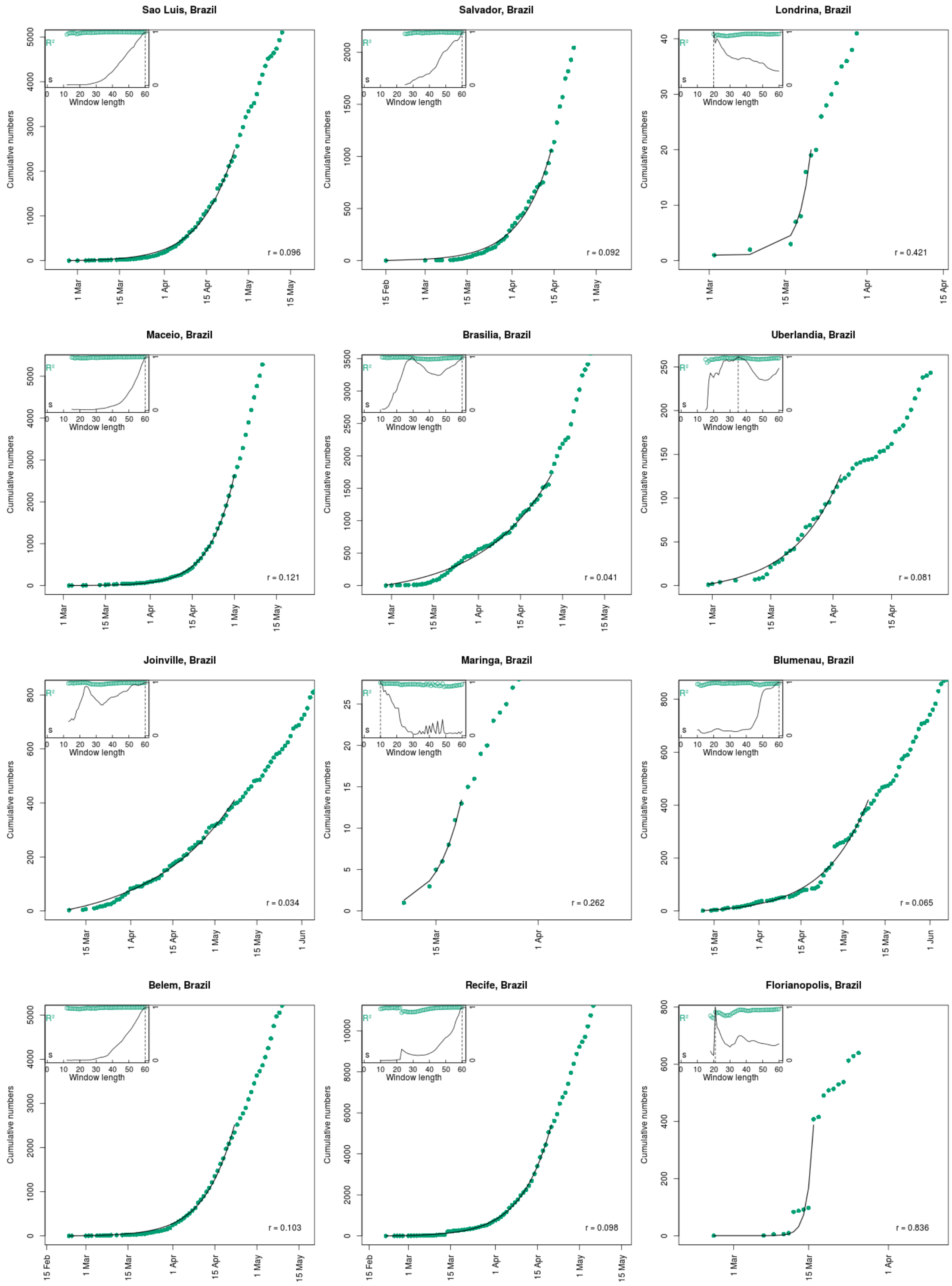
Sao Luis, Brazil
r = 0.096
Salvador, Brazil
r = 0.092
Londrina, Brazil
r = 0.421
Maceio, Brazil
r = 0.121
Brasilia, Brazil
r = 0.041
Uberlandia, Brazil
r = 0.081
Joinville, Brazil
r = 0.034
Maringa, Brazil
r = 0.262
Blumenau, Brazil
r = 0.065
Belem, Brazil
r = 0.103
Recife, Brazil
r = 0.098
Florianopolis, Brazil
r = 0.836
Cumulative numbers
Window length
R²
s

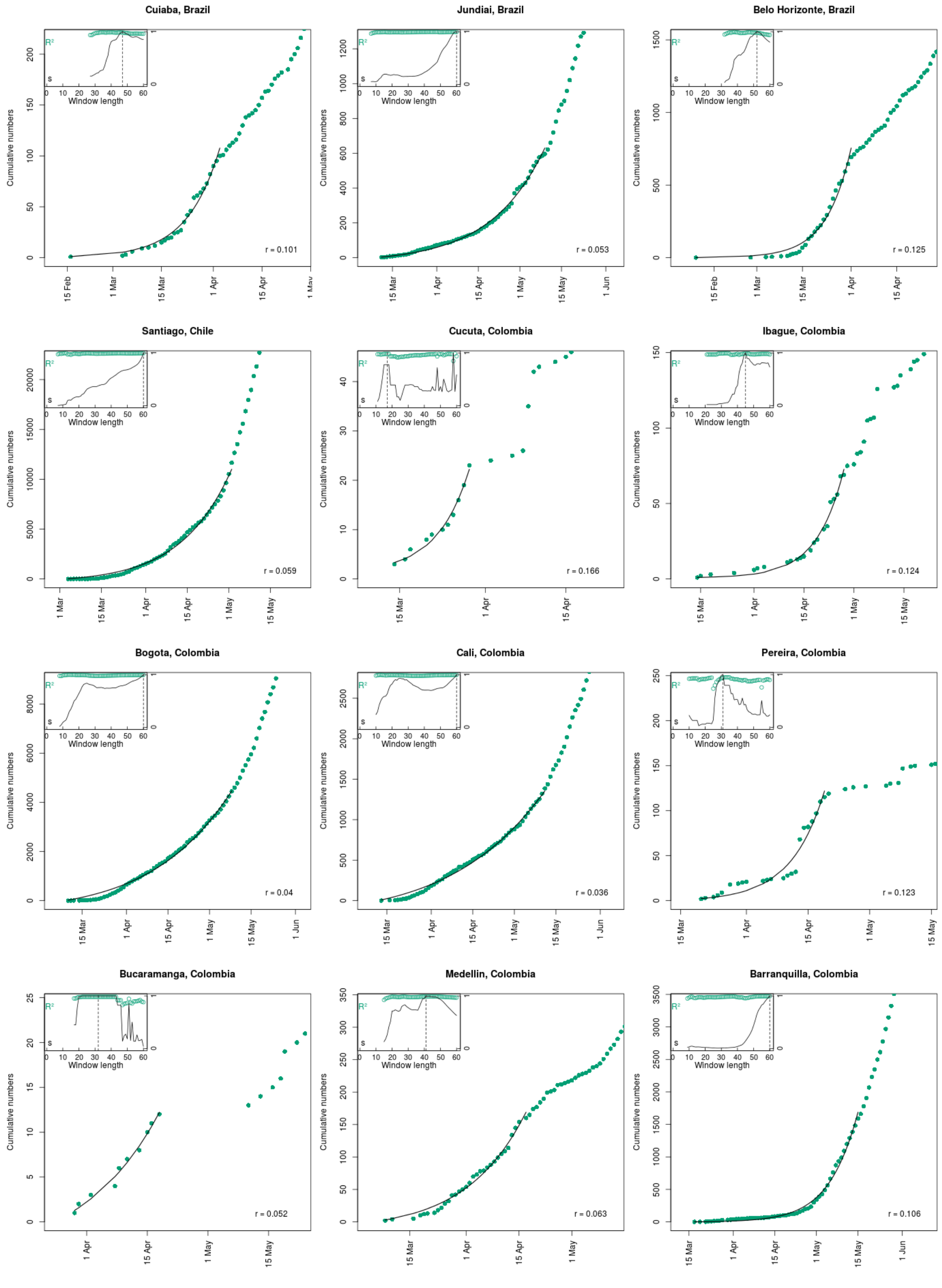
Cuiaba, Brazil
r = 0.101
Jundiai, Brazil
r = 0.053
Belo Horizonte, Brazil
r = 0.125
Santiago, Chile
r = 0.059
Cucuta, Colombia
r = 0.166
Ibague, Colombia
r = 0.124
Bogota, Colombia
r = 0.04
Cali, Colombia
r = 0.036
Pereira, Colombia
r = 0.123
Bucaramanga, Colombia
r = 0.052
Medellin, Colombia
r = 0.063
Barranquilla, Colombia
r = 0.106
Cumulative numbers
Window length
R²
s

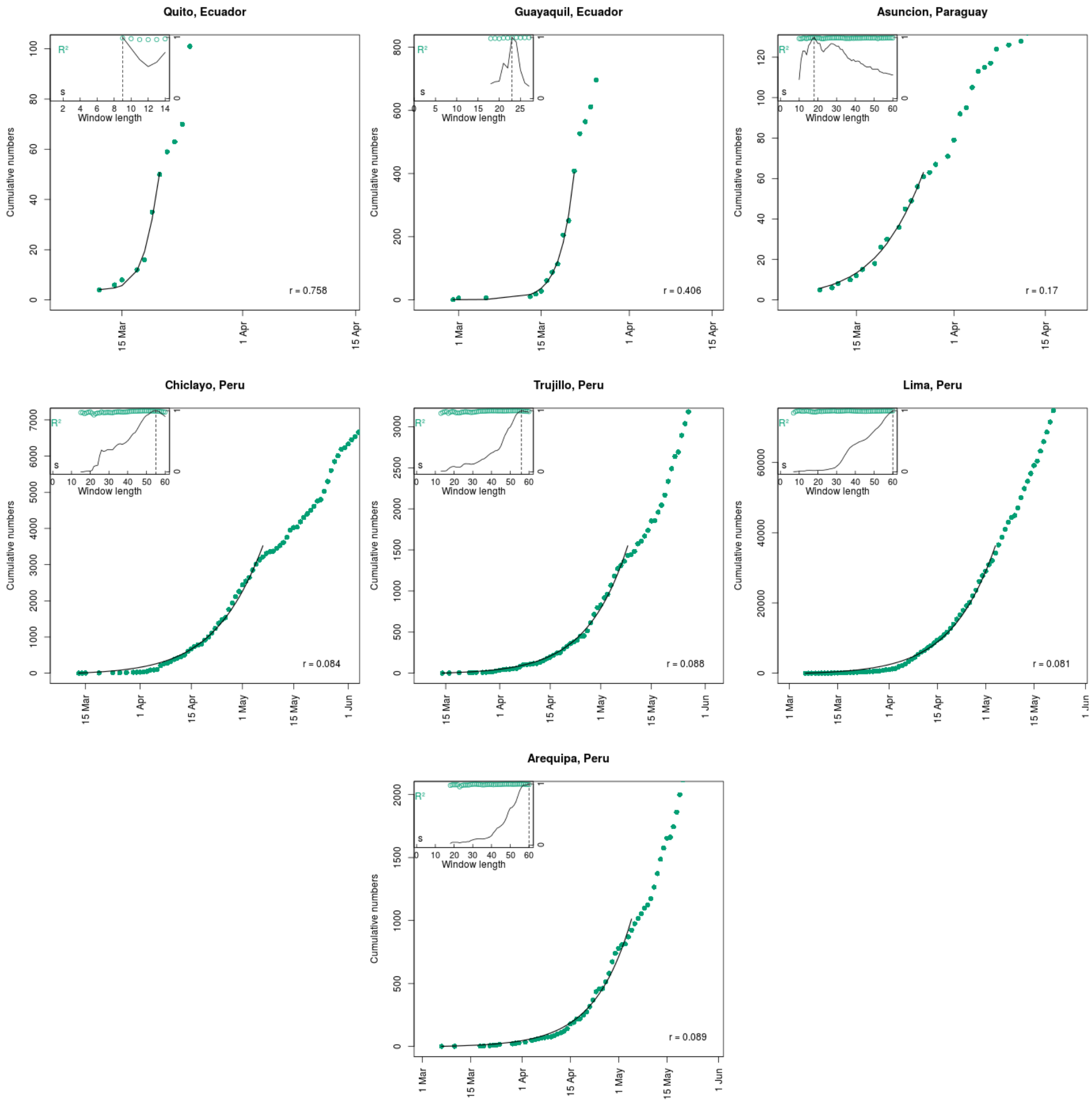
Quito, Ecuador
Guayaquil, Ecuador
Asuncion, Paraguay
Chiclayo, Peru
Trujillo, Peru
Lima, Peru
Arequipa, Peru
Cumulative numbers
Window length
R²
s
r = 0.758
r = 0.406
r = 0.17
r = 0.084
r = 0.088
r = 0.081
r = 0.089
1 Mar
15 Mar
1 Apr
15 Apr
1 May
15 May
1 Jun

S4.4 Australia

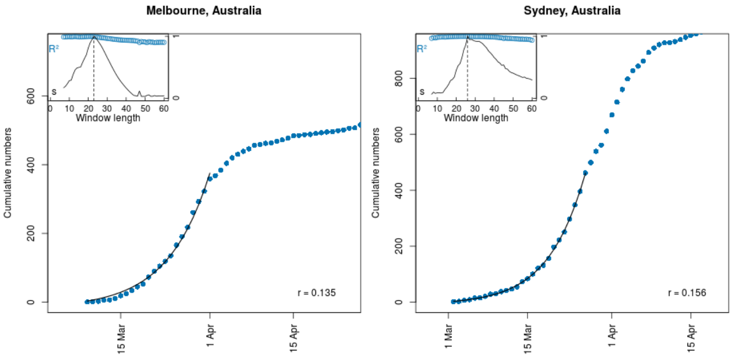

S4.5 Europe

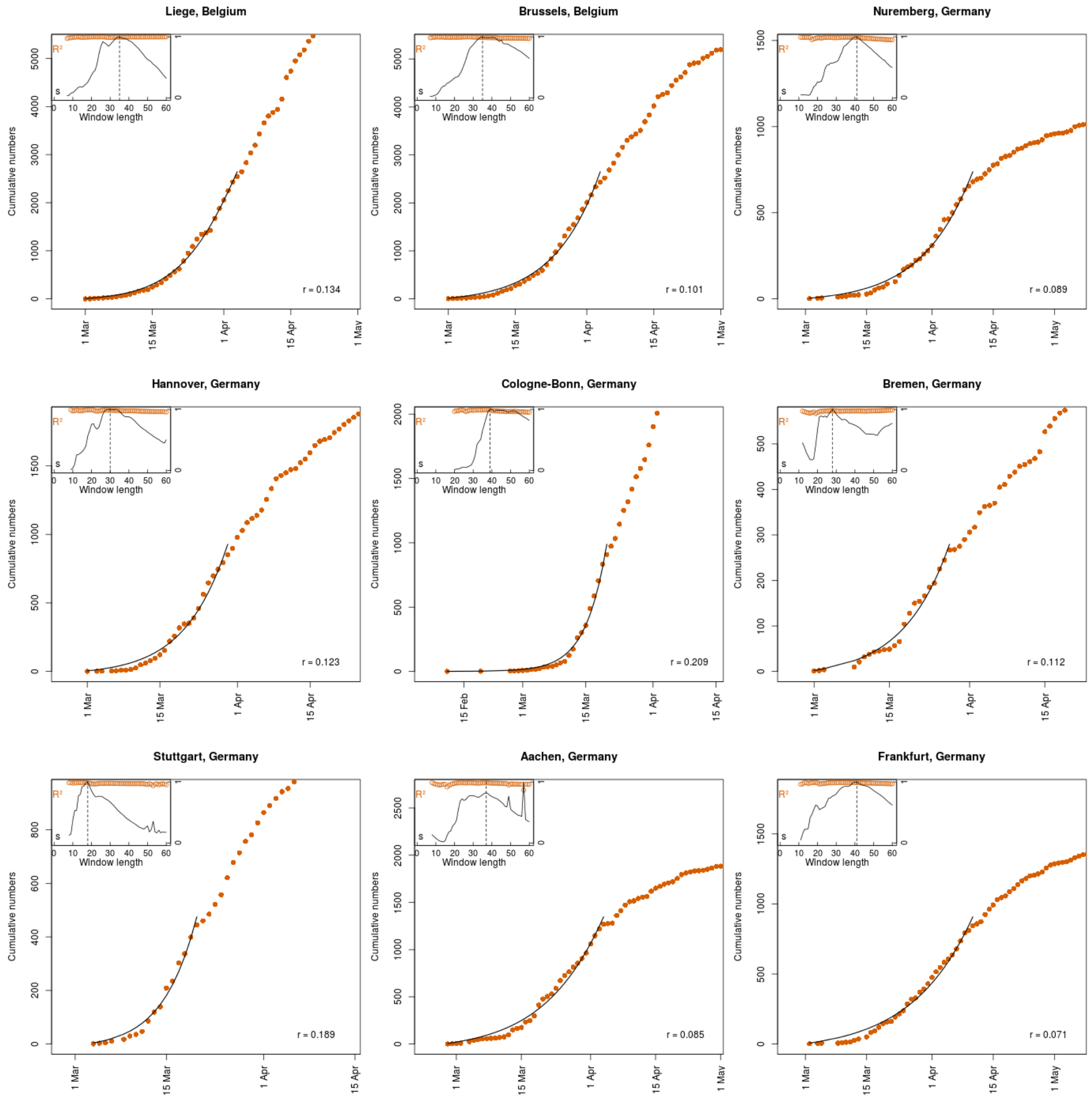
Liege, Belgium
r = 0.134
Brussels, Belgium
r = 0.101
Nuremberg, Germany
r = 0.089
Hannover, Germany
r = 0.123
Cologne-Bonn, Germany
r = 0.209
Bremen, Germany
r = 0.112
Stuttgart, Germany
r = 0.189
Aachen, Germany
r = 0.085
Frankfurt, Germany
r = 0.071
Cumulative numbers
Window length
R²
s
1 Mar
15 Mar
1 Apr
15 Apr
1 May
15 Feb

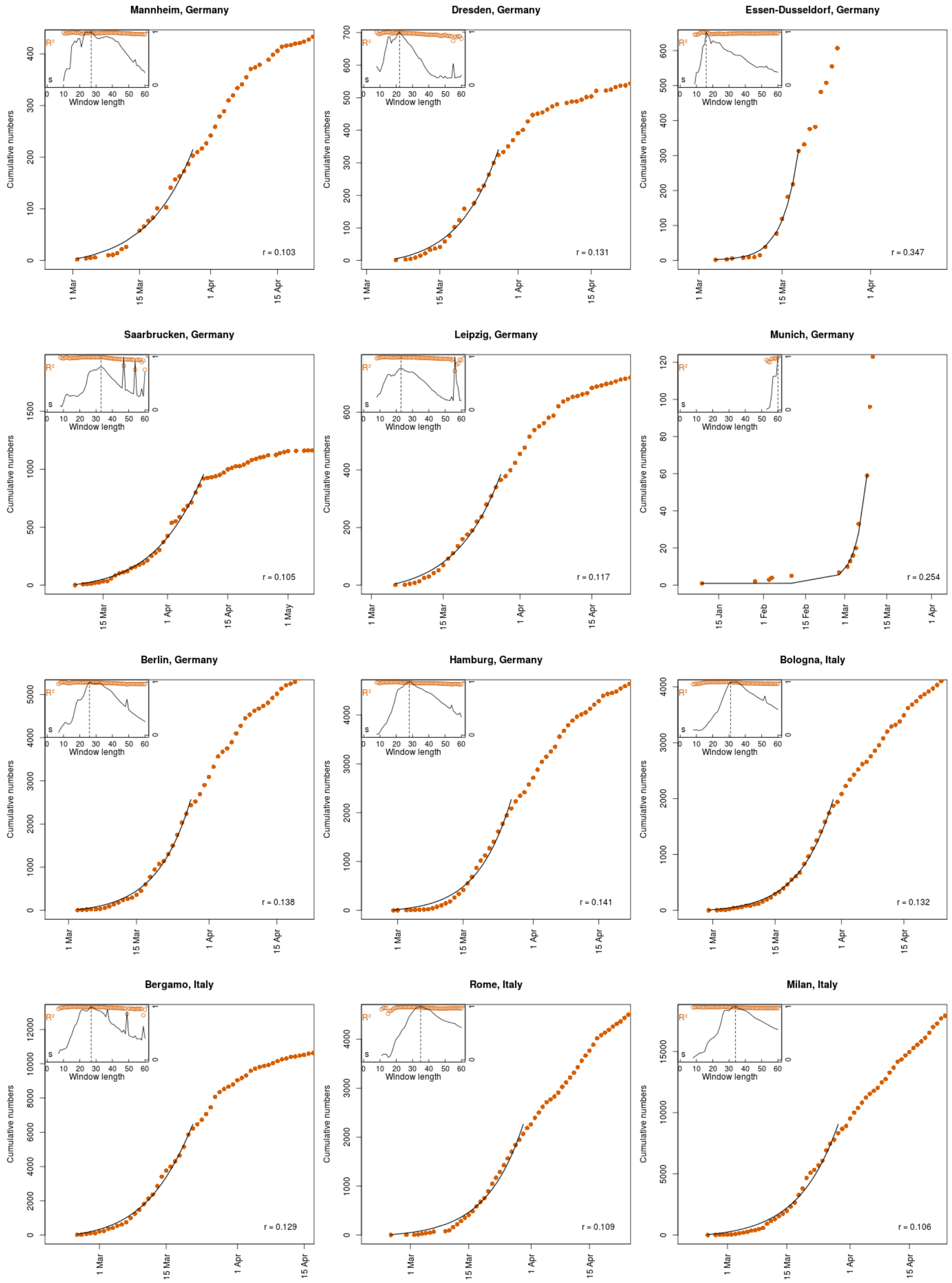
Mannheim, Germany
r = 0.103
Dresden, Germany
r = 0.131
Essen-Dusseldorf, Germany
r = 0.347
Saarbrucken, Germany
r = 0.105
Leipzig, Germany
r = 0.117
Munich, Germany
r = 0.254
Berlin, Germany
r = 0.138
Hamburg, Germany
r = 0.141
Bologna, Italy
r = 0.132
Bergamo, Italy
r = 0.129
Rome, Italy
r = 0.109
Milan, Italy
r = 0.106
Cumulative numbers
Window length
R²
S

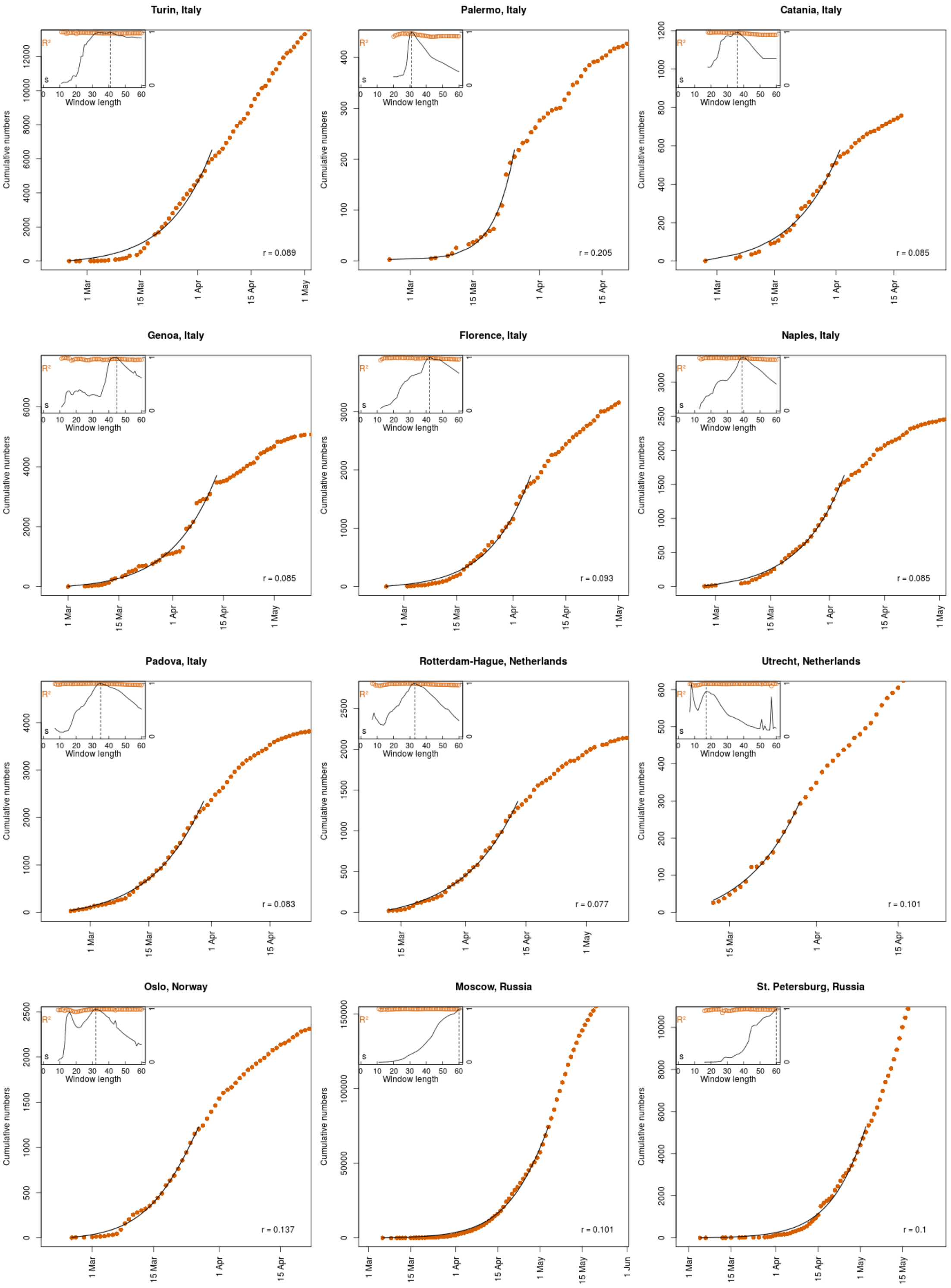
Turin, Italy
r = 0.089
Palermo, Italy
r = 0.205
Catania, Italy
r = 0.085
Genoa, Italy
r = 0.085
Florence, Italy
r = 0.093
Naples, Italy
r = 0.085
Padova, Italy
r = 0.083
Rotterdam-Hague, Netherlands
r = 0.077
Utrecht, Netherlands
r = 0.101
Oslo, Norway
r = 0.137
Moscow, Russia
r = 0.101
St. Petersburg, Russia
r = 0.1
Cumulative numbers
Window length
R²
s

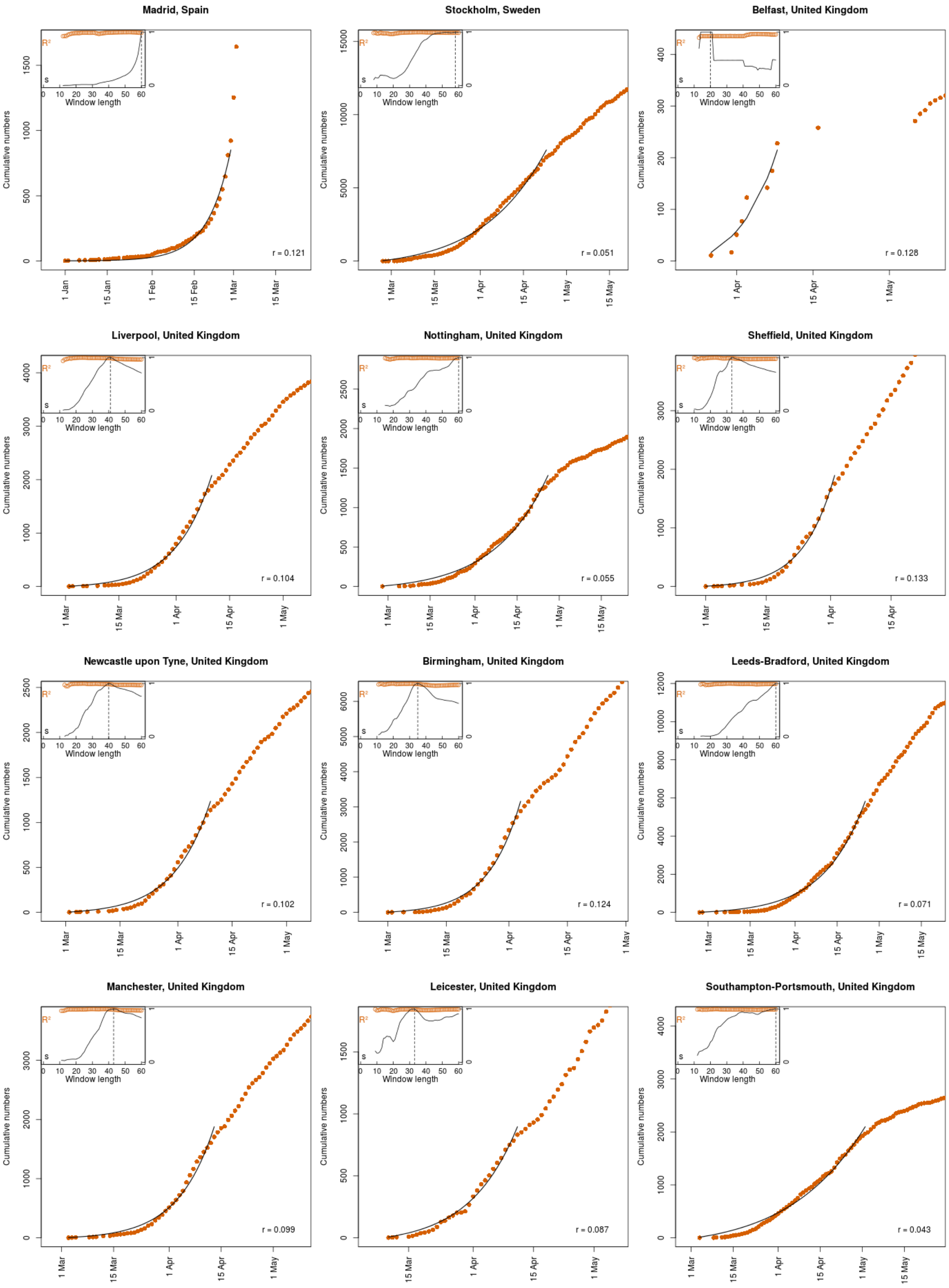
Madrid, Spain
Stockholm, Sweden
Belfast, United Kingdom
Liverpool, United Kingdom
Nottingham, United Kingdom
Sheffield, United Kingdom
Newcastle upon Tyne, United Kingdom
Birmingham, United Kingdom
Leeds-Bradford, United Kingdom
Manchester, United Kingdom
Leicester, United Kingdom
Southampton-Portsmouth, United Kingdom
Cumulative numbers
Window length
R²
s
r = 0.121
r = 0.051
r = 0.128
r = 0.104
r = 0.055
r = 0.133
r = 0.102
r = 0.124
r = 0.071
r = 0.099
r = 0.087
r = 0.043

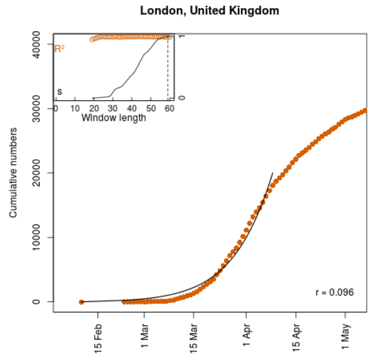

London, United Kingdom
Cumulative numbers
0
10000
20000
30000
40000
15 Feb
1 Mar
15 Mar
1 Apr
15 Apr
1 May
r = 0.096
R²
s
Window length
0
10
20
30
40
50
60

S4.6 Asia w/o China

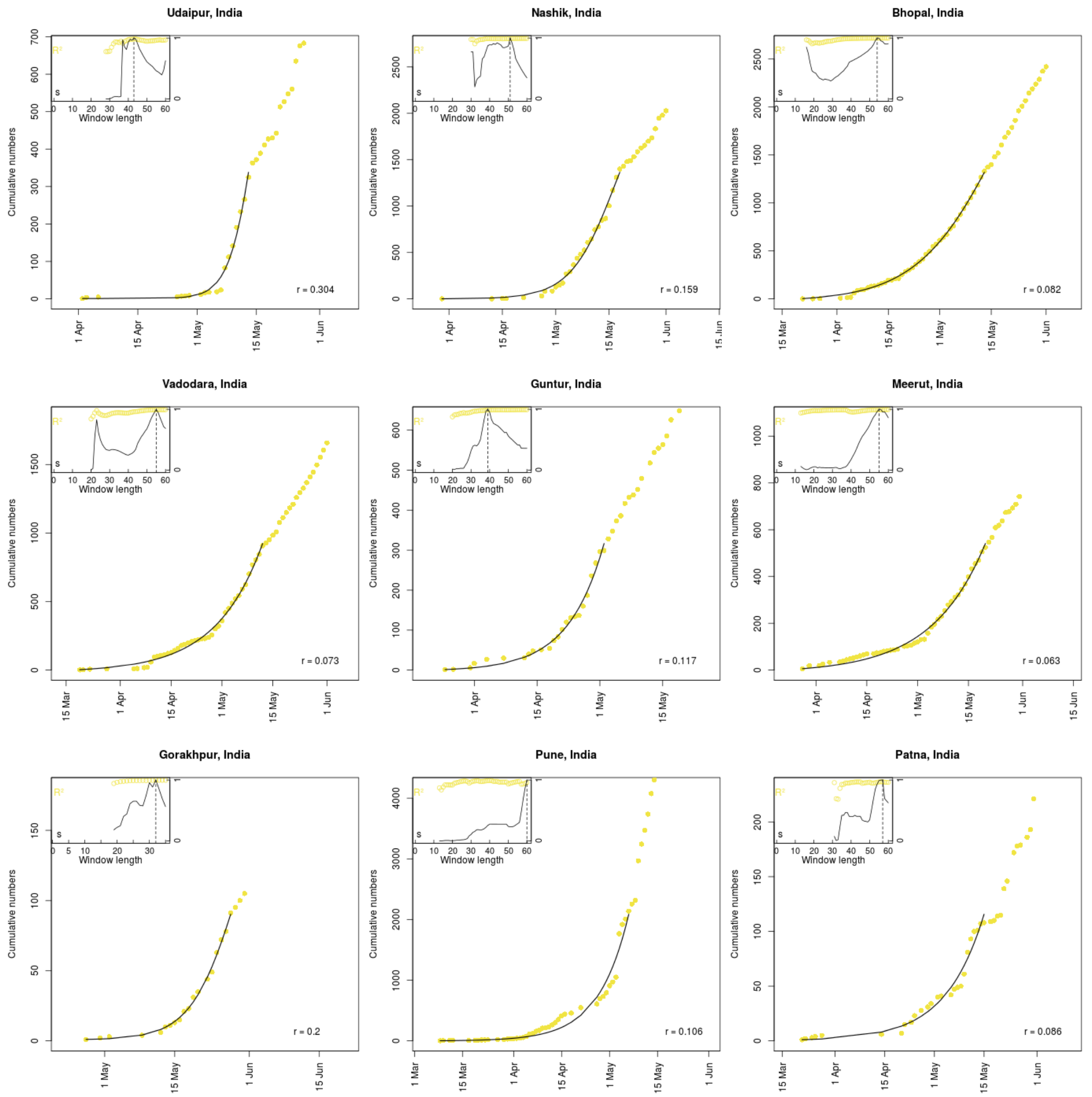

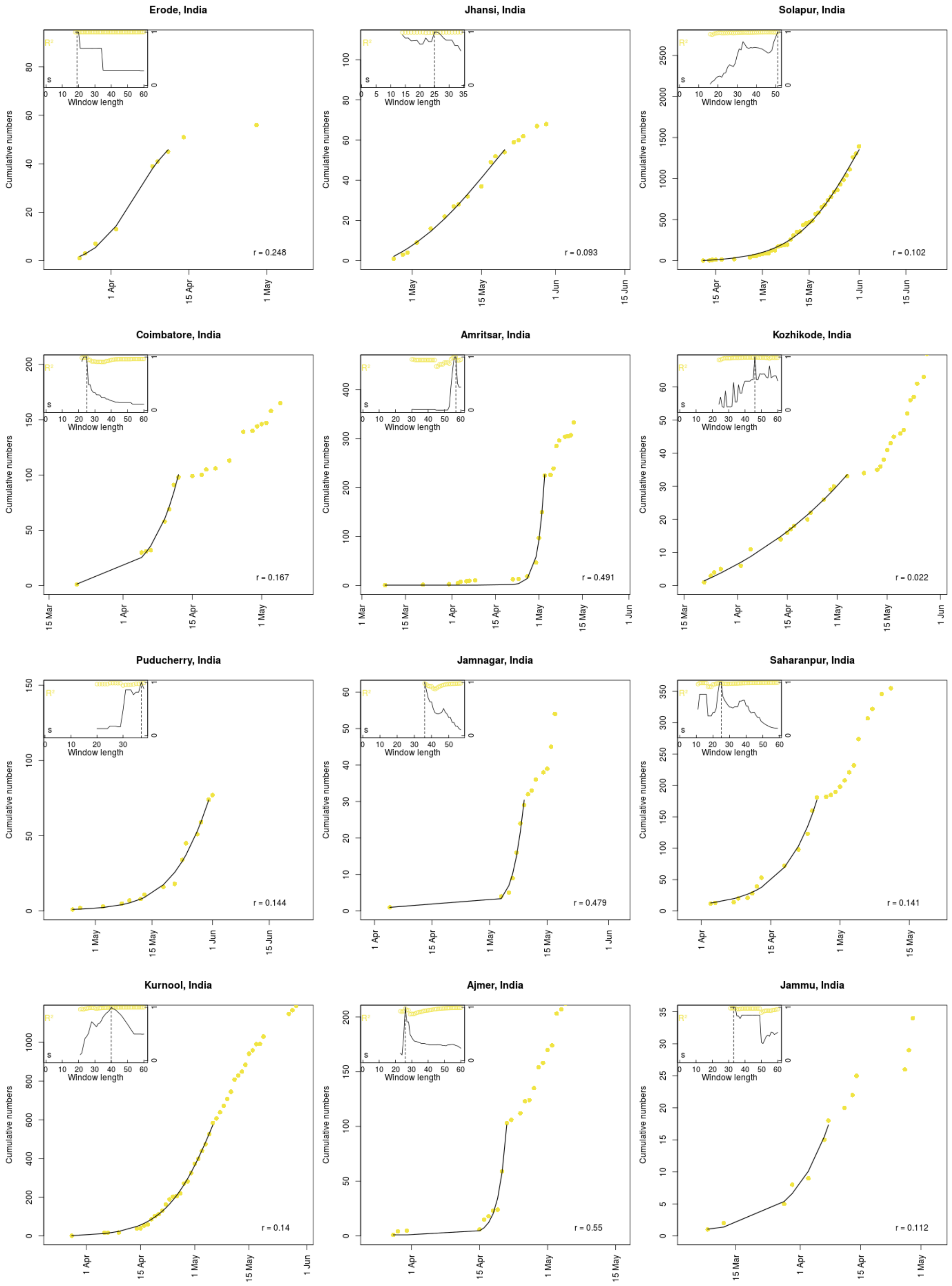
Erode, India
r = 0.248
Jhansi, India
r = 0.093
Solapur, India
r = 0.102
Coimbatore, India
r = 0.167
Amritsar, India
r = 0.491
Kozhikode, India
r = 0.022
Puducherry, India
r = 0.144
Jamnagar, India
r = 0.479
Saharanpur, India
r = 0.141
Kurnool, India
r = 0.14
Ajmer, India
r = 0.55
Jammu, India
r = 0.112
Cumulative numbers
Window length

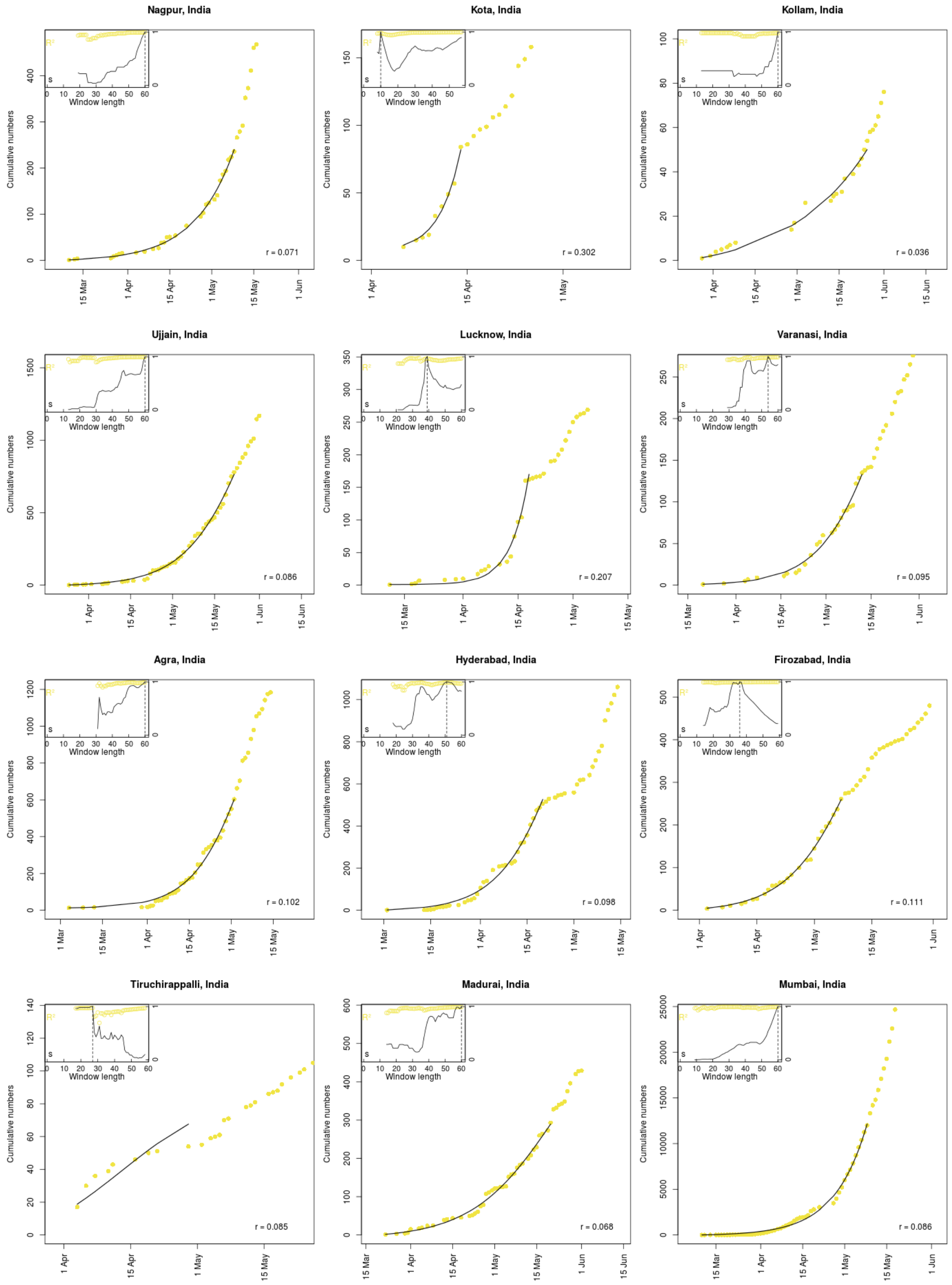
Nagpur, India
r = 0.071
Kota, India
r = 0.302
Kollam, India
r = 0.036
Ujjain, India
r = 0.086
Lucknow, India
r = 0.207
Varanasi, India
r = 0.095
Agra, India
r = 0.102
Hyderabad, India
r = 0.098
Firozabad, India
r = 0.111
Tiruchirappalli, India
r = 0.085
Madurai, India
r = 0.068
Mumbai, India
r = 0.086
Cumulative numbers
Window length

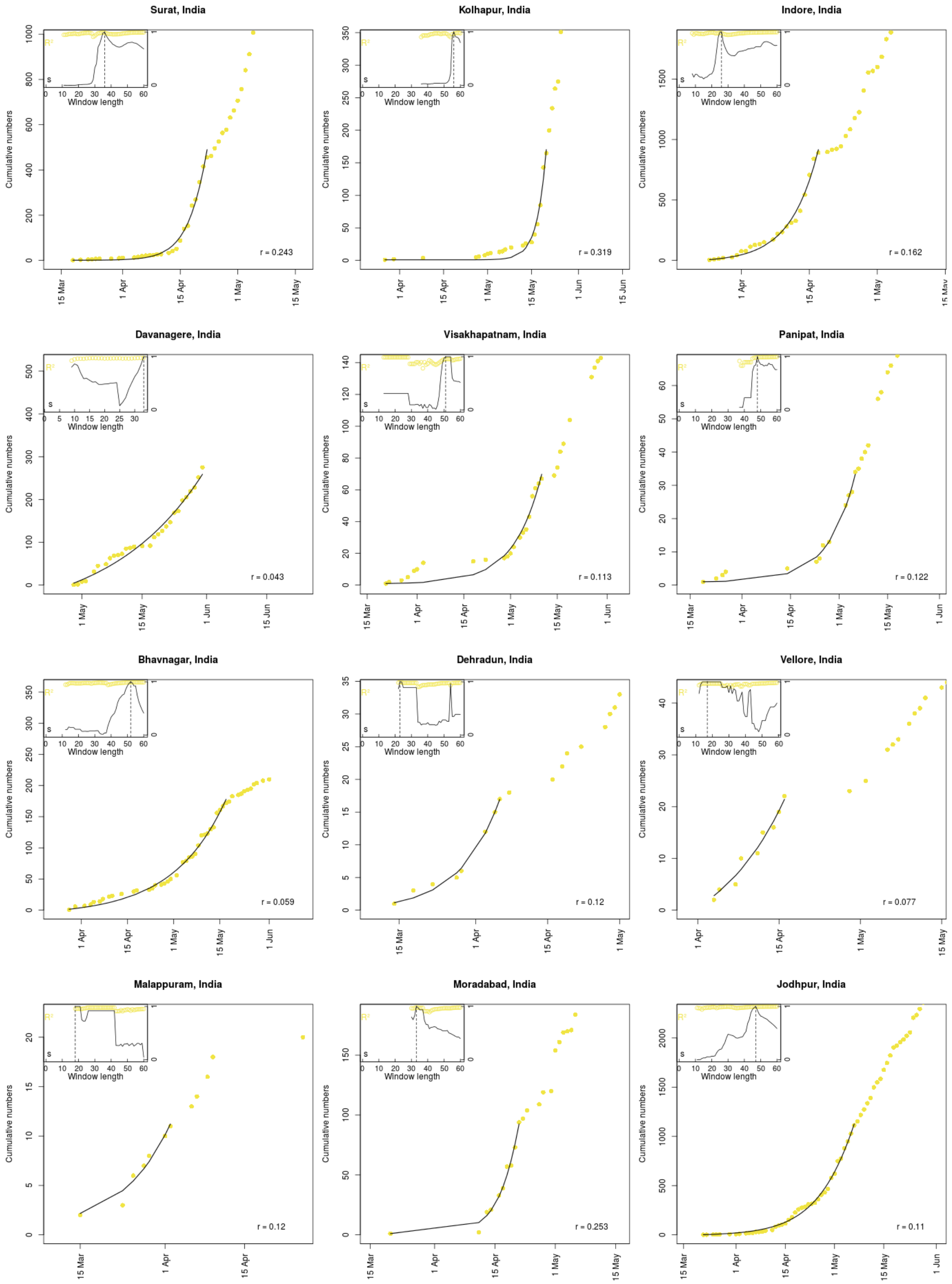
Surat, India
Cumulative numbers
Window length
r = 0.243
Kolhapur, India
r = 0.319
Indore, India
r = 0.162
Davanagere, India
r = 0.043
Visakhapatnam, India
r = 0.113
Panipat, India
r = 0.122
Bhavnagar, India
r = 0.059
Dehradun, India
r = 0.12
Vellore, India
r = 0.077
Malappuram, India
r = 0.12
Moradabad, India
r = 0.253
Jodhpur, India
r = 0.11

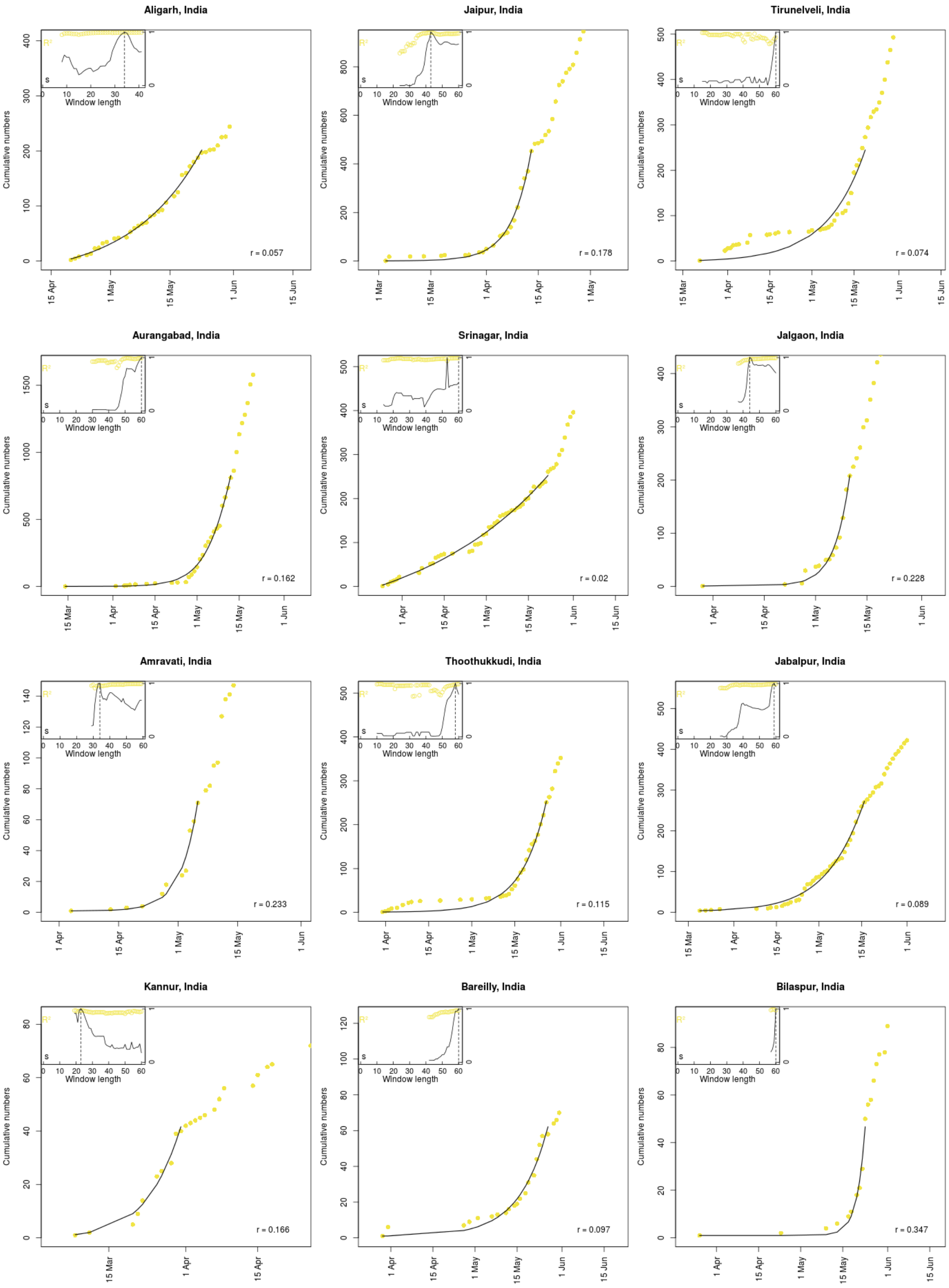
Aligarh, India
Cumulative numbers
Window length
r = 0.057
Jaipur, India
Cumulative numbers
Window length
r = 0.178
Tirunelveli, India
Cumulative numbers
Window length
r = 0.074
Aurangabad, India
Cumulative numbers
Window length
r = 0.162
Srinagar, India
Cumulative numbers
Window length
r = 0.02
Jalgaon, India
Cumulative numbers
Window length
r = 0.228
Amravati, India
Cumulative numbers
Window length
r = 0.233
Thoothukkudi, India
Cumulative numbers
Window length
r = 0.115
Jabalpur, India
Cumulative numbers
Window length
r = 0.089
Kannur, India
Cumulative numbers
Window length
r = 0.166
Bareilly, India
Cumulative numbers
Window length
r = 0.097
Bilaspur, India
Cumulative numbers
Window length
r = 0.347

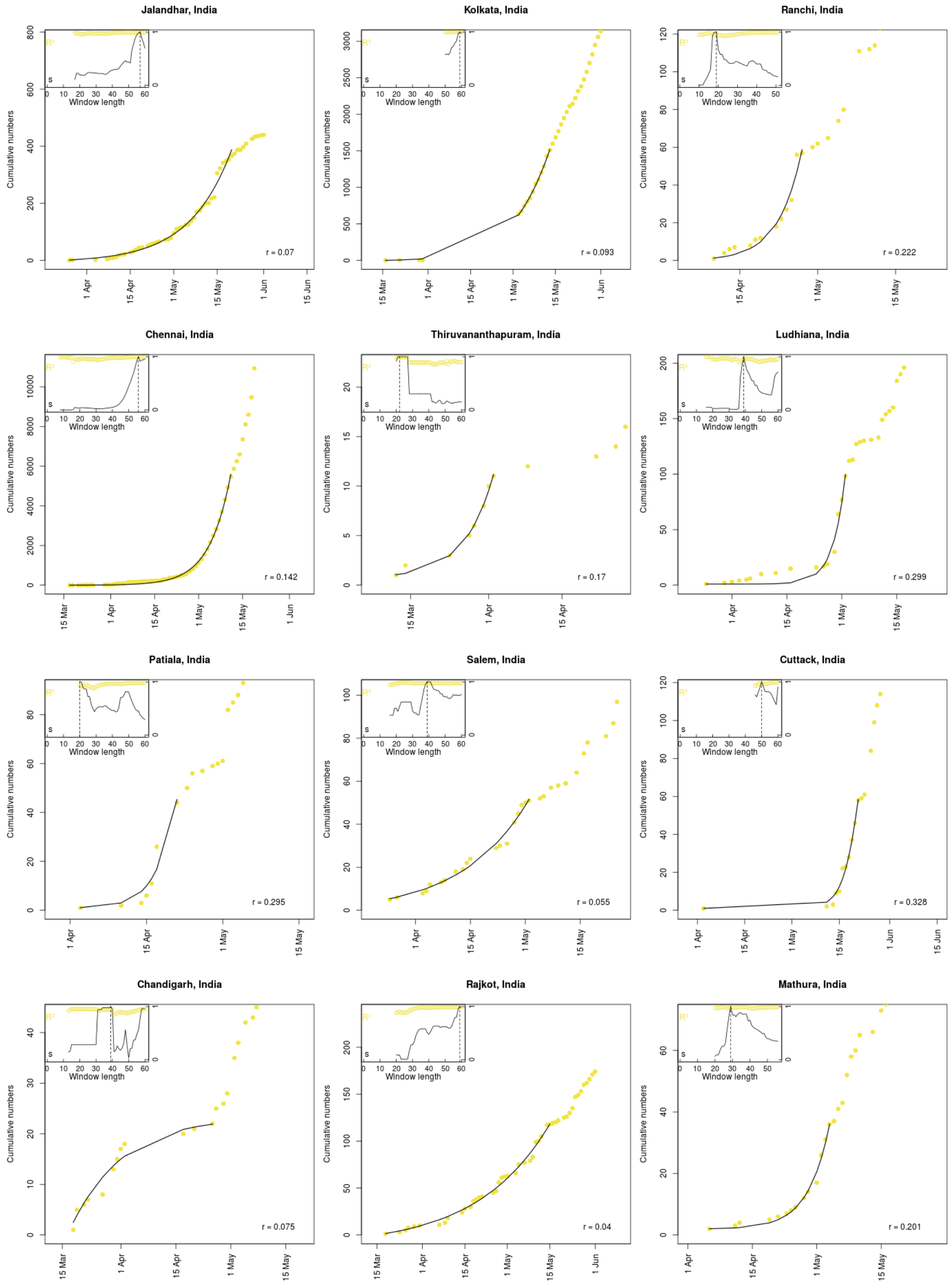
Jalandhar, India
r = 0.07
Kolkata, India
r = 0.093
Ranchi, India
r = 0.222
Chennai, India
r = 0.142
Thiruvananthapuram, India
r = 0.17
Ludhiana, India
r = 0.299
Patiala, India
r = 0.295
Salem, India
r = 0.055
Cuttack, India
r = 0.328
Chandigarh, India
r = 0.075
Rajkot, India
r = 0.04
Mathura, India
r = 0.201
Cumulative numbers
Window length
R²
s

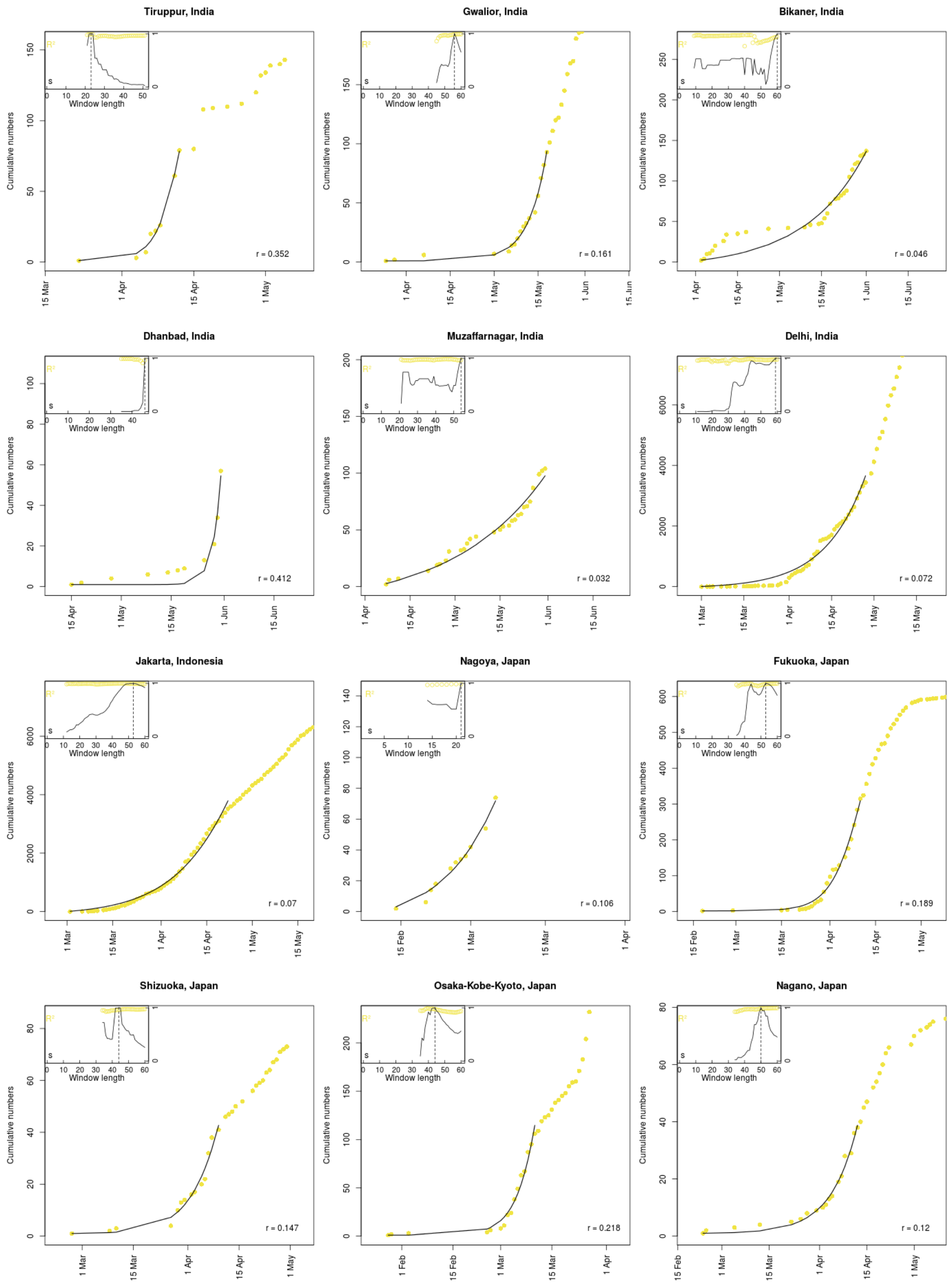
Tiruppur, India
r = 0.352
Gwalior, India
r = 0.161
Bikaner, India
r = 0.046
Dhanbad, India
r = 0.412
Muzaffarnagar, India
r = 0.032
Delhi, India
r = 0.072
Jakarta, Indonesia
r = 0.07
Nagoya, Japan
r = 0.106
Fukuoka, Japan
r = 0.189
Shizuoka, Japan
r = 0.147
Osaka-Kobe-Kyoto, Japan
r = 0.218
Nagano, Japan
r = 0.12
Cumulative numbers
Window length

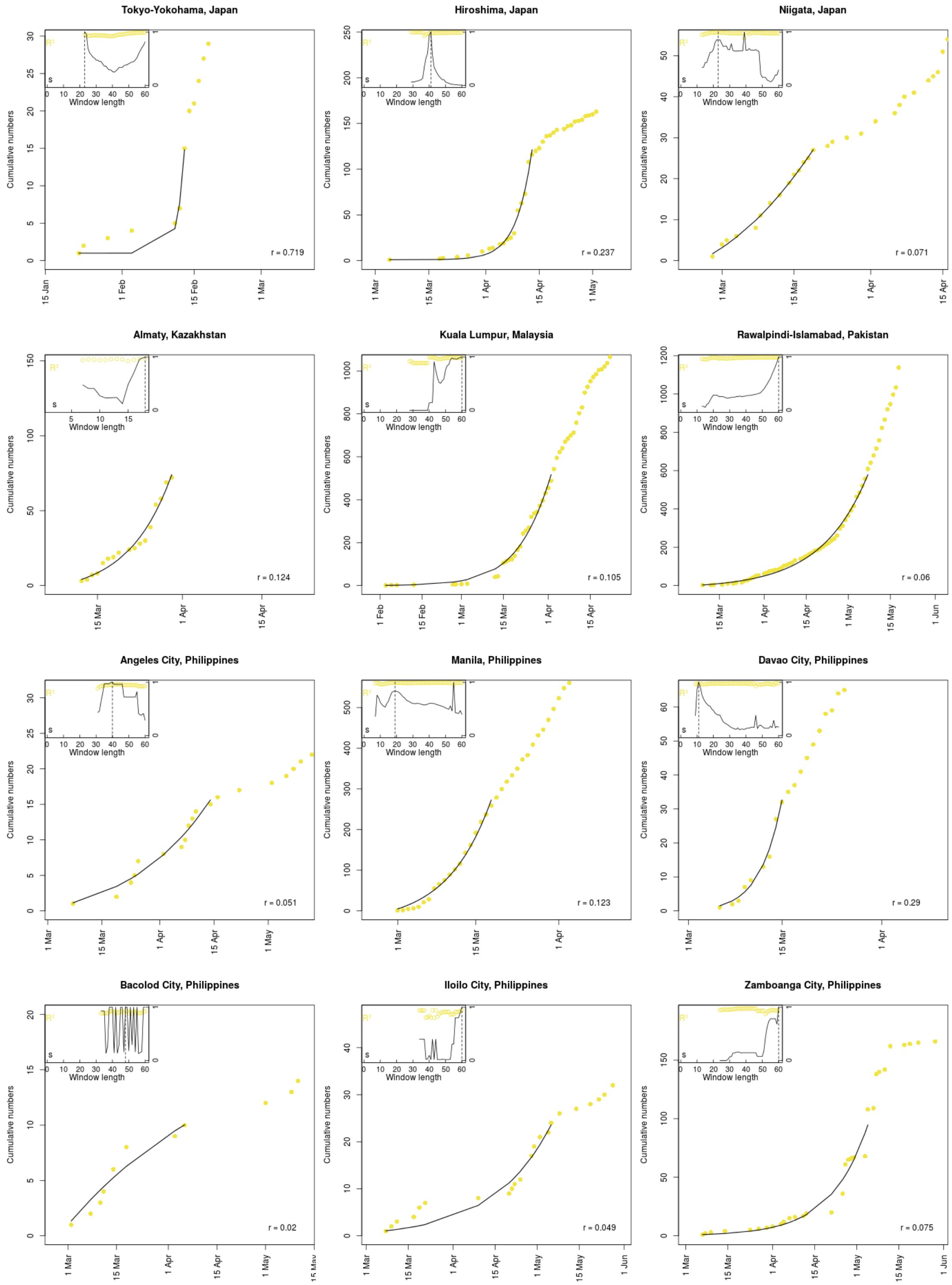
Tokyo-Yokohama, Japan
r = 0.719
Hiroshima, Japan
r = 0.237
Niigata, Japan
r = 0.071
Almaty, Kazakhstan
r = 0.124
Kuala Lumpur, Malaysia
r = 0.105
Rawalpindi-Islamabad, Pakistan
r = 0.06
Angeles City, Philippines
r = 0.051
Manila, Philippines
r = 0.123
Davao City, Philippines
r = 0.29
Bacolod City, Philippines
r = 0.02
Iloilo City, Philippines
r = 0.049
Zamboanga City, Philippines
r = 0.075
Cumulative numbers
Window length

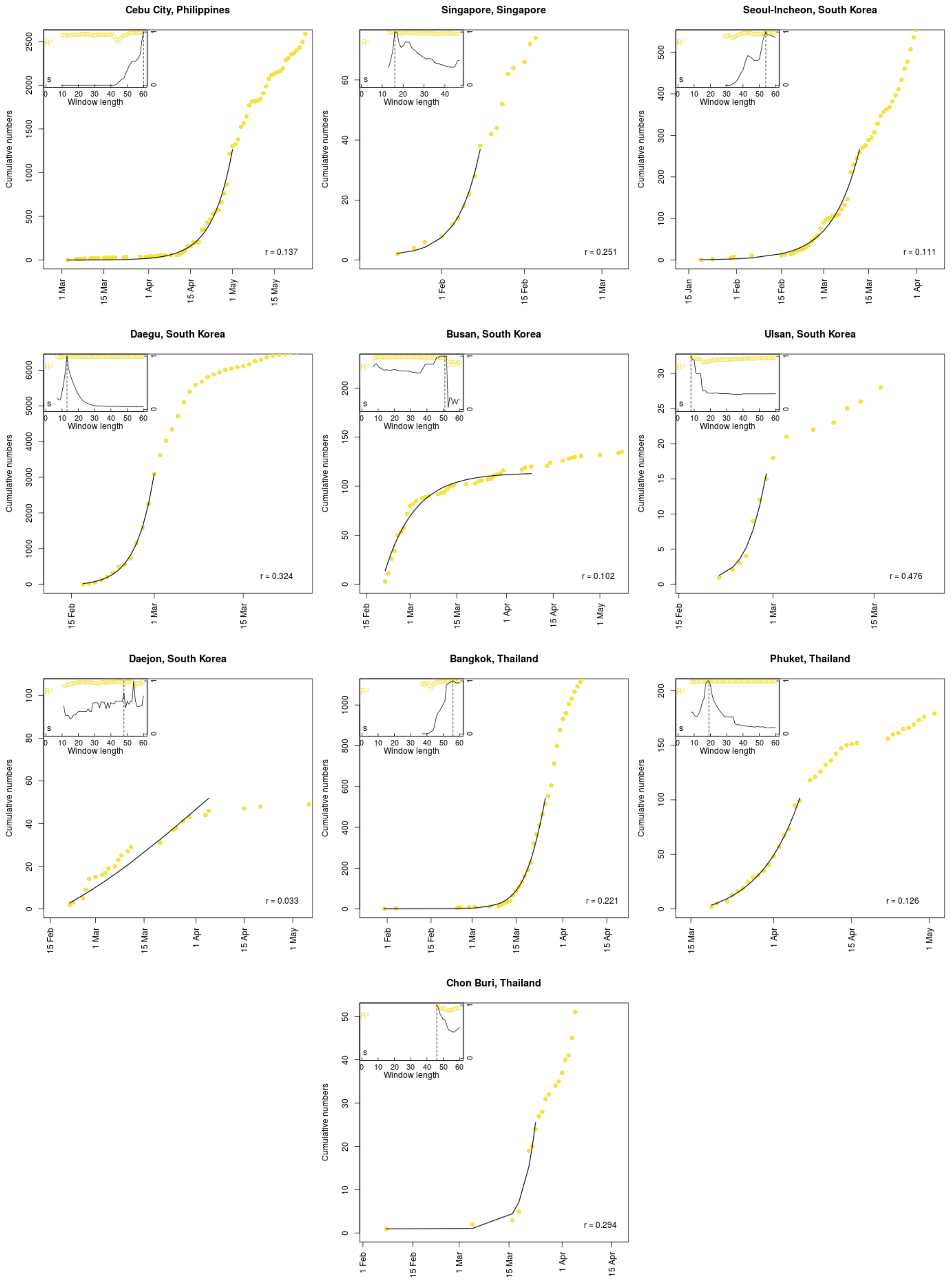
Cebu City, Philippines
r = 0.137
Singapore, Singapore
r = 0.251
Seoul-Incheon, South Korea
r = 0.111
Daegu, South Korea
r = 0.324
Busan, South Korea
r = 0.102
Ulsan, South Korea
r = 0.476
Daejon, South Korea
r = 0.033
Bangkok, Thailand
r = 0.221
Phuket, Thailand
r = 0.126
Chon Buri, Thailand
r = 0.294
Cumulative numbers
Window length
R²
s

S4.7 China

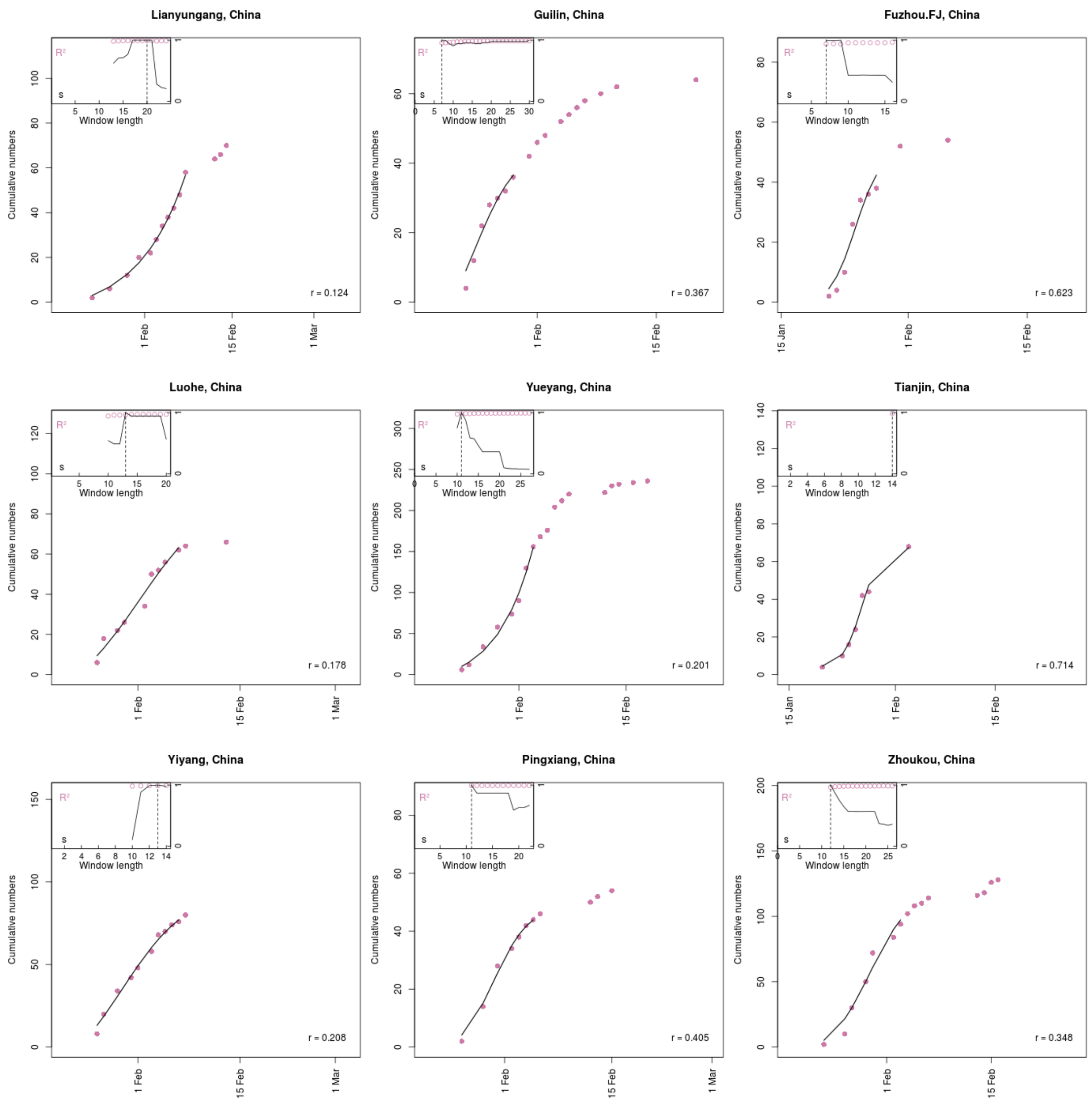
Lianyungang, China
r = 0.124
Guilin, China
r = 0.367
Fuzhou.FJ, China
r = 0.623
Luohe, China
r = 0.178
Yueyang, China
r = 0.201
Tianjin, China
r = 0.714
Yiyang, China
r = 0.208
Pingxiang, China
r = 0.405
Zhoukou, China
r = 0.348
Cumulative numbers
Window length
R^2
s
15 Jan
1 Feb
15 Feb
1 Mar

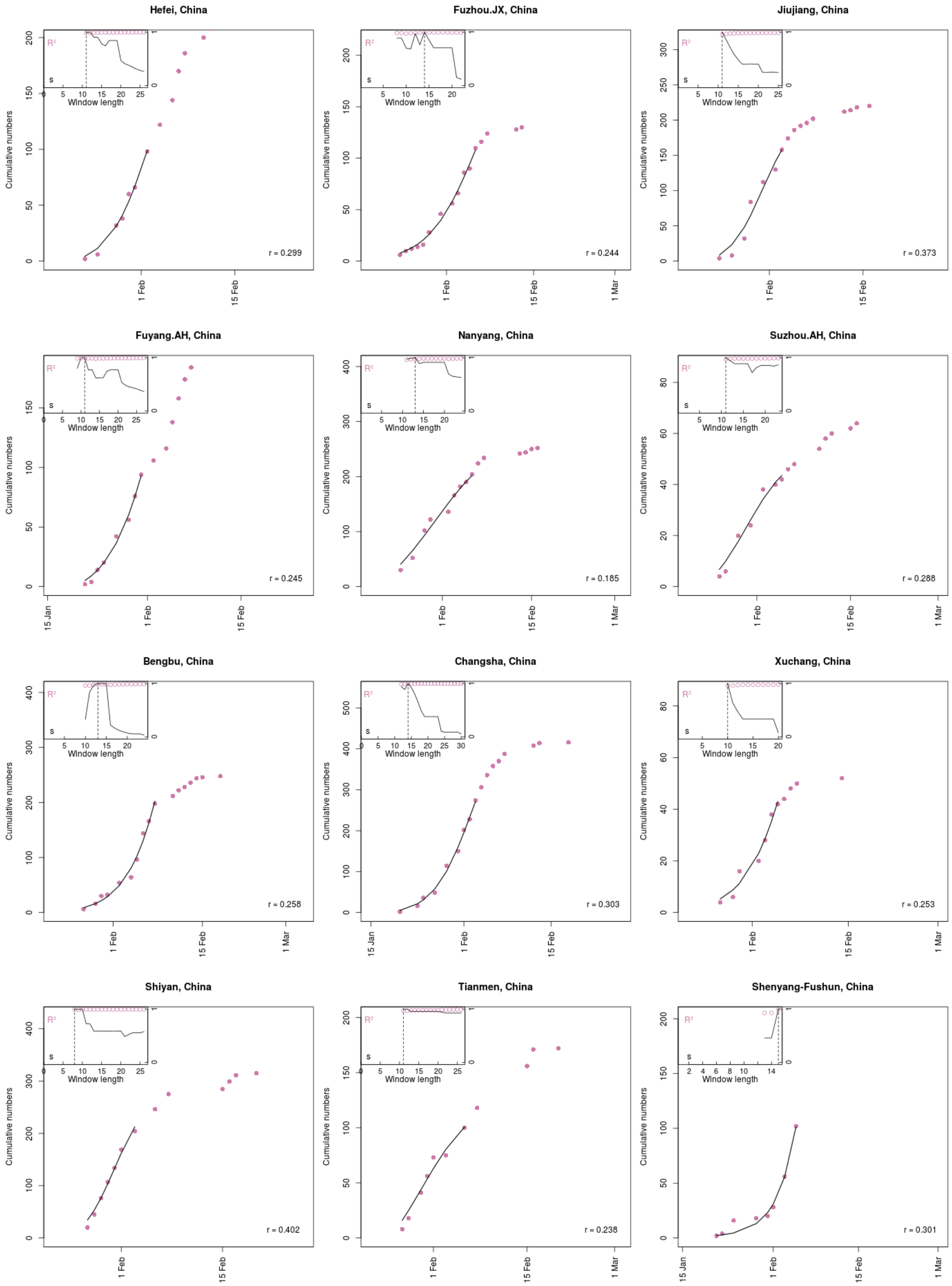
Hefei, China
r = 0.299
Fuzhou.JX, China
r = 0.244
Jiujiang, China
r = 0.373
Fuyang.AH, China
r = 0.245
Nanyang, China
r = 0.185
Suzhou.AH, China
r = 0.288
Bengbu, China
r = 0.258
Changsha, China
r = 0.303
Xuchang, China
r = 0.253
Shiyan, China
r = 0.402
Tianmen, China
r = 0.238
Shenyang-Fushun, China
r = 0.301
Cumulative numbers
Window length
R²
s
15 Jan
1 Feb
15 Feb
1 Mar

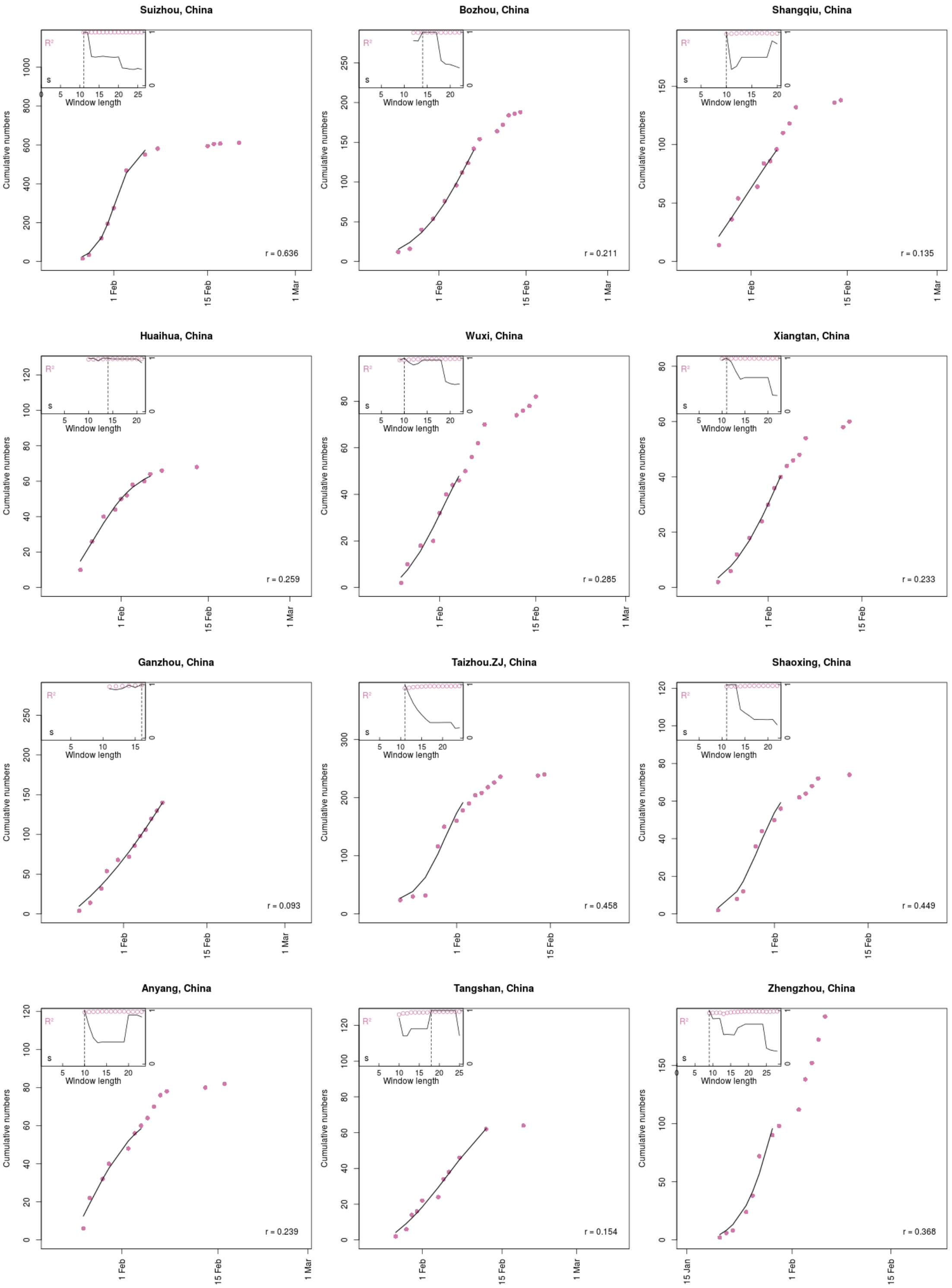
Suizhou, China
r = 0.636
Bozhou, China
r = 0.211
Shangqiu, China
r = 0.135
Huaihua, China
r = 0.259
Wuxi, China
r = 0.285
Xiangtan, China
r = 0.233
Ganzhou, China
r = 0.093
Taizhou.ZJ, China
r = 0.458
Shaoxing, China
r = 0.449
Anyang, China
r = 0.239
Tangshan, China
r = 0.154
Zhengzhou, China
r = 0.368
Cumulative numbers
Window length
R²
s
15 Jan
1 Feb
15 Feb
1 Mar

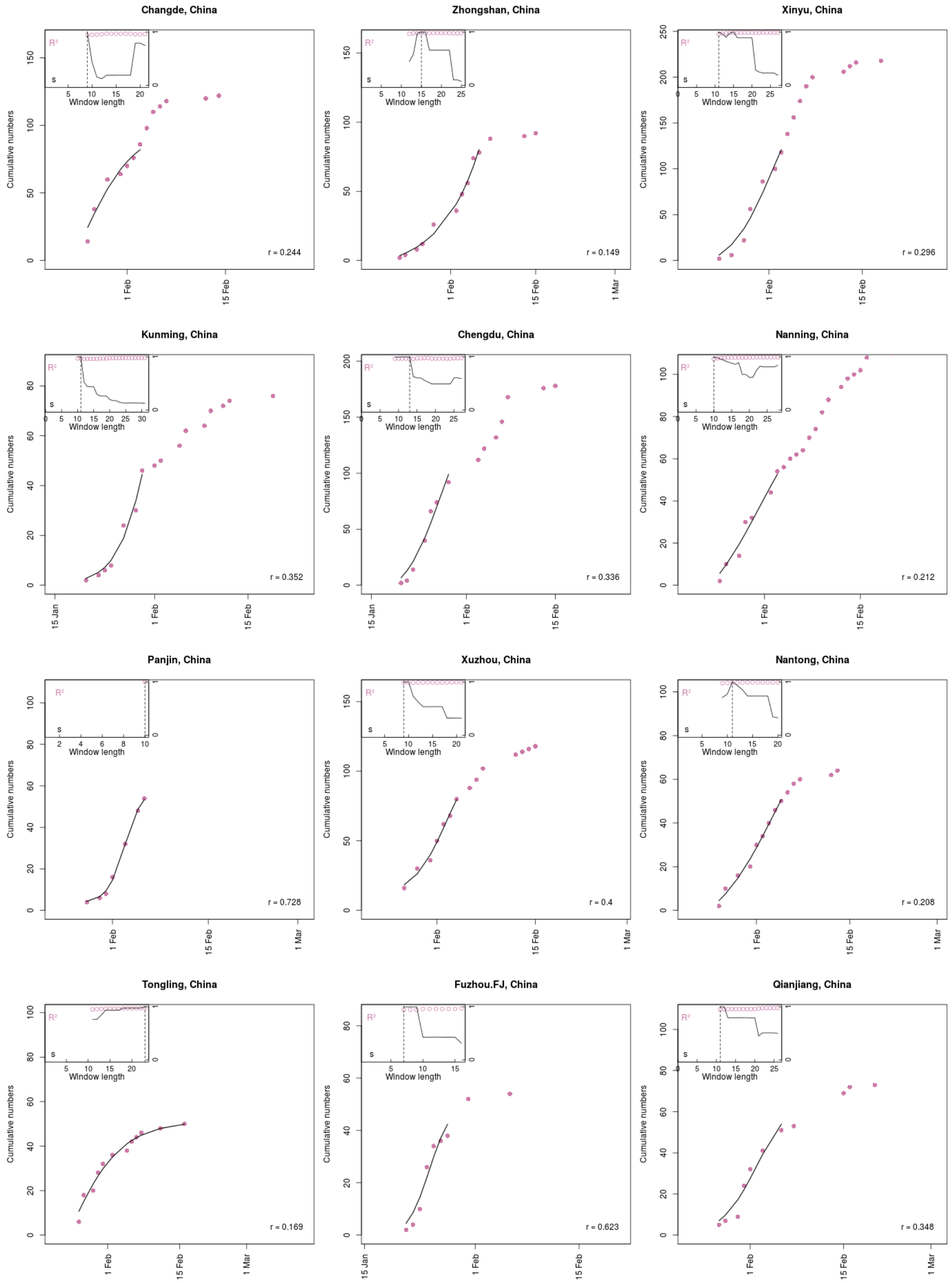
Changde, China
r = 0.244
Zhongshan, China
r = 0.149
Xinyu, China
r = 0.296
Kunming, China
r = 0.352
Chengdu, China
r = 0.336
Nanning, China
r = 0.212
Panjin, China
r = 0.728
Xuzhou, China
r = 0.4
Nantong, China
r = 0.208
Tongling, China
r = 0.169
Fuzhou.FJ, China
r = 0.623
Qianjiang, China
r = 0.348
Cumulative numbers
Window length
R²
s
15 Jan
1 Feb
15 Feb
1 Mar

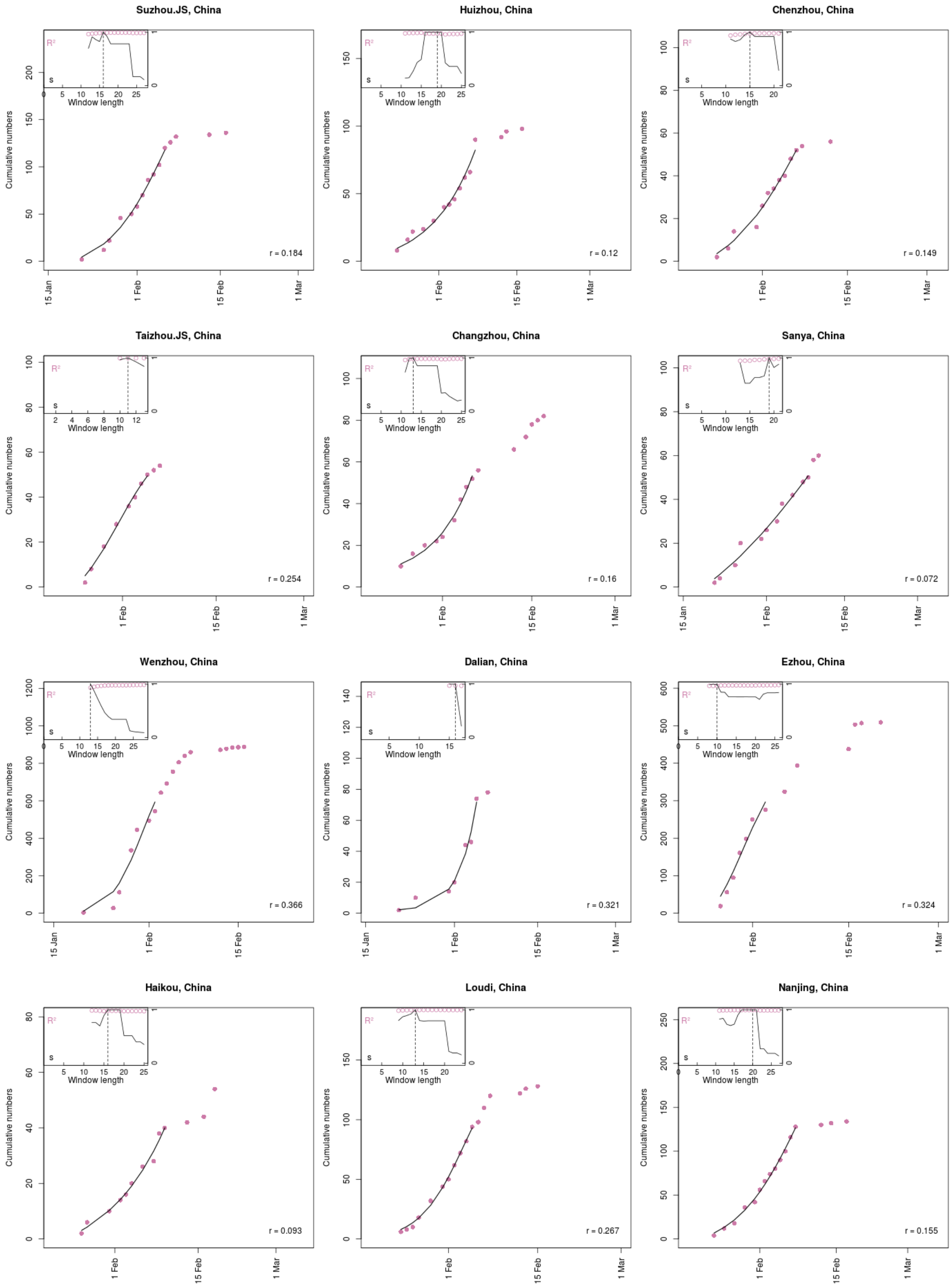
Suzhou.JS, China
r = 0.184
Huizhou, China
r = 0.12
Chenzhou, China
r = 0.149
Taizhou.JS, China
r = 0.254
Changzhou, China
r = 0.16
Sanya, China
r = 0.072
Wenzhou, China
r = 0.366
Dalian, China
r = 0.321
Ezhou, China
r = 0.324
Haikou, China
r = 0.093
Loudi, China
r = 0.267
Nanjing, China
r = 0.155
Cumulative numbers
Window length
R²
s
15 Jan
1 Feb
15 Feb
1 Mar

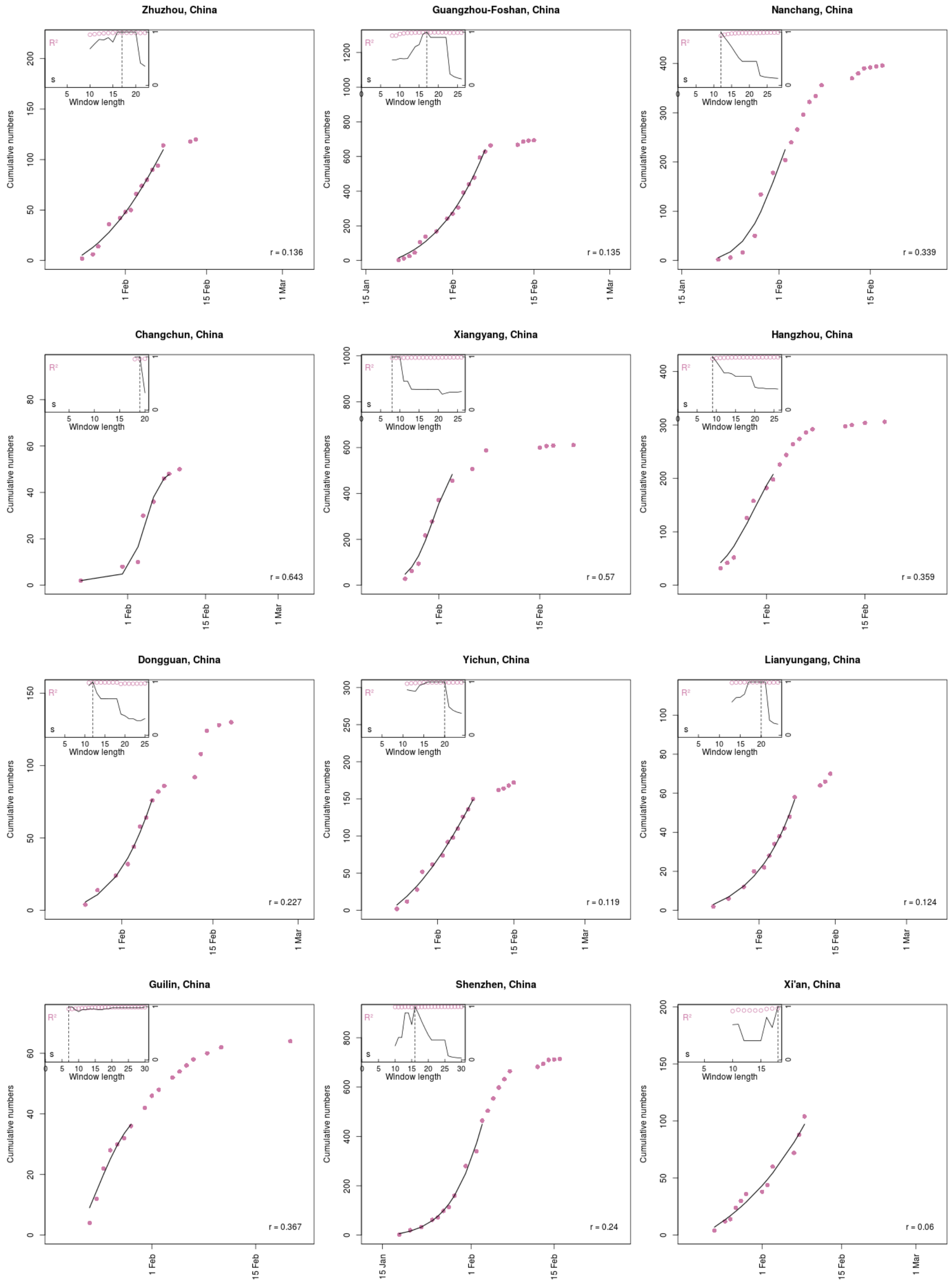
Zhuzhou, China
Guangzhou-Foshan, China
Nanchang, China
Changchun, China
Xiangyang, China
Hangzhou, China
Dongguan, China
Yichun, China
Lianyungang, China
Guilin, China
Shenzhen, China
Xi'an, China
Cumulative numbers
Window length
R²
s
r = 0.136
r = 0.135
r = 0.339
r = 0.643
r = 0.57
r = 0.359
r = 0.227
r = 0.119
r = 0.124
r = 0.367
r = 0.24
r = 0.06
15 Jan
1 Feb
15 Feb
1 Mar

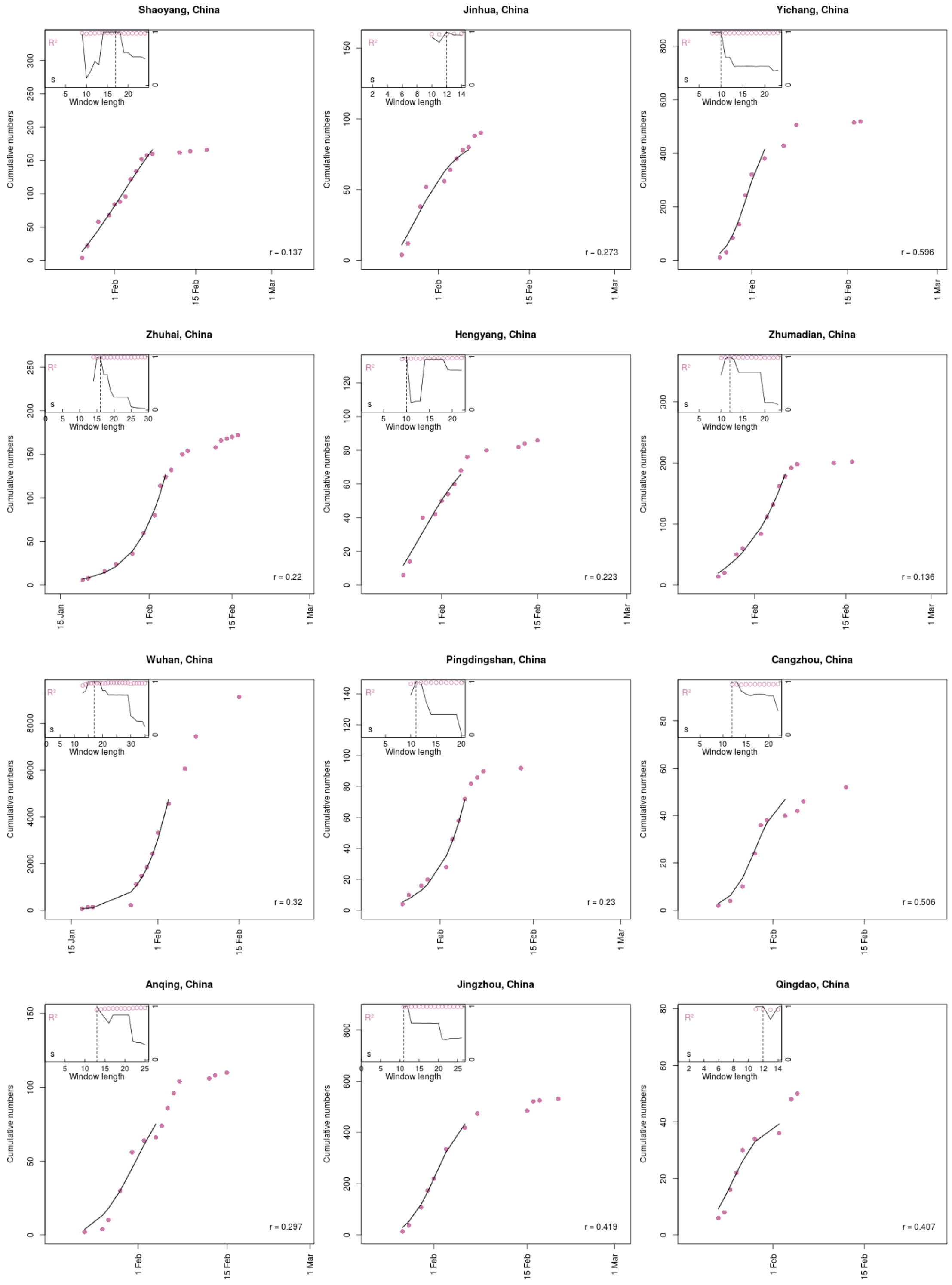
Shaoyang, China
r = 0.137
Jinhua, China
r = 0.273
Yichang, China
r = 0.596
Zhuhai, China
r = 0.22
Hengyang, China
r = 0.223
Zhumadian, China
r = 0.136
Wuhan, China
r = 0.32
Pingdingshan, China
r = 0.23
Cangzhou, China
r = 0.506
Anqing, China
r = 0.297
Jingzhou, China
r = 0.419
Qingdao, China
r = 0.407
Cumulative numbers
Window length

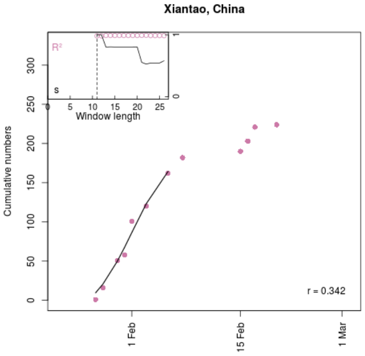
Xiantao, China
Cumulative numbers
0
50
100
150
200
250
300
1 Feb
15 Feb
1 Mar
r = 0.342
R²
s
Window length
0
5
10
15
20
25

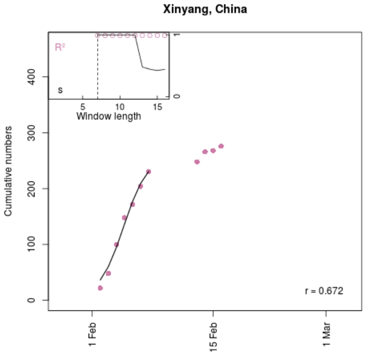
Xinyang, China
Cumulative numbers
0
100
200
300
400
1 Feb
15 Feb
1 Mar
r = 0.672
R²
s
Window length
5
10
15

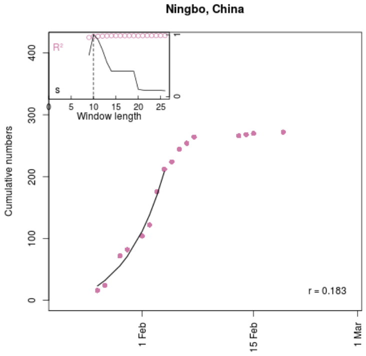
Ningbo, China
Cumulative numbers
0
100
200
300
400
1 Feb
15 Feb
1 Mar
r = 0.183
R²
s
Window length
0
5
10
15
20
25

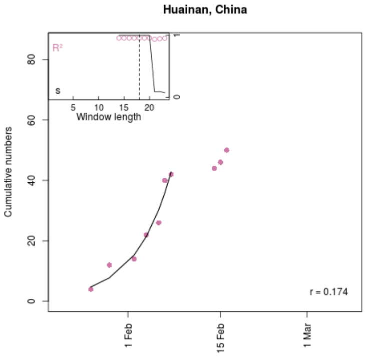
Huainan, China
Cumulative numbers
0
20
40
60
80
1 Feb
15 Feb
1 Mar
r = 0.174
R²
s
Window length
5
10
15
20

References

[1] Bates, D., Maechler, M., Bolker, B., Walker, S., Christensen, R. H. B., Singmann, H., Dai, B., Grothendieck, G., Green, P. and Bolker, M. B. [2015], 'Package 'lme4'', *Convergence* **12**(1), 2.

[2] *Central Intelligence Agency (CIA). The World Factbook* [2020]. https://www.cia.gov/library/publications/the-world-factbook/fields/342.html [Accessed on: 2020-05-07]

[3] Cox, W. [2020], 'Demographia World Urban Areas: 15th Annual Edition'. http://www.demographia.com/db-worldua.pdf [Accessed on: 2020-04-01]

[4] Czernecki, B., Głogowski, A. and Nowosad, J. [2020], 'Climate: An R Package to Access Free In-Situ Meteorological and Hydrological Datasets for Environmental Assessment', *Sustainability* **12**(1), 394.

[5] Du, Z., Xu, X., Wu, Y., Wang, L., Cowling, B. and Meyers, L. [2020], 'Serial interval of covid-19 among publicly reported confirmed cases', *Emerg Infect Dis* **26**(6), 1341–1343.

[6] Fox, J. and Weisberg, S. [2019], 'Using car Functions in Other Functions'.

[7] Ganyani, T., Kremer, C., Chen, D., Torneri, A., Faes, C., Wallinga, J. and Hens, N. [2020], 'Estimating the generation interval for coronavirus disease (COVID-19) based on symptom onset data, March 2020', *Eurosurveillance* **25**(17), 2000257.

[8] *Geonames* [2020]. www.geonames.org [Accessed on: 2020-06-03]

[9] *Global Burden of Disease Collaborative Network. Global Burden of Disease Study 2017 (GBD 2017) Results. Seattle, United States: Institute for Health Metrics and Evaluation (IHME)* [2018].

[10] *Google Inc. COVID-19 Community Mobility Reports* [2020]. https://www.google.com/covid19/mobility/ [Accessed on: 2020-12-01]

[11] Grömping, U. [2006], 'Relative importance for linear regression in R: The package relaimpo', *Journal of statistical software* **17**(1), 1–27.

[12] Grömping, U. [2007], 'Estimators of relative importance in linear regression based on variance decomposition', *The American Statistician* **61**(2), 139–147.

[13] Hale, T., Webster, S., Petherick, A., Phillips, T. and Kira, B. [2020], 'Oxford covid-19 government response tracker', *Blavatnik School of Government* **25**. https://www.bsg.ox.ac.uk/research/research-projects/coronavirus-government-response-tracker [Accessed on: 2020-12-01]

[14] Hersbach, H., Bell, B., Berrisford, P., Biavati, G., Horányi, A., Munoz Sabater, J., Nicolas, J., Peubey, C., Radu, R., Rozum, I., Schepers, D., Simmons, A., Soci, C., Dee, D. and Thépaut, J.-N. [2018], 'Era5 hourly data on single levels from 1979 to present', *Copernicus Climate Change Service (C3S) Climate Data Store (CDS)* . https://doi.org/10.24381/cds.adbb2d47 [Accessed on: 2020-11-30]

[15] King, A. A., Domenech de Cellès, M., Magpantay, F. M. G. and Rohani, P. [2015], 'Avoidable errors in the modelling of outbreaks of emerging pathogens, with special reference to Ebola', *Proceedings of the Royal Society B: Biological Sciences* **282**(1806), 20150347.

[16] Li, Q., Guan, X., Wu, P., Wang, X., Zhou, L., Tong, Y., Ren, R., Leung, K. S. M., Lau, E. H. Y., Wong, J. Y., Xing, X., Xiang, N., Wu, Y., Li, C., Chen, Q., Li, D., Liu, T., Zhao, J., Liu, M., Tu, W., Chen, C., Jin, L., Yang, R., Wang, Q., Zhou, S., Wang, R., Liu, H., Luo, Y., Liu, Y., Shao, G., Li, H., Tao, Z., Yang, Y., Deng, Z., Liu, B., Ma, Z., Zhang, Y., Shi, G., Lam, T. T. Y., Wu, J. T., Gao, G. F., Cowling, B. J., Yang, B., Leung, G. M. and Feng, Z. [2020], 'Early transmission dynamics in wuhan, china, of novel coronavirus-infected pneumonia', *The New England journal of medicine* **382**(13), 1199–1207.

[17] Ma, J. [2020], 'Estimating epidemic exponential growth rate and basic reproduction number', *Infectious Disease Modelling* **5**, 129–141.

[18] Ma, J., Dushoff, J., Bolker, B. M. and Earn, D. J. D. [2014], 'Estimating Initial Epidemic Growth Rates', *Bulletin of Mathematical Biology* **76**(1), 245–260.

[19] Nishiura, H., Linton, N. M. and Akhmetzhanov, A. R. [2020], 'Serial interval of novel coronavirus (COVID-19) infections', *International journal of infectious diseases* .

[20] *OGIMET* [2020]. www.ogimet.com [Accessed on: 2020-06-03]

[21] *Organisation for Economic Co-Operation and Development (OECD). OECD Metropolitan Database* [2020]. https://stats.oecd.org/Index.aspx?Datasetcode=CITIES [Accessed on: 2020-04-08]

[22] R Core Team [2019], 'R: A language and environment for statistical computing', R Foundation for Statistical Computing. https://www.R-project.org/

[23] Stekhoven, D. J. and Bühlmann, P. [2012], 'Missforest—non-parametric missing value imputation for mixed-type data', *Bioinformatics* **28**(1), 112–118.

[24] Wallinga, J. and Lipsitch, M. [2007], 'How generation intervals shape the relationship between growth rates and reproductive numbers', *Proceedings of the Royal Society B: Biological Sciences* **274**(1609), 599–604.

[25] *World Bank. World Development Indicators Database* [2020]. https://datacatalog.worldbank.org/dataset/world-development-indicators [Accessed on: 2020-05-08]

[26] *World Health Organisation (WHO). Electronic State Parties Self-Assessment Annual Reporting Tool* [2020]. https://extranet.who.int/e-spar/ [Accessed on: 2020-05-08]

[27] *World Health Organisation (WHO). Global Health Observatory Data Repository* [2020]. https://apps.who.int/gho/data/node.main.688 [Accessed on: 2020-04-08]

[28] Wu, J., Leung, K. and Leung, G. [2020], 'Nowcasting and forecasting the potential domestic and international spread of the 2019-ncov outbreak originating in wuhan, china: a modelling study', *The Lancet* **395**, 689–697.

[29] Xu, B., Kraemer, M. U., Gutierrez, B., Mekaru, S., Sewalk, K., Loskill, A., Wang, L., Cohn, E., Hill, S. and Zarebski, A. [2020], 'Open access epidemiological data from the COVID-19 outbreak', *The Lancet Infectious Diseases* **20**(5), 534.
